# Photothermal conversion triggered thermal asymmetric catalysis within metal nanoparticles loaded homochiral covalent organic framework

**DOI:** 10.1038/s41467-019-11355-x

**Published:** 2019-07-29

**Authors:** Hui-Chao Ma, Chen-Chen Zhao, Gong-Jun Chen, Yu-Bin Dong

**Affiliations:** College of Chemistry, Chemical Engineering and Materials Science, Collaborative Innovation Center of Functionalized Probes for Chemical Imaging in Universities of Shandong, Key Laboratory of Molecular and Nano Probes, Ministry of Education, Shandong Normal University, Jinan, 250014 P. R. China

**Keywords:** Heterogeneous catalysis, Metal-organic frameworks, Asymmetric catalysis

## Abstract

For seeking high enantiopurity, the previously reported thermal asymmetric catalysis is usually carried out at low temperature sometimes with limited yield, that is, the high enantiomeric excess (ee) usually at the cost of high yield. Thus, the achieving both high stereoselectivity and yield is an enormous challenge. We report herein two metal nanoparticle (M NP)-loaded and porphyrin-containing homochiral covalent organic framework (CCOF)-based composite catalysts, and their application in the thermally-driven asymmetric one-pot Henry and A^3^-coupling reactions. All the reactions are conducted at elevated temperatures with both excellent stereoselectivity and yield which resulted from the synergy of CCOF confinement effect and M NP catalytic activation. Notably, the needed thermal energy for the asymmetric reactions herein is derived from the photothermal conversion via porphyrin-based CCOF upon irradiation with visible light. Remarkably, the CCOF confinement effect can be effectively maintained up to 100 °C for the asymmetric one-pot Henry and A^3^-coupling reactions herein.

## Abstract (web summary)

Achieving both high stereoselectivity and yield is a challenge for conventional asymmetric catalysis. Here, the authors report two metal nanoparticle-loaded and porphyrin-containing homochiral covalent organic framework-based composite catalysts that exhibit high stereoselectivity and yield in the thermally-driven asymmetric reactions.

## Introduction

Photothermal conversion materials (PTCMs), which can transfer light energy into thermal energy, exhibit a variety of potential applications involved photothermal conversion process. For example, PTCMs have been successfully used in the fields of solar collection^1^, microcontrollers^2^, and especially photothermal therapy (PTT) for cancer treatment^3^. Among various applications, PTCMs-based photothermal chiral catalysis, especially natural sunlight triggered thermally-driven asymmetric synthesis, has attracted much less attention^4^.

As it is known, both yield and enantioselectivity are the crucial indicators for the chiral synthesis, and the achieving high yield with excellent enantiomeric excess (ee) is meaningful, especially for those important asymmetric organic transformations, such as asymmetric Henry and benzaldehyde–phenylacetylene–pyrrolidine A^3^-coupling reactions, that can transform the achiral compounds into the high value-added chiral organic chemicals. For seeking high enantiopurity, the most reported high-performance and thermally-driven asymmetric reactions, however, were generally performed at relatively low temperature, which often caused a low product yield. For example, the recently reported asymmetric Henry reaction between benzaldehyde and nitromethane was carried out in a temperature range of −78–45 °C with 28–99% yields and 17–99% ee^5–25^.

On the other hand, homogenous catalysis has been the main theme in asymmetric synthesis, but it often involved noble metals that cannot be efficiently recycled during the catalytic process. Therefore, the precious metal-based reusable heterogeneous asymmetric catalytic systems are imperative due to more and more serious sustainable and global environmental issues^26^. Up to date, however, only a few heterogeneous catalytic enantioselective Henry reactions were reported. For instance, the chiral cyclodextrin^27^ and aerogel-supported MgO with chiral binol^28^ were used to promote the benzaldehyde-nitromethane addition in high 98 (90% ee) and 95% (90% ee) yields, but they are conducted at −20 and −78 °C, respectively. In addition, a chiral MOF-catalyzed benzaldehyde-nitromethane addition at 45 °C was also reported^29^, and the desired product was obtained in high 95% ee albeit with only 71% yield.

In addition, the traditional asymmetric catalytic systems are normally complicated, and various additives such as cocatalysts are often coexisted^30^. For example, the optically active pyrrolidine-derived propargylamine generated from the homogeneous CuI-catalyzed benzaldehyde–phenylacetylene–pyrrolidine A^3^-coupling can be achieved in high 92% yield and 92% ee, but with the aid of the chiral acid-thiourea cocatalysts and molecular sieve at 0 °C^31^.

For meeting the multifaceted requirements of the thermally-driven asymmetric catalysis, we report herein two metal nanoparticle (M NP)-loaded and homochiral covalent organic framework (CCOF)-based asymmetric catalytic materials, termed as M@CCOF-CuTPP (M=Au (**2**), Pd (**3**)), which are composed of a porphyrin-derived CCOF (CCOF-CuTPP (**1**)) and corresponding M NP. The obtained **2** and **3** feature chiral templating, photothermal conversion and highly catalytic activity, which allow them to highly promote the thermally-driven asymmetric one-pot Henry and A^3^-coupling reactions under photothermal conversion conditions, respectively.

## Results

### Synthesis and characterization of 1–3

**Fig. 1:**
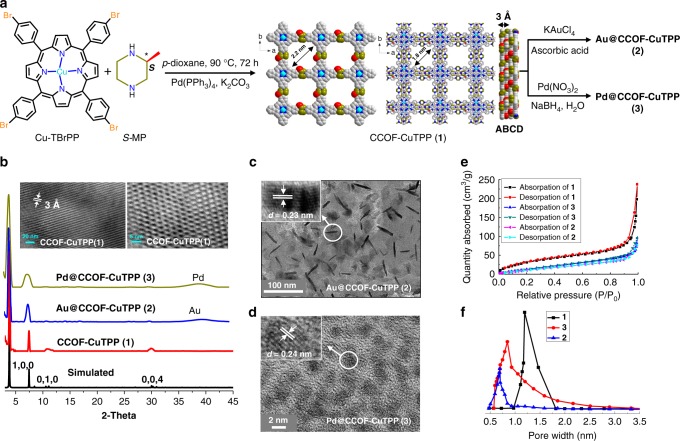
Synthesis and characterization of **1–3**. **a** Synthesis of **1–3**. Single 2D layer and crystal packing patterns of **1**. **b** Simulated and measured PXRD patterns of CCOF-CuTPP (**1**), Au@CCOF-CuTPP (**2**) and Pd@CCOF-CuTPP (**3**). TEM images (side and top views) of **1** were inserted. **c**, **d** TEM images of **2** and **3**. Their SEM images are shown in Supplementary Fig. 1. **e** N_2_ adsorption isotherms of **1–3** at 77 K. **f** The pore widths of **1–3** are centered at 1.20, 0.71, and 0.85 nm, respectively

As shown in Fig. 1a, the homochiral organic framework of CCOF-CuTPP (**1**) was prepared by directly assembling the chiral organic component of *S*-(+)−2-methylpiperazine (*S*-MP) and photothermal conversion species of copper tetrabromophenolphthalein (Cu-TBrPP) in anhydrous *p*-dioxane (90 °C, 72 h) with the aid of Pd(PPh_4_)_3_ and K_2_CO_3_ in 63% yield. After filtration and successively and completely washed with hydrochloric acid, dichloromethane, ethanol, and water, **1** was obtained as purple-black crystalline powders that were insoluble in common organic solvents and water.

Scanning electron microscopy (SEM) was used to visualize the as-synthesized **1**, and its particle morphology was observed (Supplementary Fig. 1). Thermogravimetric analysis (TGA) indicated that no weight loss of **1** was observed up to ca. 350 °C (Supplementary Fig. 2). In addition, IR (Supplementary Fig. 3) and ^13^C CP-MAS (Supplementary Fig. 4) were also used to establish the connectivity of the COF species and the existence of Cu-TPP and *S*-MP moieties in **1** were directly evidenced. Notably, **1** shows good crystallinity as revealed by its powder X-ray diffraction (PXRD) pattern (Fig. 1b). The structural modeling was thus conducted with the software of Materials Studio (ver. 2018) (Supplementary Fig. 5). The most probable structure of **1** was simulated, analogous to that of **1** as a 2D staggered layered-sheets using the chiral space group of *P*_41_ with the optimized parameters of *a* *=* *b* = 22.073 Å and *c* *=* 12.000 Å (residuals wRp = 3.72% and Rp = 2.89%, Supplementary Table 1). As indicated in Fig. 1b, the intense PXRD peaks at 3.9, 8.3 and 29.6° displayed by **1** correspond to the (1,0,0), (0,1,0) and (0,0,4) planes, respectively. The structural modeling shows that the Cu-TPP is linked together via *S*-MP into a 2D layer extended in the crystallographic *ab* plane with square-like cavity, in which the diagonal C···C distance is ca. 2.2 nm (Fig. 1a). These layers further stack together in an ABCD-fashion to form tetragonal channels with a reduced pore size (diagonal C···C distance at ca. 1.8 nm) due to their stagger arrangement (Fig. 1a). The interlayer distance of 3.0 Å met the low-energy minimum, which is consistent with the (0,0,4) diffraction peak at 29.6°, indicating that the adjacent layers in **1** are in π-π contact. Transmission electron microscopy (TEM) images clearly revealed that **1** is a 2D layered structure (*d*_interlayer_ = 3 Å) which contains tetragonal pore with a ca. 1.8 nm diameter (Fig. 1b, inset), which is well consistent with its structural modeling analysis. The reported 2D COFs usually favored AA-^32^, AB-^32^ and ABC-stacking modes^33^, and the ABCD stacking mode was uncommon. **1** herein with other stacking modes, however, gave the PXRD patterns that largely deviated from the experimentally observed profile (Supplementary Fig. 5). In addition, **1** crystalized in *P*_41_ space group with ABCD stacking mode possessed the lowest total energy (221.93 kcal/mol) among other types of possible simulated space groups (*P*_21_ 285.50 kcal/mol, *P*_1_ 344.74 kcal/mol) with different stacking modes (Supplementary Tables 2–3), implying that the ABCD stacking mode is more energetically favored. Furthermore, it seems that the CuTBrPP monomer played an important role in the crystallization of **1**. If the metal-free TBrPP instead of CuTBrPP was used to perform the COF synthesis under the same reaction conditions, the COF with the decreased crystallinity was obtained (Supplementary Fig. 5).

Au@CCOF-CuTPP (**2**) and Pd@CCOF-CuTPP (**3**) were prepared by successive solution impregnating and metal reduction steps (Fig. 1a). As shown in Fig. 1b, the framework of **1** remained intact during the M NP loading process. The diffraction of 2*θ* values at 39 and 40^o^ in PXRD confirmed the existence of Au(0) in **2** and Pd(0) in **3** (Fig. 1b), which was further supported by X-ray photoelectron spectroscopy (XPS). However, no valence change of Cu(II) in **2** and **3** was detected (Supplementary Figs. 6–7)^34^. The uploaded Au and Pd amount, as determined by inductively coupled plasma (ICP) measurement, was up to 17.9 and 24.8 wt%, respectively. TEM showed that the Au NR (mean width and length ca. 3 and 30 nm) and Pd NP (particle size ca. 2–5 nm) were highly dispersed in the CCOF matrix, and their characteristic atomic lattice fringes (*d*_interplanar spacing_ = 0.23 nm for Au and *d*_interplanar spacing_ = 0.24 nm for Pd) were clearly observed^35,36^(Fig. 1c, d). In addition, the porosity difference before and after M NP loading was demonstrated by the gas adsorption-desorption measurement. As shown in Fig. 1e, N_2_ absorption amount of **1**–**3** at 77 K is 237.9, 79.4 and 94.8 cm^3^ g^−1^, respectively. Their corresponding surface areas calculated on basis of the BET model are 686 (**1**), 95 (**2**), and 101 m^2^ g^−1^ (**3**), respectively. The decreased N_2_ adsorption and the reduced surface area in **2** and **3** were obviously caused by the M NP loading. The pore size distribution curves, calculated from Barrett-Joyner-Halenda analysis, indicated that **1**–**3** possessed the narrow pore diameter distributions (Fig. 1f). Owing to the existence of chiral *S*-MP, **1**–**3** herein are optical active. For **1**, it displayed positive Cotton effect at 450 nm and negative dichroic signal at 230 nm; for **2** and **3**, they all exhibited positive Cotton effect at 445 nm and negative dichroic signals at 220 nm in their CD spectra (Supplementary Fig. 8).

### Catalytic activity of 2 and 3 under photothermal conditions

The catalytic activity of Au NR loaded **2** was preliminarily examined by the one-pot asymmetric Henry reaction starting from benzyl alcohol with nitromethane. As is known, the optically active *β*-nitro alcohols generated from the asymmetric Henry reaction are important intermediates for the synthesis of various valuable chiral chemicals, such as bioactive natural products and pharmaceutical agents^37,38^.

The yield and ee for all reactions, together with the control experiments, are displayed in Table 1 (entries 1–12). Catalytic reactions were performed under various conditions, including different solvents (Table 1, entries 1–4) and catalysts with or without visible light irradiation at different temperatures (Table 1, entries 5–9). The best result was observed when the reaction was conducted in toluene/EtOH (1:1) at room temperature for 10 h (Table 1, entry 1) with **2** (1 mol% Au, lower catalyst loading would lead lower yield) upon illumination with visible light (*λ* > 400 nm). The nitroaldol was obtained in excellent 98% yield (TON = 98, TOF = 9.8 h^−1^) with excellent enantiomeric excess (98% ee). The measured reaction solution temperature was 58 °C, implying that the porphyrin-derived **2** is a good COF-based PTCM. A series of control experiments were performed to further confirm this **2**-promoted one-pot asymmetric Henry reaction was a photoinduced thermally-driven one (Table 1, entries 5–6), in which **1** was responsible for not only chiral confinement and photothermal conversion, but also partial catalytic function (Table 1, entries 7–8). The partially catalytic function of **1** resulted from the CuTPP building block in **1**, which was further confirmed by the catalytic test performed on the CuTBrPP monomer (Table 1, entry 9). The loaded Au NR, however, served as the dominated catalytic sites (Table 1, entry 10, Supplementary Fig. 11). In contrast, the Au@amorphous polymer generated from Cu-TBrPP, *S*-MP and Au NR provided a very low 8% ee and a 73 % yield (Table 1, entry 11, Supplementary Fig. 11), which demonstrated the crystalline CCOF displayed much better chiral templating and catalytic performance. It is noteworthy that this one-pot asymmetric Henry reaction could even significantly occurred under natural sunlight irradiation, and the desired nitroaldol was obtained in moderate yield (49%) with excellent stereoselectivity (96% ee) (Table 1, entry 12).

**Table 1:** Optimization of the **2**-catalyzed model one-pot asymmetric Henry reaction^a^


Entry	Catalyst	Solvent	T(^°^C)/*hv*	Yield (ee) %^b^
1	**2** (1 mol Au %)	PhMe/EtOH	r.t./*hv*	98 (98)
2	**2** (1 mol Au %)	PhMe	r.t./*hv*	12 (95)
3	**2** (1 mol Au %)	EtOH	r.t./*hv*	8 (92)
4	**2** (1 mol Au %)	CH_3_CN	r.t./*hv*	14 (96)
5	**2** (1 mol Au %)	PhMe/EtOH	58 °C/dark	98 (91)
6	**2** (1 mol Au %)	PhMe/EtOH	r.t./dark	− (−)
7	**1** (1 mol%)	PhMe/EtOH	58 °C/dark	45 (94)
8	**1** (1 mol %)	PhMe/EtOH	r.t./*hv*	40 (92)
9	Cu-TBrPP monomer	PhMe/EtOH	r.t./*hv*	43 (−)
10	Au NR (1 mol %)	PhMe/EtOH	58 °C/dark	97 (−)
11	Au@amorphous polymer (1 mol% Au %)	PhMe/EtOH	r.t./*hv*	73 (18)
12	**2** (1 mol Au %)	PhMe/EtOH	r.t./solar light	49 (96)

^a^Reaction conditions: **2** (6.0 mg, 1 mol% Au equiv), benzyl alcohol (0.5 mmol), nitromethane (1.5 mmol), K_2_CO_3_ (1.5 mmol), PhMe/EtOH (1:1, 2 mL), 300 W xenon with a power density of 2.5 W cm^−2^ (*λ* *>* 400 nm), 10 h, in air

^b^Yield was determined by the GC on HP-5 column, and ee was determined by HPLC with a Chiralcel OD-H column (90: 10 = *n*-hexane: isopropanol, 1.0 mL min^−1^, 230 nm) (Supplementary Figs. 9, 10). Because no one-pot tandem asymmetric Henry reaction starting from benzyl alcohol with nitromethane was reported so far, so only the comparison of **2** with reported asymmetric Henry reactions between benzaldehyde and nitromethane was shown in Supplementary Table 4

The first benzyl alcohol selective oxidation step was separately examined (Supplementary Figs. 12–13). The result showed that the benzyl alcohol was selectively oxidized to benzaldehyde in PhMe/EtOH (1:1) with the aid of **2** in 98% yield under the given conditions, which ensured that the whole one-pot tandem reaction proceeded smoothly. As a typical heterogeneous catalyst (Supplementary Fig. 14), **2** can be reused in this one-pot tandem Henry reaction and the yield was still up to 93% (95% ee) even after five catalytic cycles without loss its crystallinity and structural integrity (Supplementary Figs. 15–18). The leaching amount of Au in **2** is only 15.6% after five catalytic cycles which was determined by ICP-AES.

With the optimized conditions in hand, we investigated the scope of **2**-catalyzed one-pot Henry reactions utilizing various substrates. As shown in Table 2, the substituted benzyl alcohols with either electron-donating or electron-withdrawing groups at different substituted positions under the optimized conditions furnished the excellent yields (93–99 %) with the excellent ee values (94–98% ee, entries 1–9). Meanwhile, the larger sized 9H-fluorene-2-methanol (5.06 × 10.66 Å, Supplementary Fig. 20) and 9-anthracenemethanol (7.13 × 8.92 Å, Supplementary Fig. 20) substrates (Table 2, entries 10–11) were unfavorable for the reaction, and the corresponding products were obtained in low yields (6–15%) but with 97% ee. As shown above, the pore size of **2** was centered at 0.71 nm, so these larger sized aromatic alcohols were not easy to enter the COF cavity, consequently, led to a low yield. On the other hand, this indicated that the one-pot asymmetric reaction should be an inner pore catalytic process. Notably, the generated products featured *R* configuration.

**Table 2:** Scope of the **2**-catalyzed one-pot asymmetric Henry reactions^a^


Entry	Ar-CH_2_OH	Yield (%)^b^	ee (%)^b^	TON
1	PhCH_2_OH	98	98 (*R*)	98
2	4-Me-PhCH_2_OH	97	98 (*R*)	97
3	3-Me-PhCH_2_OH	98	95 (*R*)	98
4	4-MeO-PhCH_2_OH	99	96 (*R*)	99
5	3-MeO-PhCH_2_OH	99	94 (*R*)	99
6	4-NO_2_-PhCH_2_OH	94	97 (*R*)	94
7	3-NO_2_-PhCH_2_OH	95	95 (*R*)	95
8	4-F-PhCH_2_OH	93	94 (*R*)	93
9	4-Cl-PhCH_2_OH	95	98 (*R*)	95
10	9H-Fluorene-2-methanol	6	97 (*R*)	6
11	9-Anthracenemethanol	15	97 (*R*)	15

^a^Reaction conditions: **2** (6 mg, 1 mol% Au equiv), aromatic alcohol (0.5 mmol), nitromethane (1.5 mmol), K_2_CO_3_ (1.5 mmol), PhMe/EtOH (1:1, 2 mL), room temperature, irradiated by 300 W xenon with a power density of 2.5 W cm^−2^ (*λ* *>* 400 nm), 10 h. Yields and ee are determined by GC and chiral HPLC analysis, respectively (Supplementary Fig. 19)

For demonstrating the generality of M NPs-loaded and CCOF-based asymmetric photothermal catalysis, we next evaluated the **3**-catalyzed asymmetric A^3^-coupling (aldehyde-alkyne-amine) reaction, which is well-known a typical thermally-driven one-pot multicomponent reaction and the generated propargylamines are the important intermediates for the synthesis of various value-added nitrogen-containing compounds^39,40^.

Optimization of A^3^-coupling was based on the one-pot benzaldehyde-pyrrolidine-phenylacetylene (mole ratio, 1.0:1.0:1.5) coupling with or without visible light irradiation and heating (Table 3, entries 1–15). As shown in Table 3, various organic solvents such as *p*-dioxane, toluene, acetonitrile, and ethanol were utilized to perform the A^3^-coupling model reaction (Table 3, entries 1–4). Upon illumination by visible light (*λ* > 400 nm), the ideal result was obtained when the reaction was carried out in *p*-dioxane at room temperature for 10 h (Table 3, entries 1 and 5) in the presence of **3** with 2.1 or 3.2 mol% Pd loading. The reaction provided the pyrrolidine-derived propargylamine in 98% yield with an excellent enantiomeric excess (96% ee). The measured reaction system temperature was 45 °C under the given conditions. In addition, when the reaction was conducted in *p*-dioxane with a lower 1.4 mol% Pd loading, the coupling product was obtained in slightly lower 90% yield (97% ee) (Table 3, entry 6). For confirming the A^3^-coupling herein was a thermally-driven reaction, the reaction was carried out at 45 °C with **3** (2.1 mol% Pd) in the dark (Table 3, entry 7), and a good coupling yield (90%) with excellent 96% ee value was observed. In contrast, only 21% coupling yield (90% ee) was obtained once the reaction was performed with **3** in the dark at room temperature (Table 3, entry 8). In addition, a moderate yield (55%) with high enantioselectivity (92% ee) was observed when the reaction was conducted with **1** under visible light irradiation (Table 3, entry 9), which implied the catalytic activity coming from the synergy effect of **1** and loaded Pd NP. This was further proved by the reactions with **1** (Table 3, entry 10) and CuTBrPP (Table 3, entry 11) conducted at 45 °C in the dark and illuminated by visible light, respectively. Besides catalytic activity, the chiral templating effect of **1** was well demonstrated by the control experiments, as shown in Table 3 (entries 12 and 13). Without the aid of **1**, the coupled product was isolated in 17% yield by visible light irradiation (Table 3, entry 12) or 67% yield on heating treatment (Table 3, entry 13) in the presence of only Pd NP, but without any effective enantioselectivity. It is similar to **2**-catalyzed one-pot Henry reaction, this **3**-catalyzed A^3^-coupling even significantly proceeded under natural sunlight irradiation, and the desired propargylamine was generated in moderate 45% yield with excellent 92% ee after 10 h at ambient temperature (Table 3, entry 14). Again, no desirable yield and stereoselectivity were observed when the Pd@amorphous polymer formed by Cu-TBrPP, *S*-MP, and Pd NP was used instead of **3** to perform the reaction (Table 3, entry 15), further demonstrated the importance of the highly crystalline CCOF in this asymmetric A^3^-coupling reaction.

**Table 3:** Optimization of the **3**-catalyzed asymmetric A^3^-coupling reaction^a^


Entry	Cat.	Solvent	T (°C)/*hv*	Yield (ee) %^b^
1	**3** (2.1 mol Pd %)	*p*-dioxane	r.t./*hv*	98 (96)
2	**3** (2.1 mol Pd %)	Toluene	r.t./*hv*	81 (89)
3	**3** (2.1 mol Pd %)	EtOH	r.t./*hv*	58 (92)
4	**3** (2.1 mol Pd %)	CH_3_CN	r.t./*hv*	70 (96)
5	**3** (2.8 mol Pd %)	*p*-dioxane	r.t./hv	98 (96)
6	**3** (1.4 mol Pd %)	*p*-dioxane	r.t./*hv*	90 (97)
7	**3** (2.1 mol Pd %)	*p*-dioxane	45 °C/dark	90 (96)
8	**3** (2.1 mol Pd %)	*p*-dioxane	r.t./dark	21 (90)
9	**1** (1.0 mol %)	*p*-dioxane	r.t./*hv*	55 (92)
10	**1** (1.0 mol %)	*p*-dioxane	45 °C/dark	50 (87)
11	Cu-TBrPP	*p*-dioxane	r.t./*hv*	46 (−)
12	Pd NPs (5.0 mol %)	*p*-dioxane	r.t./*hv*	17 (−)
13	Pd NPs (5.0 mol %)	*p*-dioxane	45 °C/dark	67 (−)
14	**3** (2.1 mol Pd %)	*p*-dioxane	r.t./sunlight	45 (92)
15	Pd@amorphous polymer (2.1 mol% Pd %)	*p*-dioxane	r.t./*hv*	78 (14)

^a^Reaction conditions: **3** (6.0 mg, 2.8 mol Pd% equiv; 4.5 mg, 2.1 mol Pd% equiv; 3.0 mg, 1.4 mol Pd% equiv), benzaldehyde (0.5 mmol), phenylacetylene (0.5 mmol) and pyrrolidine (0.75 mmol), solvent (2 mL), 300 W xenon with a power density of 2.5 W cm^−2^ (*λ* *>* 400 nm), in nitrogen, 10 h

^b^Yield was determined by the GC on HP-5 column, and ee was determined by HPLC with a Chiralcel OJ-H column (99: 1 = *n*-hexane: isopropanol, 1.0 mL min^−1^, 254 nm) (Supplementary Figs. 21 and 22). The comparison of **3** with reported asymmetric A^3^-coupling reaction was shown in Supplementary Table 5. **3** herein met excellent yield and stereoselectivity, mild reaction conditions and multiple reuse

The turnover number (TON) and turnover frequency (TOF) for the model reaction are 46.7 and 4.67 h^−1^, respectively. The hot leaching test confirmed **3** is a typical heterogeneous catalyst (Supplementary Figs. 24–29). Additionally, **3** can be reused and the yield was still up to 91% (92% ee) even after five catalytic cycles (Supplementary Fig. 25). Also, the crystallinity and structural integrity of the reused **3** were well maintained, which was confirmed by the PXRD patterns (Supplementary Fig. 29). No significant Pd species loss in **3** was observed after five catalytic cycles (Pd 20.1 wt%, Cu 4.4 wt % based on ICP-AES). Thus, **1** is an ideal scaffold to support Pd NP for this asymmetric A^3^-coupling reaction.

In addition, **3** could also be applicable to various substrates with either electron-donating or electron-withdrawing groups at different substituted positions with good-to-excellent yields (68–98%) and excellent ee values (90–98% ee, Table 4 (entries 1–26), Supplementary Figs. 30–31). As shown in Table 4, most of the substrates under the optimized conditions furnished good-to-excellent yields (68–98%, entries 1–26) with excellent ee values (90–98% ee, entries 1–26). Meanwhile, the larger-sized substrates such as 9-anthraldehyde and 2-fluorenecarboxaldehyde (Table 4, entries 27–28) were unfavorable for the reaction, and the corresponding products were obtained in lower yields (37–38%) with 87–89% ee, indicating that the asymmetric A^3^-coupling reactions herein should be an inner pore catalytic process. Notably, benzaldehyde or the *para*-substituted aromatic aldehydes gave the products with *S* configuration (Table 4, entries 1–19), while the *meta*-substituted aromatic aldehydes generated the products with *R* configuration (Table 4, entries 20–26). Although we currently distance ourselves from any type of explanation and say forthright that we do not know why the different position substituted aromatic aldehydes preferred the different chiral configurations, it is much possible because of the different templating effect toward the *para*- or *meta*-substrates caused by the rigid chiral host-framework. When the reactions were carried out at 0 °C, the same results were obtained.

**Table 4:** Scope of the **3**-catalyzed asymmetric A^3^-coupling reactions^a^


Entry	R_1_	R_2_	R_3_ R_4_	Yield (%)^b^	ee (%)^b^	TON
1	Ph−	Ph−	−(CH_2_)_4_−	98	96 (*S*)	46.7
2	Ph−	4-Me−Ph−	−(CH_2_)_4_−	92	96 (*S*)	43.8
3	Ph−	4-MeO−Ph−	−(CH_2_)_4_−	90	94 (*S*)	42.9
4	Ph−	4-NO_2_−Ph−	−(CH_2_)_4_−	96	98 (*S*)	45.7
5	Ph−	4-F−Ph−	−(CH_2_)_4_−	97	98 (*S*)	46.2
6	4-Me−Ph−	Ph−	−(CH_2_)_4_−	94	95 (*S*)	44.8
7	4-MeO−Ph−	Ph−	−(CH_2_)_4_−	95	98 (*S*)	45.2
8	4-NO_2_−Ph−	Ph−	−(CH_2_)_4_−	86	92 (*S*)	41.0
9	4-F−Ph−	Ph−	−(CH_2_)_4_−	88	95 (*S*)	41.9
10	4-Me−Ph−	4-NO_2_−Ph−	−(CH_2_)_4_−	95	98 (*S*)	45.2
11	4-MeO−Ph−	4-NO_2_−Ph−	−(CH_2_)_4_−	95	98 (*S*)	45.2
12	4-NO_2_−Ph−	4-NO_2_−Ph−	−(CH_2_)_4_−	83	95 (*S*)	39.5
13	4-F−Ph−	4-NO_2_−Ph−	−(CH_2_)_4_−	84	95 (*S*)	40.0
14	4-Me−Ph−	4−Me−Ph−	−(CH_2_)_4_−	86	93 (*S*)	41.0
15	4-MeO−Ph−	4-Me−Ph−	−(CH_2_)_4_−	84	91 (*S*)	40.0
16	4-NO_2_−Ph−	4-Me−Ph−	−(CH_2_)_4_−	91	94 (*S*)	43.3
17	4-F−Ph−	4-Me−Ph−	−(CH_2_)_4_−	92	95 (*S*)	43.8
18	Ph−	Ph−	−(CH_2_)_2_O(CH_2_)_2_−	68	90 (*S*)	32.4
19	Ph−	Ph−	−(CH_2_)_5_−	95	95 (*S*)	45.2
20	3-Me−Ph−	Ph−	−(CH_2_)_4_−	98	96 (*R*)	46.7
21	3-MeO−Ph−	Ph−	−(CH_2_)_4_−	98	94 (*R*)	46.7
22	3-NO_2_−Ph−	Ph−	−(CH_2_)_4_−	86	95 (*R*)	41.0
23	3-Me−Ph−	4-Me−Ph−	−(CH_2_)_4_−	87	91 (*R*)	41.4
24	3-Me−Ph−	4-NO_2_−Ph−	−(CH_2_)_4_−	95	95 (*R*)	45.2
25	3-NO_2_−Ph−	4-Me−Ph−	−(CH_2_)_4_−	94	93 (*R*)	44.8
26	3-NO_2_−Ph−	4-NO_2_−Ph−	−(CH_2_)_4_−	86	94 (*R*)	41.0
27	9-anthral	Ph−	−(CH_2_)_4_−	37	87 (*S*)	17.6
28	Fluorene-2-carboxal	Ph−	−(CH_2_)_4_−	38	89 (*S*)	18.1

^a^Reaction conditions: N_2_, **3** (4.5 mg, 2.1 mol% Pd), aromatic aldehyde (0.5 mmol), aromatic alkyne (0.5 mmol) and secondary amine (0.75 mmol), *p*-dioxane (2 mL), r.t., 300 W xenon with a power density of 2.5 W cm^−2^ (*λ* *>* 400 nm)

^b^Yield and ee are determined by GC and chiral HPLC, respectively (Supplementary Figs. 30 and 31)

### Photothermal behavior of 1–3

As shown above, the **2**-catalyzed Henry and **3**-catalyzed A^3^-coupling reactions herein are typical thermally-driven instead of a photoinduced reactions, and the maximum yield for either one-pot Henry reaction or A^3^-coupling reaction was obtained at elevated temperatures by photothermal conversion. To further evaluate the photothermal conversion of **1**–**3**, the visible light induced temperature increase (∆T) of above reaction systems (i.e., PhMe/EtOH (1:1) and *p*-dioxane) in the presence of catalytic amounts of **1–3** was tested based on their absorption spectra (Supplementary Fig. 32).

**Fig. 2:**
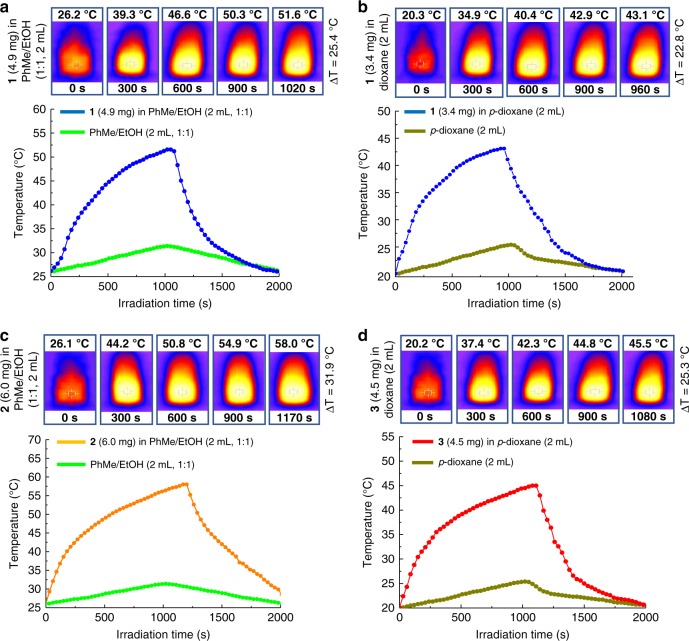
Photothermal examination of **1–3**. **a**, **b** Photothermal behavior of **1** in PhMe/EtOH (1:1, 2 mL) and *p*-dioxane (2 mL), respectively. **c** Photothermal effect of **2** in PhMe/EtOH (1:1, 2 mL). **d** Photothermal effect of **3** in *p*-dioxane (2 mL). Visible light source: 300 W xenon lamp, *λ* > 400 nm with the intensity at 2.5 W cm^−2^. 4.9 and 3.4 mg of **1** are equivalent to the CCOF content in 6.0 mg of **2** and 4.5 mg of **3**, respectively

As shown in Fig. 2a, b, when the PhMe/EtOH (1:1, 2 mL)-**1** (4.9 mg) system was irradiated with visible light for 17 min., a significant temperature increase (∆T) of 25.4 °C was detected. For *p*-dioxane (2 mL)-**1** (3.4 mg) system, the measured ∆T is 22.8 °C at 16 min. For **2**, a 31.9 °C temperature increase was observed in PhMe/EtOH (1:1, 2 mL)−**2** (6.0 mg) system upon irradiation for 19.5 min (Fig. 2c). Meanwhile, ∆T of 25.3 °C was presented when *p*-dioxane (2 mL)-**3** (4.5 mg) system was illuminated for 18 min (Fig. 2d). As shown in Fig. 1a, the 2D porphyrin-derived CCOF stacking nature, together with the central Cu(II) ion, induced the formation of porphyrin-aggregates in **1**, which enabled the obtained CCOF microparticles with effective light-to-heat conversion efficiency^41^. Thus, the porphyrin-based CCOF **1** herein is responsible for not only the chiral templating and partial catalytic functionality, but also the photothermal conversion. The slight difference in ∆T by **1**–**3** should be caused by different type and amount of the embedded M NP with different amount of **1** in different media.

This interesting phenomenon inspired us to further explore the chiral templating ability of **1** at even higher temperature, which is very important factor to balance the yield and enantioselectivity in asymmetric catalysis. The one-pot Henry and A^3^-coupling model reactions were therefore conducted within a temperature range of 60–100 °C under the optimized conditions. The obtained results were summarized in Table 5.

**Table 5:** **2**- and **3**-catalyzed asymmetric reactions at different temperatures^a^


T(°C)	60	70	80	90	100
Yield (%)^b^	97	97	98	98	99
ee (%)^*b*^	96	97	95	93	88

T(°C)	60	70	80	90	100
Yield (%)^b^	97	97	98	98	99
ee (%)^b^	97	96	96	92	86

^a^Reactions are carried out under the optimized conditions except in dark by heating

^b^Yield and ee are determined by GC and chiral HPLC, respectively (Supplementary Fig. 33)

We are very pleased to find that the chiral confined effect of **1** could be well maintained even at a higher temperature. As shown in Table 5, **2**-catalyzed model one-pot Henry reaction of benzyl alcohol with nitromethane gave the target chiral *β*-nitro alcohol in 97–98% yields but with an excellent 93–96% ee within 60–90 °C. At 100 °C, the reaction provided 99% yield but still with a significant stereoselectivity (88% ee). The similar result was observed in the **3**-catalyzed model A^3^-coupling reaction of benzaldehyde-pyrrolidine-phenylacetylene. The desired pyrrolidine-derived propargylamine was generated in 97–98% yields with excellent 92–97% ee in a temperature range of 60–90 °C. Again, a good enough enantiopurity (86% ee) was obtained even at 100 °C but with an excellent 99% yield. Thus, the M@CCOF-CuTPP herein could well tolerate high-temperature condition and broke the bottleneck of high enantiopurity at the cost of yield in the thermally-driven asymmetric catalysis, especially for those low-yield asymmetric catalysis caused by the low-temperature condition. So far, we are not aware of any reported asymmetric catalysts possessing such a feature.

## Discussion

As mentioned above, M@CCOF-CuTPP of **2** (M=Au) and **3** (M=Pd) are the multifunctional catalytic materials and they can highly promote thermally-driven asymmetric catalysis at the elevated temperature by photothermal conversion. First, CCOF-CuTPP (**1**) is a rigid homochiral host-framework^42–45^, and it can be a powerful chiral temple to steer the organic substrates in the specific spatial orientation within the CCOF confined space even at high temperature, consequently, provide the desired products with high enantiopurity; second, **1** contains porphyrin moiety which is a widely recognized PTCM that can readily transfer light into thermal energy^46^. Therefore, the obtained porphyrin-involved CCOF herein can supply the reaction needed thermal energy upon visible light irradiation; and third, **1** is highly porous and heteroatom-rich COF host which is qualified for the M NP loading and stabilization^47^.

Notably, the 2D porphyrin-based CCOF herein involves the connection of building blocks through a carbon–nitrogen (C–N) single bond linkage under the Pd-catalyzed reaction. This result implies that C–N bonding enables the system to proceed a thermodynamically controlled process, in which the crystallinity of an in-plane structure is handled via continued 2D chain growth and self-healing processes. It is different from the most commonly reported C=N driven COFs, the C–N single bond connected COF is chemically stable toward the reducing agent such as NaBH_4_, which is widely used to prepare the M NP loaded catalytic materials.

In summary, the concept of CCOF-based multifunctional integration leads the enantioselective synthesis to be economical, safe, eco-friendly, energy and source saving, more importantly, broke through the bottleneck of high enantiopurity at the cost of yield in the thermal-driven asymmetric catalysis. We think this approach to be general, effective and viable for the fabrication of many more practical multifunctional composite catalytic materials for a variety of asymmetric thermally-driven chemical transformations with both high yield and stereoselectivity.

## Methods

### Synthesis of CCOF-CuTPP (1)

Under nitrogen, a mixture of *S*-(+)-2-methylpiperazine (0.4 mmol, 40 mg), anhydrous K_2_CO_3_ (1.2 mmol, 166 mg) and Pd[P(Ph)_3_]_4_ (0.03 mmol, 33 mg) in anhydrous *p*-dioxane (50 mL) was stirred at room temperature. Then, the mixture was heated to 90 °C, and the TBrPP (0.2 mmol, 186 mg) in anhydrous *p*-dioxane (6 mL) was added dropwise. After refluxed for 3 days at 90 °C, the reaction system was allowed to stand at ambient temperature for two days. The purple-black crystalline product of **1** was collected by filtration and completely washed with HCl (1.0 M, 10 mL), water, dichloromethane, and ethanol to remove the unreacted starting precursors. Yield, 63%.

### Synthesis of Au@CCOF-CuTPP (2)

**1** (5 mg) was added to a CH_3_CN (1.5 mL) solution of KAuCl_4_ (5.0 mg, 0.09 mmol). The mixture was stirred for 3 h at room temperature. The resulting solid was isolated by centrifugation and washed with CH_3_CN and EtOH, respectively. The obtained solid was mixed with ascorbic acid (2.5 mg, 0.05 mmol) in water (1.5 mL) and stirred at room temperature for an additional 1 h to afford **2**. The obtained crystalline solids were completely washed with H_2_O, CH_3_CN and EtOH and dried in air. Yield, 98%.

### Synthesis of Pd@CCOF-CuTPP (3)

**1** (5 mg) was added to a CH_3_CN (1.5 mL) solution of palladium nitrate (5.0 mg, 0.09 mmol). The mixture was stirred for 3 h at room temperature. The resulting solids were isolated by centrifugation and washed with CH_3_CN and EtOH. The obtained solids were mixed with NaBH_4_ (2 mg, 0.05 mmol) in water (1.5 mL) and stirred at room temperature for additional 1 h to afford **3**. The obtained crystalline solids were completely washed with H_2_O, CH_3_CN and EtOH and dried in air. Yield, 97%.

### Model one-pot asymmetric Henry reaction catalyzed by 2

A mixture of benzyl alcohol (0.5 mmol), nitromethane (1.50 mmol), K_2_CO_3_ (1.50 mmol) and **2** (6.0 mg, 1 mol% Au equiv) in PhMe/EtOH (1:1, 2 mL) was stirred under 300 W xenon lamp (*λ* > 400 nm with the intensity at 2.5 W cm^−2^, 30 cm away from the reaction vessel) irradiation for 10 h in air to afford the corresponding adducts. The yield was determined by the GC measurement on HP-5 column, and ee was determined by HPLC with a Chiralcel OD-H column (90: 10 = *n*-hexane: isopropanol, 1.0 mL min^−1^, 230 nm), respectively.

### Model one-pot asymmetric A^3^-coupling reaction catalyzed by 3

Under nitrogen, a mixture of benzaldehyde (0.5 mmol), phenylacetylene (0.5 mmol), pyrrolidine (0.75 mmol) and **3** (4.5 mg, 2.1 mol% Pd equiv) in *p*-dioxane (2 mL) was stirred at room temperature under 300 W xenon lamp (*λ* > 400 nm with the intensity at 2.5 W cm^−2^, 30 cm away from the reaction vessel) irradiation for 10 h to afford the corresponding product. Yield was determined by the GC measurement on HP-5 column, and ee was determined by HPLC with a Chiralcel OJ-H column (99: 1 = *n*-hexane: isopropanol, 1.0 mL min^−1^, 254 nm), respectively.

## Supplementary information

Supporting Information

Source Data

## Data Availability

The source data supporting the findings of this study are available within the article, as well as the Supplementary Information file, or available from the corresponding authors on reasonable request.
