# Supplementary material for: Photothermal conversion triggered thermal asymmetric catalysis within metal nanoparticles loaded homochiral covalent organic framework
Source: Nat Commun. 2019 Jul 29;10:3368. doi: 10.1038/s41467-019-11355-x (PMC6662712; doi:10.1038/s41467-019-11355-x)
Supplement: Supplementary file 1 — Supporting Information [file 41467_2019_11355_MOESM1_ESM.pdf]

Supporting Information for

Photothermal conversion triggered thermal asymmetric catalysis within metal nanoparticles loaded homochiral covalent organic framework

Ma et al.

Supplementary Methods

Materials and instruments. The reagents and solvents employed were commercially available and used without further purification. TBrPP (tetra(4-bromophenyl)porphyrin) was prepared according to reported method.[1] The powder diffractometer (XRD) patterns were collected by a D8 ADVANCEX-ray with Cu Kα radiation (λ = 1.5405 Å). The total surface areas of the catalysts were measured by the BET (Brunauer–Emmer–Teller) method using N_2 adsorption at 77 K, this was done by the Micromeritics ASAP 2000 sorption/desorption analyzer. Inductively coupled plasma (ICP) measurement was conducted on an IRIS Intrepid (II) XSP and NU AttoM spectrometer. HRTEM (High resolution transmission electron microscopy) analysis was performed on a JEOL 2100 Electron Microscope at an operating voltage of 200 kV. Scanning electron microscopy (SEM) images were taken on a SUB010 scanning electron microscope with acceleration voltage of 20 kV. Elemental analyses for C, H and N were obtained on a Perkin-Elmer analyzer model 240. Infrared (IR) samples were prepared as KBr pellets, and spectra were obtained in the 400-4000cm^{-1} range using a Perkin-Elmer 1600 FTIR spectrometer. ^{13}C NMR spectra were recorded on a MERCURY plus 400 spectrometer operating at resonance frequencies of 400 MHz. Thermogravimetric analyses (TGA) were carried out under flowing nitrogen at a heating rate of 10 °C·min^{-1} on a TA Instrument Q5 analyzer. The solid-state CD spectra were recorded on a J-815 spectropolarimeter (Jasco, Japan). XPS spectra were obtained from PHI Versaprobe II. UV-vis spectrum was recorded on a Cary 5000 UV-vis spectrophotometer (Varian, USA). Gas chromatography (GC) analysis was performed on an Agilent 7890B GC. Enantiomer ratios were determined by chiral HPLC analysis using a Shimadzu LC-10AT VP series and a Shimadzu LC-10A VP UV-vis. Photocatalytic performances were evaluated by xenon lamp (300W with the intensities 2.5W cm^{-2}).

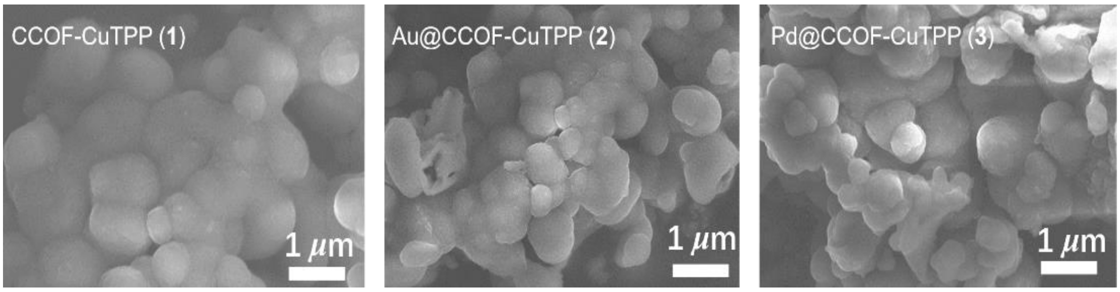

Supplementary Figure 1. SEM images of **1 - 3**.

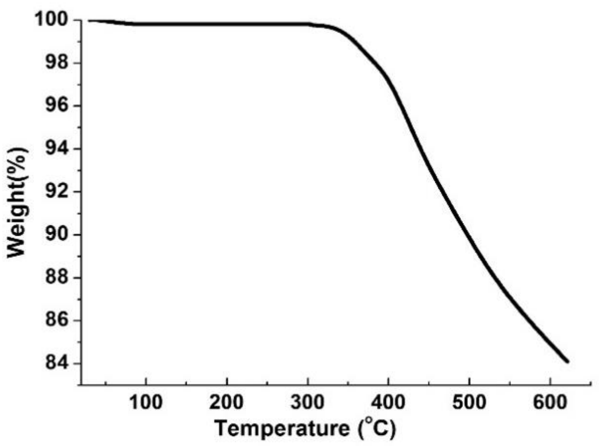

Supplementary Figure 2. TGA trace for the activated sample of **1**. No weight loss of **1** was observed up to ca. 350 °C.

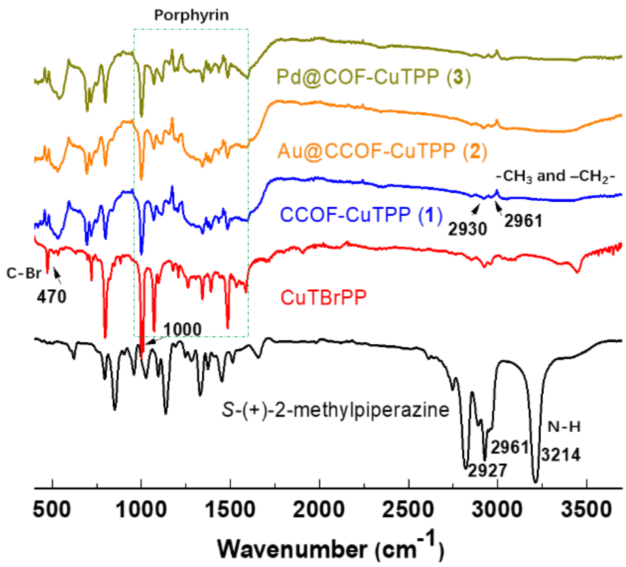

Supplementary Figure 3. IR spectra of CuTBPP, *S*-(+)-methylpiperazine, and **1** - **3**. The IR spectrum of **1** showed that the characteristic N-H stretching vibration at 3214 cm^{-1} of *S*-MP and C-Br stretching vibration of TBrPP at 470 cm^{-1} disappeared after the reaction. Meanwhile the C-H stretching vibrations (2961 and 2927 cm^{-1} for methylpiperazinyl C-H bonds) and characteristic porphyrin stretching vibrations (1340, 1390, 1490 and 1590 cm^{-1} for porphyrin C=N bonds and 1000 cm^{-1} for porphyrin skeleton vibration) indicated that both the methylpiperazinyl and porphyrin units existed in **1**. In addition, **1-3** were characterized by elemental analysis or ICP. Elemental Analysis (%) calcd for $C_{54}H_{48}CuN_8$ (**1**): C, 74.33; N, 12.84; H, 5.54. Found (%): C, 74.04; N, 12.65; H, 6.02. ICP measurement indicated that the amount of Cu in **1** is 6.9 wt % (calcd. 7.2 wt %). The Au amount in **2** based on ICP is 17.9 wt %. The Pd amount in **3** based on ICP is 24.8 wt %.

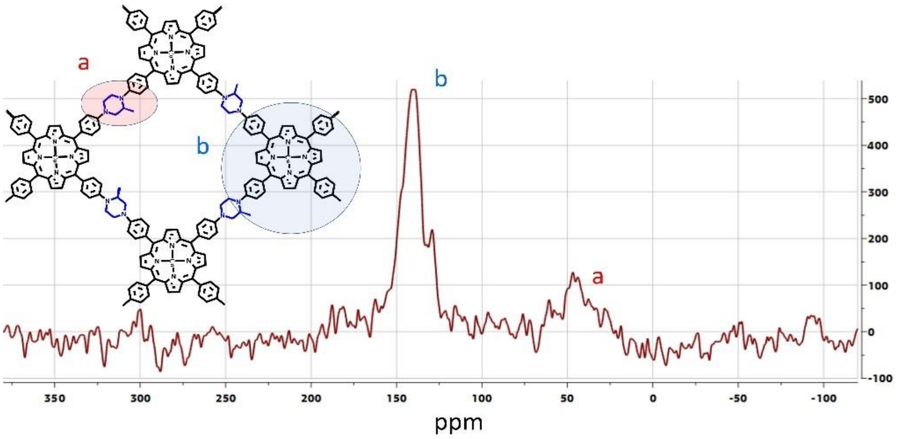

Supplementary Figure 4. Solid-state ^{13}C NMR of **1**. The existence of both methylpiperazinyl and porphyrin units in **1** was evidenced by the characteristic resonances at 50 and 148 ppm, respectively.

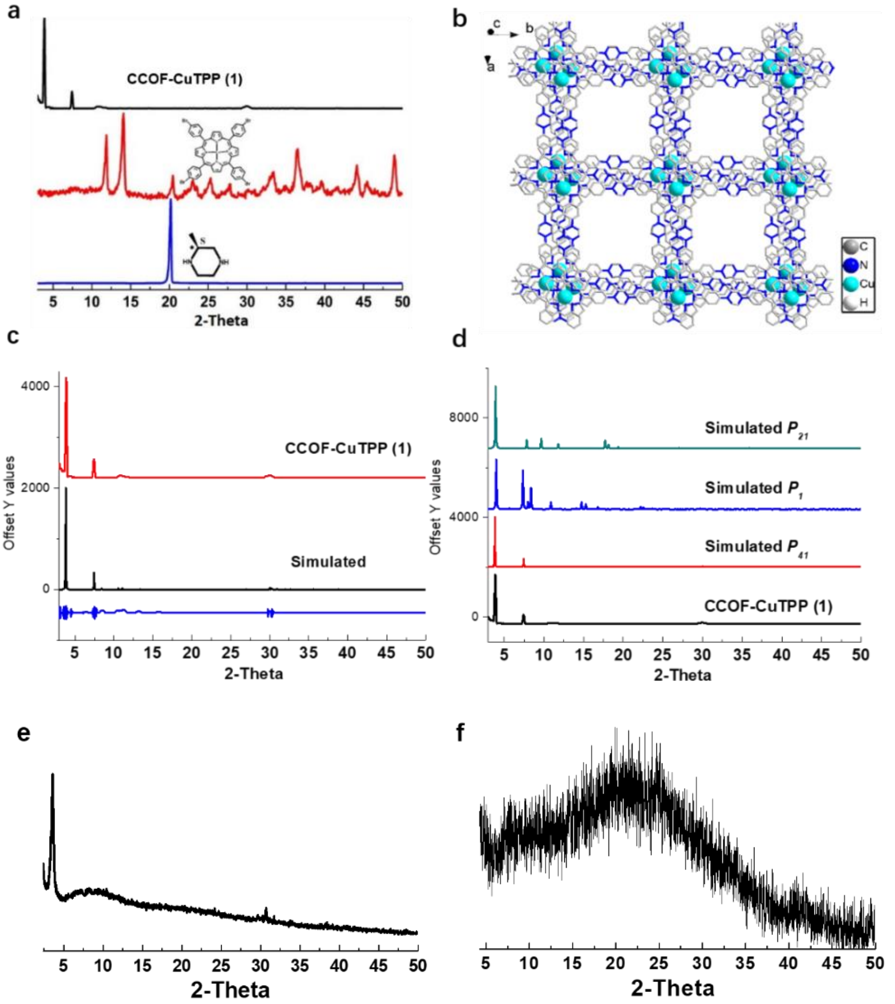

Supplementary Figure 5. **a**, PXRD patterns of **1**, *S*-(+)-2-methylpiperazine and Cu(II)-TBrPP. **b**, View of the ABCD-layered **1** (down the crystallographic *c* axis). Hydrogen atoms are omitted for clarity. **c**, The difference plot between measured and Rietveld-refined PXRD pattern of **1**. **d**, Simulated PXRD patterns of P_{21}, P_1 and P_{41} modes and the measured PXRD pattern of **1**. Compared to P_{21} and P_1 modes, the P_{41} mode is more energetic preferential. **e**, The measured PXRD pattern of CCOF TPP without Cu^{2+}. **f**, The PXRD of amorphous CCOF (**1**).

Supplementary Table 1. Simulated structure of 1.

Structural modeling of **1** was generated using the Materials Studio (ver. 2018) suite of programs. Molecular geometry optimization was performed with MS DMol3 module. The initial lattice was created by starting with the space group P_{41}. The *a* and *b* lattice parameters (initially 22.073 Å) were estimated according to the center to center distance between the vertices of the COF. The constructed model was geometry optimized using the Forcite module (Universal force fields, Ewald summations). Then the calculated PXRD pattern was generated with the Reflex Plus module. Finally, Pawley refinement was applied for profile fitting, producing the refined PXRD profile with the lattice parameters of $a = b = 22.073$ (± 0.002) Å and $c = 12.000$ (± 0.002) Å. *R*wp and *R*p values converged to 3.72% and 2.89%, respectively (Supplementary Table 1).

Reflex Module in Material Studio using data from 2θ =3.0-50° (Supplementary Fig. 5). Backgrounds were first refined applying a 2nd order Chebyschev Polynomial. The profile was calculated starting with the unit cell parameters from the crystal models and the space group P_{41}. The integrated intensities (Iobs) were extracted by a full pattern decomposition using a Thomson-Cox-Hastingpseudo Voigt peak profile, followed by refinement of peak asymmetry using Finger et al. asymmetry function (2 parameters). Unit cells and zero-shift were then refined with peak asymmetry. Once this was achieved, the background was refined with 20th-order polynomial. Refinement of unit cell parameters, zero shift, peak asymmetry, crystallite size and strain were used for the final profile.

Based on the measured and the simulated PXRD patterns (Supplementary Fig. 5), it is not possible to assign the CCOF-CuTPP (**1**) to P_1 or P_{21} mode (Supplementary Table 2 and Table 3). The preferable structure of CCOF-CuTPP (**1**) is the P_{41} mode.

The total energies were calculated from fully optimized structures (Materials Studio Forcite molecular geometry optimization module, universal force fields using charges Qeq_neutral 1.0, Ewald summations method). The total crystal stacking energy per layer has been obtained from the expression: Etot (crystal stacking energy per layer) = [N×Etot(monolayer)-Etot(bulk)]/N, where Etot (bulk) and Etot (monolayer) are the total energies of the fully optimized crystal bulks and isolated monolayers, respectively, and N is the number of layers considered in the crystal bulk calculations. Monolayer energy is 394.05 kcal/mol, P_{41} space group (**1**) with ABCD stacking mode possessed the highest total crystal stacking energy per layer (338.57 kcal/mol) among other types of possible simulated space groups (P_{21} 251.30 kcal/mol, P_1 49.31 kcal/mol) with different stacking modes.

Supplementary Table 1. The structure model of CCOF-CuTPP (**1**) with $P4_1$ mode.

CCOF-CuTPP (**1**) Space group: $P4_1$<br>a = 22.073 Å, b = 22.073 Å, c = 12.000 Å<br>α = 90.0°, β = 90.0°, γ = 90.0°			
Atom	x	y	z
C1	0.36981	-1.85856	0.00699
C2	0.33488	-1.80544	0.00645
C3	0.27144	-1.80529	0.00623
C4	0.23640	-1.85839	0.00662
C5	0.27122	-1.91154	0.00750
C6	0.33464	-1.91164	0.00779
C7	-0.11873	-1.69060	-0.00431
C8	-0.16726	-1.73905	0.00144
C9	-0.13368	-1.79983	0.00336
N10	-0.06509	-1.79275	-0.00069
C11	-0.05793	-1.72420	-0.00559
C12	0.00040	-1.68945	-0.00835
C13	-0.16838	-1.85777	0.00597
C14	0.11859	-2.02528	0.00042
C15	0.16712	-1.97675	0.00298
C16	0.13364	-1.91594	0.00329
N17	0.06504	-1.92306	0.00125
C18	0.05784	-1.99168	-0.00016
C19	0.16847	-1.85803	0.00388
C20	-0.00021	-2.02631	0.00053
C21	-0.16736	-1.97647	0.00662
C22	-0.11894	-2.02509	0.00394
C23	-0.05808	-1.99157	0.00189
N24	-0.06516	-1.92295	0.00275
C25	-0.13372	-1.91570	0.00560
C26	0.16744	-1.73916	-0.00071
C27	0.11893	-1.69060	-0.00626
C28	0.05807	-1.72411	-0.00623
N29	0.06524	-1.79274	-0.00122
C30	0.13388	-1.79997	0.00183
Cu31	0.00004	-1.85788	0.00051
C32	-0.23635	-1.85767	0.00696
C33	-0.27128	-1.80454	0.00643
C34	-0.33472	-1.80439	0.00620
C35	-0.36976	-1.85750	0.00659
C36	-0.33494	-1.91065	0.00747
C37	-0.27151	-1.91074	0.00777

N38	0.43407	-1.85954	-0.03098
C39	0.46804	-1.91871	-0.04082
C40	0.52765	-1.91577	0.02563
N41	0.56285	-1.85994	-0.00743
C42	0.52779	-1.80438	0.02776
C43	0.46799	-1.80024	-0.03794
C44	-0.00007	-2.09413	0.00848
C45	-0.05319	-2.12905	0.00852
C46	-0.05334	-2.19249	0.00870
C47	-0.00023	-2.22753	0.00898
C48	0.05292	-2.19271	0.00930
C49	0.05301	-2.12928	0.00918
C50	0.00018	-1.48844	0.00460
C51	-0.05303	-1.52353	0.00463
C52	-0.05296	-1.58697	0.00482
C53	0.00026	-1.62182	0.00509
C54	0.05330	-1.58682	0.00541
C55	0.05317	-1.52339	0.00529
N56	-0.00073	-1.42434	-0.03379
C57	0.05835	-1.39029	-0.04423
C58	0.05544	-1.33056	0.02185
N59	-0.00052	-1.29552	-0.01108
C60	-0.05595	-1.33060	0.02470
C61	-0.06010	-1.39054	-0.04059
C62	-0.05375	-1.19318	0.00229
C63	-0.00070	-1.22832	0.00257
C64	0.05251	-1.19359	0.00288
C65	0.56561	0.25270	0.00073
C66	0.88708	0.70735	0.02185
H67	0.56608	0.26037	-0.09321
H68	0.61398	0.24612	0.03175
H69	0.54466	0.29372	0.04372
H70	0.84705	0.67899	0.05157
H71	0.87840	0.72342	-0.06701
H72	0.89281	0.74827	0.07885
H73	0.35806	0.24071	0.00618
H74	0.24572	0.23950	0.00571
H75	0.24795	0.04235	0.00801
H76	0.36040	0.04360	0.00873
H77	0.21700	0.00988	0.00421
H78	0.21733	0.27419	0.00072
H79	0.13235	0.35924	-0.00941

H80	0.47861	0.07208	-0.13225
H81	0.43890	0.04296	-0.00649
H82	0.55589	0.04216	0.00722
H83	0.51709	0.08621	0.11855
H84	0.51793	0.19362	0.12094
H85	0.47831	0.21045	-0.12892
H86	0.43864	0.23732	-0.00140
H87	0.09949	0.39008	0.00578
H88	0.09784	0.50252	0.00578
H89	0.06737	0.62012	-0.13579
H90	0.09681	0.58070	-0.00995
H91	0.09742	0.69772	0.00299
H92	0.05365	0.65905	0.11485
H93	0.09757	0.78159	0.01133
H94	0.86792	0.35927	-0.00671
H95	0.78288	0.27437	0.00340
H96	0.78275	0.01025	0.00882
H97	0.75191	0.24161	0.00615
H98	0.63956	0.24040	0.00568
H99	0.64179	0.04324	0.00799
H100	0.75424	0.04449	0.00870
H101	0.90078	0.49959	0.00450
H102	0.90234	0.38716	0.00475
H103	0.95160	0.65652	0.11564
H104	0.92902	0.61959	-0.13157
H105	0.90245	0.58011	-0.00362
H106	0.13197	0.92484	-0.00064
H107	0.86760	0.92505	0.00381

Supplementary Table 2. The structure model of CCOF-CuTPP (**1**) with P_1 mode.

CCOF-CuTPP (**1**) Space group: P_1<br>a = 22.073 Å, b = 22.073 Å, c = 12.000 Å<br>α = 90.0°, β = 90.0°, γ = 90.0°			
C1	0.36981	-1.85856	0.00699
C2	0.33488	-1.80544	0.00645
C3	0.27144	-1.80529	0.00623
C4	0.23640	-1.85839	0.00662
C5	0.27122	-1.91154	0.00750
C6	0.33464	-1.91164	0.00779
C7	-0.11873	-1.69060	-0.00431
C8	-0.16726	-1.73905	0.00144
C9	-0.13368	-1.79983	0.00336

N10	-0.06509	-1.79275	-0.00069
C11	-0.05793	-1.72420	-0.00559
C12	0.00040	-1.68945	-0.00835
C13	-0.16838	-1.85777	0.00597
C14	0.11859	-2.02528	0.00042
C15	0.16712	-1.97675	0.00298
C16	0.13364	-1.91594	0.00329
N17	0.06504	-1.92306	0.00125
C18	0.05784	-1.99168	-0.00016
C19	0.16847	-1.85803	0.00388
C20	-0.00021	-2.02631	0.00053
C21	-0.16736	-1.97647	0.00662
C22	-0.11894	-2.02509	0.00394
C23	-0.05808	-1.99157	0.00189
N24	-0.06516	-1.92295	0.00275
C25	-0.13372	-1.91570	0.00560
C26	0.16744	-1.73916	-0.00071
C27	0.11893	-1.69060	-0.00626
C28	0.05807	-1.72411	-0.00623
N29	0.06524	-1.79274	-0.00122
C30	0.13388	-1.79997	0.00183
Cu31	0.00004	-1.85788	0.00051
C32	-0.23635	-1.85767	0.00696
C33	-0.27128	-1.80454	0.00643
C34	-0.33472	-1.80439	0.00620
C35	-0.36976	-1.85750	0.00659
C36	-0.33494	-1.91065	0.00747
C37	-0.27151	-1.91074	0.00777
N38	0.43407	-1.85954	-0.03098
C39	0.46804	-1.91871	-0.04082
C40	0.52765	-1.91577	0.02563
N41	0.56285	-1.85994	-0.00743
C42	0.52779	-1.80438	0.02776
C43	0.46799	-1.80024	-0.03794
C44	-0.00007	-2.09413	0.00848
C45	-0.05319	-2.12905	0.00852
C46	-0.05334	-2.19249	0.00870
C47	-0.00023	-2.22753	0.00898
C48	0.05292	-2.19271	0.00930
C49	0.05301	-2.12928	0.00918
C50	0.00018	-1.48844	0.00460
C51	-0.05303	-1.52353	0.00463

C52	-0.05296	-1.58697	0.00482
C53	0.00026	-1.62182	0.00509
C54	0.05330	-1.58682	0.00541
C55	0.05317	-1.52339	0.00529
N56	-0.00073	-1.42434	-0.03379
C57	0.05835	-1.39029	-0.04423
C58	0.05544	-1.33056	0.02185
N59	-0.00052	-1.29552	-0.01108
C60	-0.05595	-1.33060	0.02470
C61	-0.06010	-1.39054	-0.04059
C62	-0.05375	-1.19318	0.00229
C63	-0.00070	-1.22832	0.00257
C64	0.05251	-1.19359	0.00288
C65	0.56561	0.25270	0.00073
C66	0.88708	0.70735	0.02185
H67	0.35806	-1.75929	0.00618
H68	0.24572	-1.76050	0.00571
H69	0.24795	-1.95765	0.00801
H70	0.36040	-1.95640	0.00873
H71	-0.13208	-1.64073	-0.00671
H72	-0.21712	-1.72563	0.00340
H73	0.13197	-2.07516	-0.00064
H74	0.21700	-1.99012	0.00421
H75	-0.21725	-1.98975	0.00882
H76	-0.13240	-2.07495	0.00381
H77	0.21733	-1.72581	0.00072
H78	0.13235	-1.64076	-0.00941
H79	-0.24809	-1.75839	0.00615
H80	-0.36044	-1.75960	0.00568
H81	-0.35821	-1.95676	0.00799
H82	-0.24576	-1.95551	0.00870
H83	0.47861	-1.92792	-0.13225
H84	0.43890	-1.95704	-0.00649
H85	0.55589	-1.95784	0.00722
H86	0.51709	-1.91379	0.11855
H87	0.51793	-1.80638	0.12094
H88	0.47831	-1.78955	-0.12892
H89	0.43864	-1.76268	-0.00140
H90	-0.09922	-1.50041	0.00450
H91	-0.09766	-1.61284	0.00475
H92	0.09949	-1.60992	0.00578
H93	0.09784	-1.49748	0.00578

H94	0.06737	-1.37988	-0.13579
H95	0.09681	-1.41930	-0.00995
H96	0.09742	-1.30228	0.00299
H97	0.05365	-1.34095	0.11485
H98	-0.04840	-1.34348	0.11564
H99	-0.07098	-1.38041	-0.13157
H100	-0.09755	-1.41989	-0.00362
H101	0.09757	-1.21841	0.01133
H102	0.56608	0.26037	-0.09321
H103	0.61398	0.24612	0.03175
H104	0.54466	0.29372	0.04372
H105	0.84705	0.67899	0.05157
H106	0.87840	0.72342	-0.06701
H107	0.89281	0.74827	0.07885

Supplementary Table 3. The structure model of CCOF-CuTPP (**1**) with P_{21} mode.

CCOF-CuTPP (**1**) Space group: P_{21}<br>a = 22.5300 Å, b = 10.0000 Å, c = 22.5300 Å<br>α = 90.0°, β = 90.0°, γ = 90.0°			
N1	-1.26833	-2.97246	0.49756
C2	-1.29862	-3.02948	0.55163
C3	-1.36375	-2.98280	0.55329
N4	-1.39435	-3.03346	0.49812
C5	-1.36533	-2.97252	0.44419
C6	-1.30007	-3.01807	0.44215
C7	-1.26568	-2.98225	0.60767
H8	-1.29725	-3.14333	0.54940
C9	-1.49518	-3.00148	0.43978
C10	-1.46083	-3.01172	0.49870
C11	-1.49465	-3.00144	0.55798
C12	-0.99545	-2.99994	0.38369
C13	-0.94779	-2.99993	0.33626
C14	-0.88829	-2.99994	0.36928
N15	-0.89543	-2.99991	0.43648
C16	-0.96269	-2.99992	0.44332
C17	-0.99687	-2.99989	0.49997
C18	-0.83149	-3.00013	0.33536
C19	-0.77469	-3.00032	0.36940
C20	-0.83168	-3.00001	0.26684
C21	-0.96300	-2.99979	0.55684
C22	-1.06542	-2.99995	0.49963
C23	-0.71510	-3.00031	0.33656

C24	-0.66755	-2.99942	0.38416
C25	-0.70041	-2.99889	0.44378
N26	-0.76771	-2.99975	0.43668
C27	-0.66637	-2.99749	0.50066
C28	-0.70062	-2.99874	0.55743
C29	-0.66798	-2.99933	0.61714
C30	-0.71571	-3.00032	0.66459
C31	-0.77523	-3.00042	0.63160
N32	-0.76797	-2.99966	0.56433
N33	-0.89574	-2.99993	0.56403
C34	-0.88890	-3.00025	0.63130
C35	-0.94860	-3.00004	0.66400
C36	-0.99605	-2.99979	0.61632
C37	-0.83220	-3.00092	0.66551
C38	-0.83266	-3.00192	0.73413
C39	-0.59765	-2.99581	0.50064
C40	-1.10015	-2.99995	0.55854
C41	-1.16852	-2.99997	0.55809
C42	-1.20234	-2.99998	0.49871
C43	-1.16774	-2.99997	0.43978
C44	-1.09939	-2.99996	0.44028
C45	-0.77386	-3.00136	0.76906
C46	-0.77451	-3.00030	0.83748
C47	-0.83398	-2.99886	0.87121
C48	-0.89278	-3.00287	0.83639
C49	-0.89209	-3.00374	0.76800
C50	-0.56335	-2.99510	0.44144
C51	-0.56290	-2.99496	0.55963
C52	-0.89099	-2.99996	0.23282
C53	-0.89140	-3.00005	0.16448
C54	-0.83242	-3.00007	0.12998
C55	-0.77307	-3.00001	0.16387
C56	-0.77271	-2.99996	0.23223
Cu57	-0.83169	-2.99988	0.50039
H58	-0.73824	-3.00591	0.05988
N59	-0.83337	-2.97266	0.06393
C60	-0.88753	-3.03045	0.03416
C61	-0.89184	-2.98340	-0.03077
N62	-0.83321	-2.98908	-0.06334
C63	-0.77534	-2.97983	-0.02966
C64	-0.78002	-3.03362	0.03413
H65	-1.36552	-2.86890	0.55434

H66	-1.38635	-3.02491	0.59452
H67	-1.36739	-2.85883	0.44730
H68	-1.38948	-3.00735	0.40250
H69	-1.27750	-2.97286	0.40155
H70	-1.29814	-3.13179	0.43925
H71	-1.22937	-2.90848	0.59464
H72	-1.24490	-3.07203	0.63092
H73	-1.29836	-2.93124	0.63897
H74	-1.47006	-2.99875	0.39587
H75	-1.46912	-2.99850	0.60164
H76	-1.04429	-2.99997	0.37047
H77	-0.96077	-2.99992	0.28735
H78	-0.71409	-3.00091	0.28597
H79	-0.61866	-2.99927	0.37110
H80	-0.61739	-2.99899	0.61832
H81	-0.70276	-3.00081	0.71350
H82	-0.94973	-3.00010	0.71459
H83	-1.04495	-2.99964	0.62930
H84	-1.07525	-2.99992	0.60259
H85	-1.19410	-2.99997	0.60175
H86	-1.19272	-2.99998	0.39578
H87	-1.07377	-2.99995	0.39665
H88	-0.72973	-3.00172	0.74431
H89	-0.73093	-3.00058	0.86319
H90	-0.93686	-3.00521	0.86121
H91	-0.93565	-3.00572	0.74226
H92	-0.58854	-2.98975	0.39762
H93	-0.58705	-2.98964	0.60403
H94	-0.93474	-2.99986	0.25824
H95	-0.93537	-3.00009	0.13944
H96	-0.72937	-3.00001	0.13837
H97	-0.72875	-2.99989	0.25730
H98	-0.76074	-2.87074	-0.02801
H99	-0.73990	-3.04062	-0.05367
C100	0.94553	0.46902	0.92966
H101	-0.87629	-3.14117	0.02986
H102	-0.90890	-2.87609	-0.03151
H103	-0.92449	-3.04972	-0.05582
H104	-0.78496	-3.14704	0.03298
H105	0.93611	0.50519	0.88261
H106	0.96419	0.36313	0.92803
H107	0.97920	0.53835	0.95156

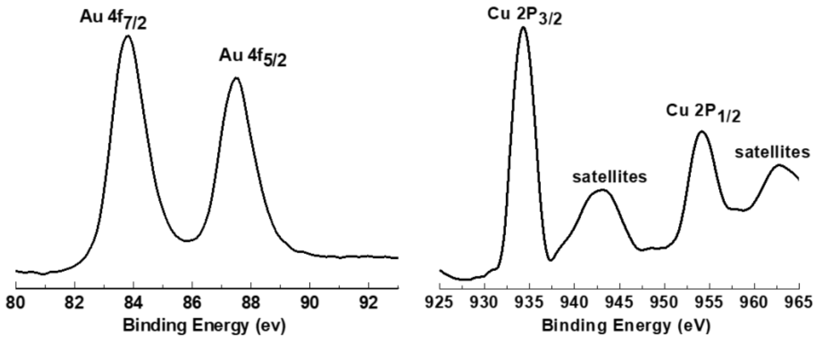

Supplementary Figure 6. XPS spectra of Au(0) (left) and Cu(II) (right) species in **2**. The observation of Au $4f_{7/2}$ and $4f_{5/2}$ peaks at 84.0 and 87.5 eV demonstrated the existence of Au(0) in **2**. In addition, the two peaks at 934.5 and 954.8 eV in XPS spectrum were assigned to Cu ($2p_{3/2}$) and Cu($2p_{1/2}$), indicating that the oxidation state of Cu species in **2** is still bivalent and no valence variation occurred during the Au(III)-reduction process.

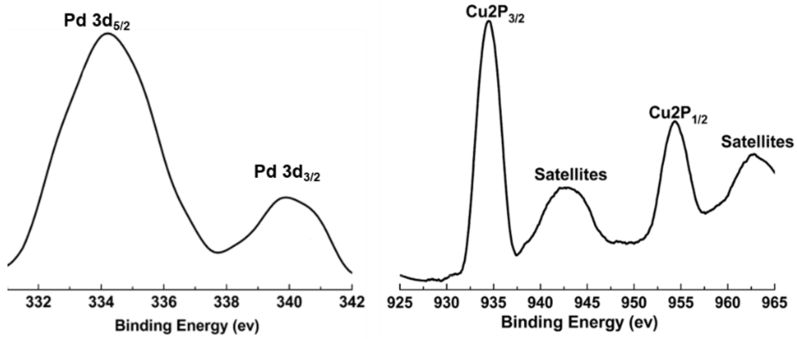

Supplementary Figure 7. XPS spectra of Pd(0) (left) and Cu(II) (right) species in **3**. The observation of Pd $d_{5/2}$ and $d_{3/2}$ peaks at 334.5 and 341.0 eV demonstrated the existence of Pd(0) in **3**. In addition, the two peaks at 934.8 and 954.5 eV in XPS spectrum were assigned to Cu $2p_{3/2}$ and $2p_{1/2}$, indicating that the oxidation state of Cu species in **3** did not change.

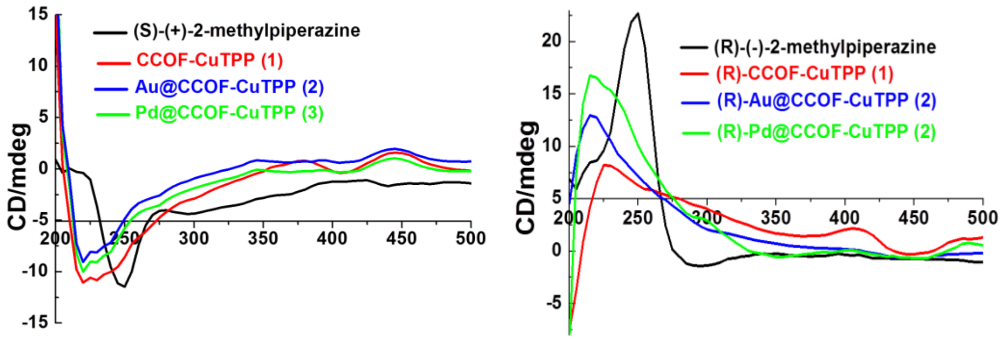

Supplementary Figure 8. Left: CD spectra of *S*-(+)-2-methylpiperazine, and **1-3**. Right: For further confirming the chirality of **1-3**, the corresponding *R*-CCOF-based **1-3** from *R*-(-)-2-methylpiperazine, Cu-TBrPP and corresponding M NP were prepared. The positive dichroic signals at 250 nm for (*R*)-methylpiperazine, 225 nm for *R*-CCOF, 220 nm for Au@*R*-CCOF and 220 nm for Pd@*R*-CCOF were observed, while the negative Cotton effect at 445 nm for *R*-CCOF, 455 nm for Au@*R*-CCOF and 460 nm for Pd@*R*-CCOF were also detected in their CD spectra (right).

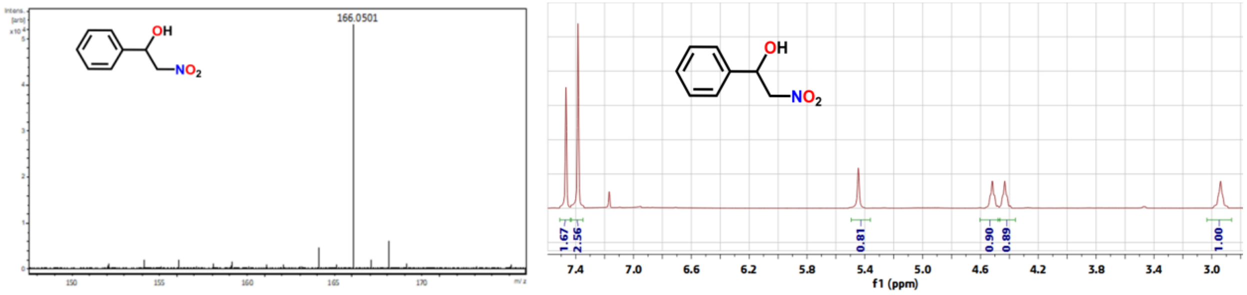

Supplementary Figure 9. ESI-MS and ^{1}H NMR spectra for the nitroaldol product generated from the model one-pot asymmetric Henry reaction. MS (ESI[+]): exact mass calcd for $C_8H_9NO_3$, 167.0582 ($[M+H]^+$). Found: 166.0501.

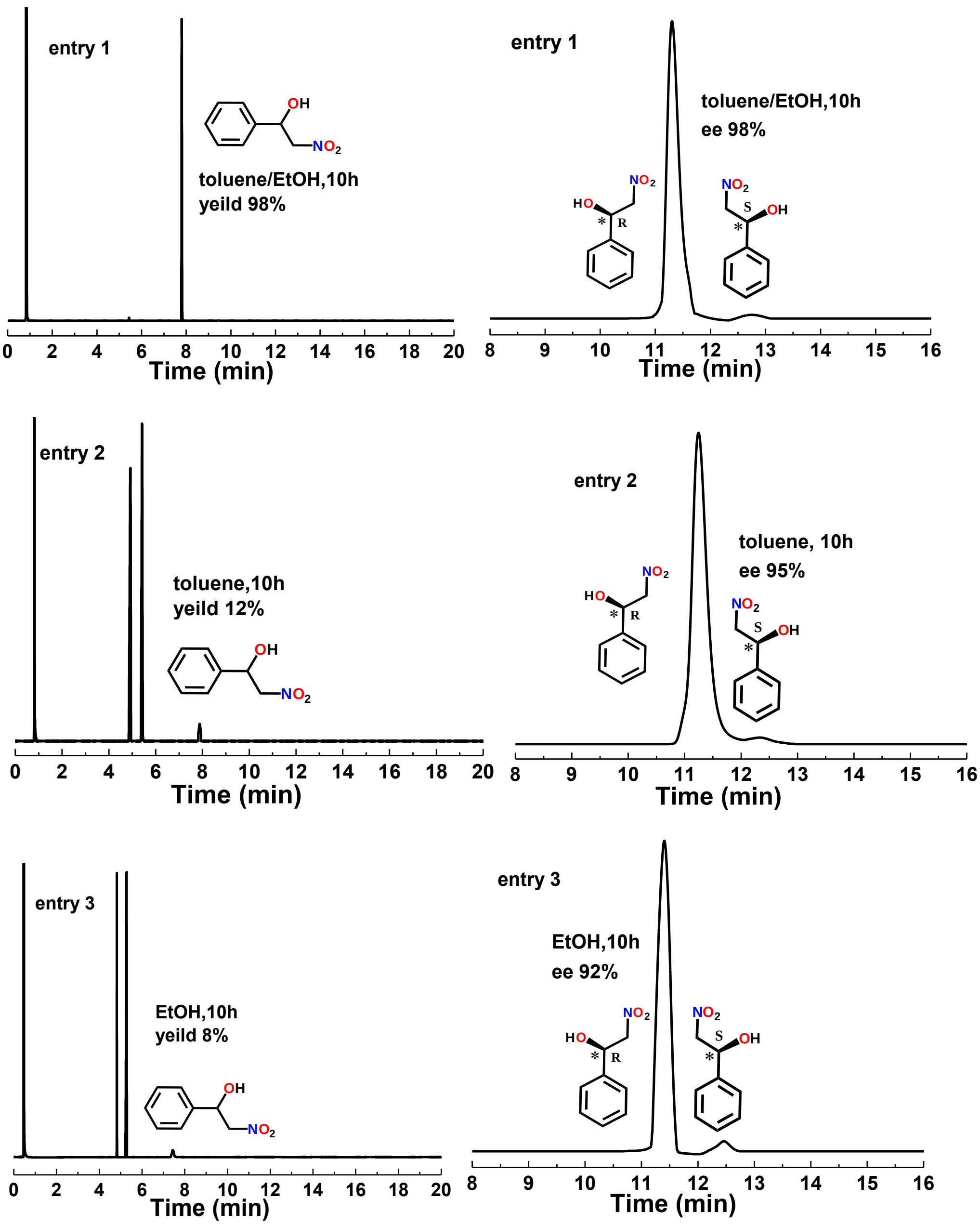

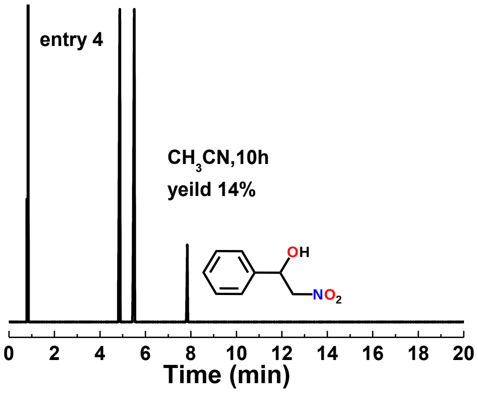
entry 4
CH_3CN,10h
yeild 14%
OH
NO_2
0 2 4 6 8 10 12 14 16 18 20
Time (min)

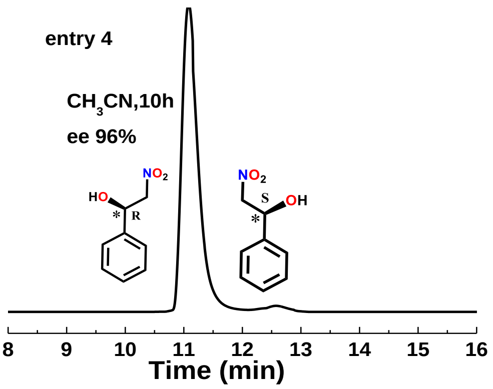
entry 4
CH_3CN,10h
ee 96%
HO
NO_2
R
S
OH
8 9 10 11 12 13 14 15 16
Time (min)

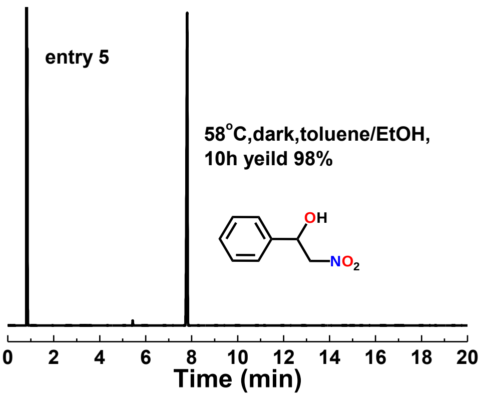
entry 5
58°C,dark,toluene/EtOH,
10h yeild 98%
OH
NO_2
0 2 4 6 8 10 12 14 16 18 20
Time (min)

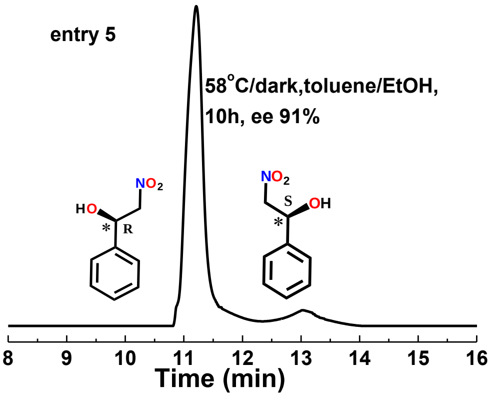
entry 5
58°C/dark,toluene/EtOH,
10h, ee 91%
HO
NO_2
R
S
OH
8 9 10 11 12 13 14 15 16
Time (min)

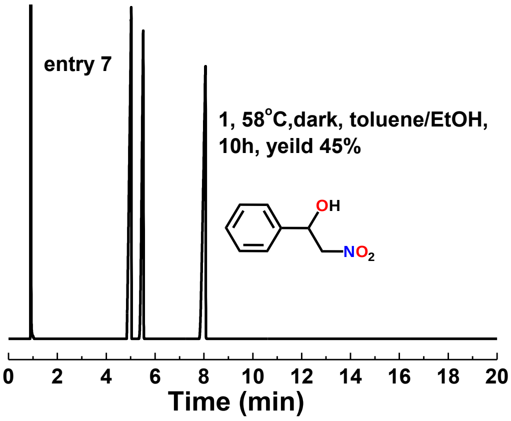
entry 7
1, 58°C,dark, toluene/EtOH,
10h, yeild 45%
OH
NO_2
0 2 4 6 8 10 12 14 16 18 20
Time (min)

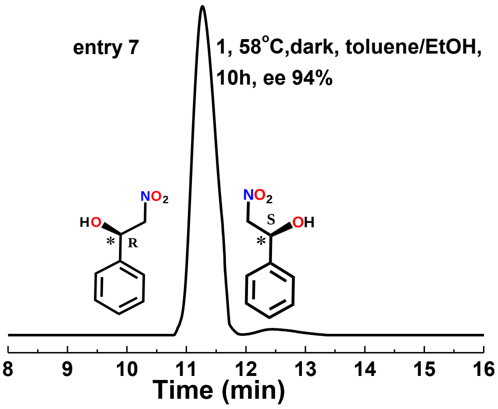
entry 7
1, 58°C,dark, toluene/EtOH,
10h, ee 94%
HO
NO_2
R
S
OH
8 9 10 11 12 13 14 15 16
Time (min)

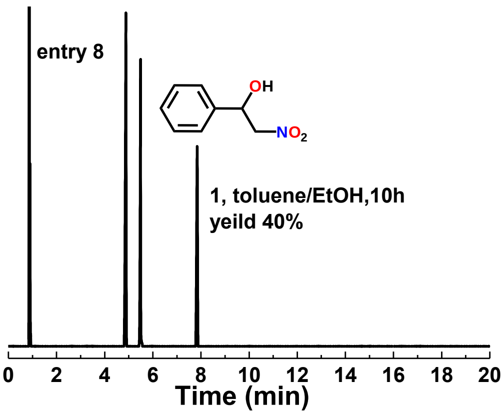
entry 8
OH
NO_2
1, toluene/EtOH,10h
yeild 40%
0 2 4 6 8 10 12 14 16 18 20
Time (min)

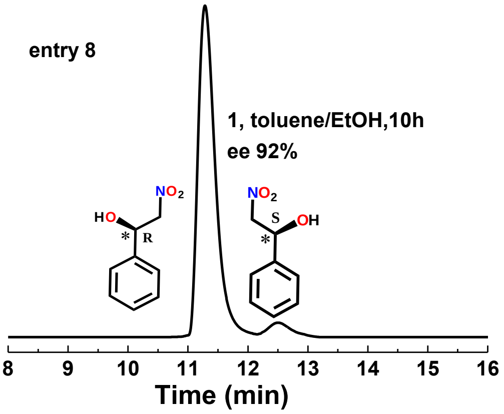
entry 8
1, toluene/EtOH,10h
ee 92%
HO
NO_2
R
S
OH
8 9 10 11 12 13 14 15 16
Time (min)

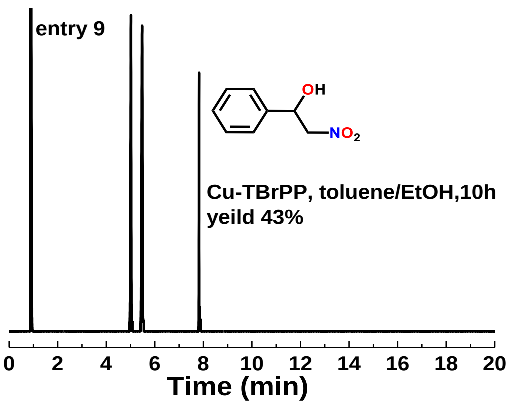
entry 9
OH
NO2
Cu-TBrPP, toluene/EtOH,10h
yeild 43%
0 2 4 6 8 10 12 14 16 18 20
Time (min)

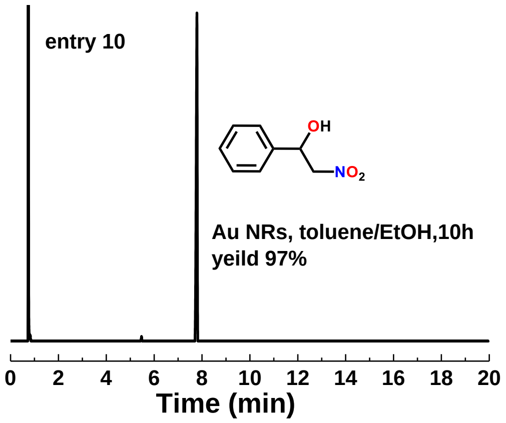
entry 10
OH
NO2
Au NRs, toluene/EtOH,10h
yeild 97%
0 2 4 6 8 10 12 14 16 18 20
Time (min)

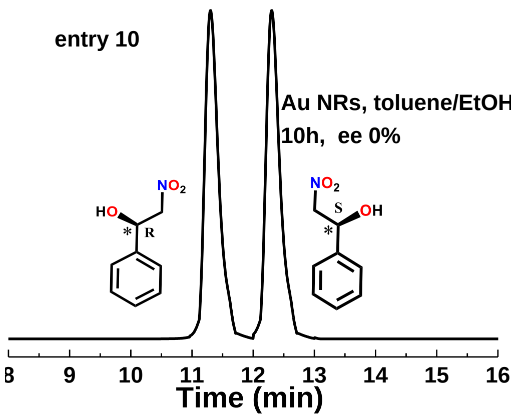
entry 10
Au NRs, toluene/EtOH
10h, ee 0%
NO2
HO
R
NO2
S
OH
9 10 11 12 13 14 15 16
Time (min)

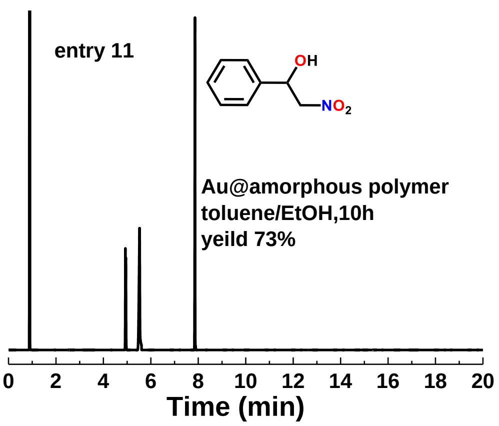
entry 11
OH
NO2
Au@amorphous polymer
toluene/EtOH,10h
yeild 73%
0 2 4 6 8 10 12 14 16 18 20
Time (min)

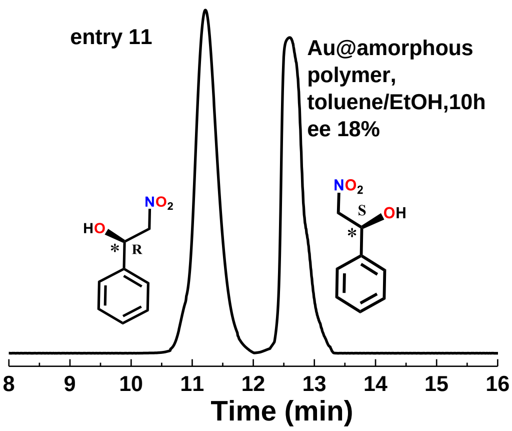
entry 11
Au@amorphous
polymer,
toluene/EtOH,10h
ee 18%
NO2
HO
R
NO2
S
OH
8 9 10 11 12 13 14 15 16
Time (min)

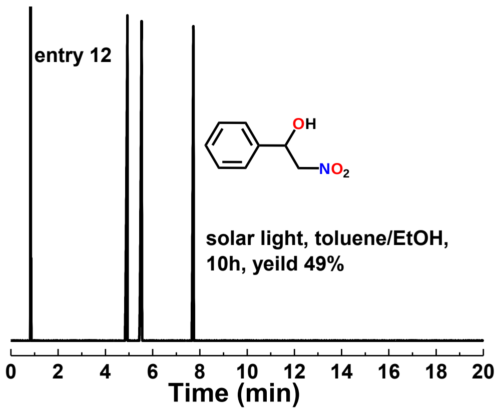
entry 12
OH
NO2
solar light, toluene/EtOH,
10h, yeild 49%
0 2 4 6 8 10 12 14 16 18 20
Time (min)

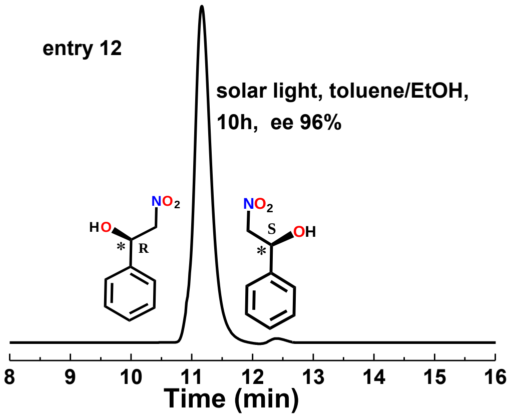
entry 12
solar light, toluene/EtOH,
10h, ee 96%
NO2
HO
R
NO2
S
OH
8 9 10 11 12 13 14 15 16
Time (min)

Supplementary Figure 10. Yield and enantiomeric excess of the model one-pot asymmetric Henry reaction between benzyl alcohol and nitromethane (for Table 1). Yield was determined by the GC on HP-5 column, enantiomeric excess was determined by HPLC with a Chiralcel OD-H column (90 : 10 = *n*-hexane : isopropanol, 1.0 mL min^{-1}, 230 nm), respectively.

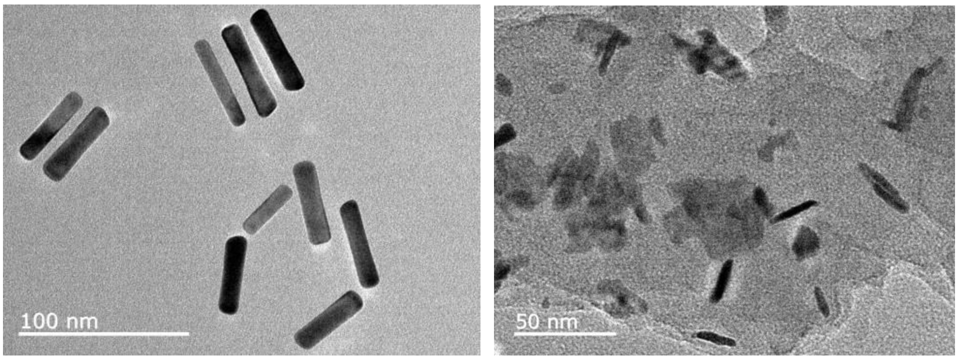

Supplementary Figure 11. TEM images of the Au NR (left) and the Au@amorphous polymer (right) which were used to catalyze one-pot asymmetric model Henry reaction between benzyl alcohol and nitromethane.

Supplementary Table 4. Summary of the typical asymmetric Henry reactions for PhCHO with CH_3NO_2.

catalyst	conditions	recycle	yield (ee)	TON	ref.
silica-supported (SBA-15) copper complex	EtOH, organic bases(S)/r.t./40 h	3	97 (97)	9.7	2
iPr-bipiperidine/$Cu(OAc)_2$	MeOH/H_2O/-25 °C/40 h	0	93 (94)	9.3	3
bis(oxazoline)-$Cu(OAc)_2$	EtOH /r.t./22 h	0	76 (94)	15.2	4
(*R, R*)-Cu/Sm/aminophenol sulfonamide	THF/-30 °C/90 h	0	90 (93)	9.0	5
sulfolobus tokodaii (ST0779)	H_2O, TBME 1:4/40 °C/90 h	0	34 (17)		6
pH chitosan hydrogel beads (CSHB)	H_2O/r.t./24 h	3	91 (99)	5353	7
[(Bisurea–salen)Co]	CH_2Cl_2, DIEPA/-70 °C/24 h	0	85 (97)	17	8
1,2-amino phosphinamide-Zn(II)	C_7H_8/-20 °C/48 h	0	99 (92)	9.9	9
trans-*N*,*N*'-bis-biphenyl-4-ylmethyl-cyclohexane-1,2-diamine-$CuCl_2$	AcOEt/Et_3N/0 °C/24 h	0	87 (91)	8.7	10
bis(thiourea) (R_p)-**1**	THF/i-Pr_2NEt/0 °C/24 h	0	57 (90)	11.4	11
per-6-ABCD	ACN/H_2O (1:1)/−20 °C/7 h	6	98 (90)	0.98	12
$(CuOTf)_2$-(+)-tetrahydrosalen ligand	methanol/40 °C/20 h	0	90 (92)	9.0	13
Cu(I)-[H_4]salen	4 Å MS, MeOH/ 45 °C/60 h	0	64 (92)	6.4	14
amino alcohol-copper(II)	Et_2O/r.t./48 h	0	66 (98)	26.4	15
NA-MgO	THF/25 °C/10 h	4	95 (90)	36.5	16
bispidine-$CuCl_2$	NEt_3, THF, $MeNO_3$/-20 °C/42 h	0	99 (98)	49.5	17
bisoxazolidine-CuOAc	EtOH/-15 °C/16 h	0	93 (89)	9.3	18
Thiourea compound	THF/-20 °C/48 h	0	90 (92)	9.0	19
Pd@CCOF-MPC	K_2CO_3, EtOH /r. t./8 h	4	97 (95)	48.5	20
**Au@CCOF-CuTPP (2)**	(reaction starting from benzyl alcohol), K_2CO_3/PhMe,EtOH/*hv*/10 h	4	98 (98)	98	**this work**

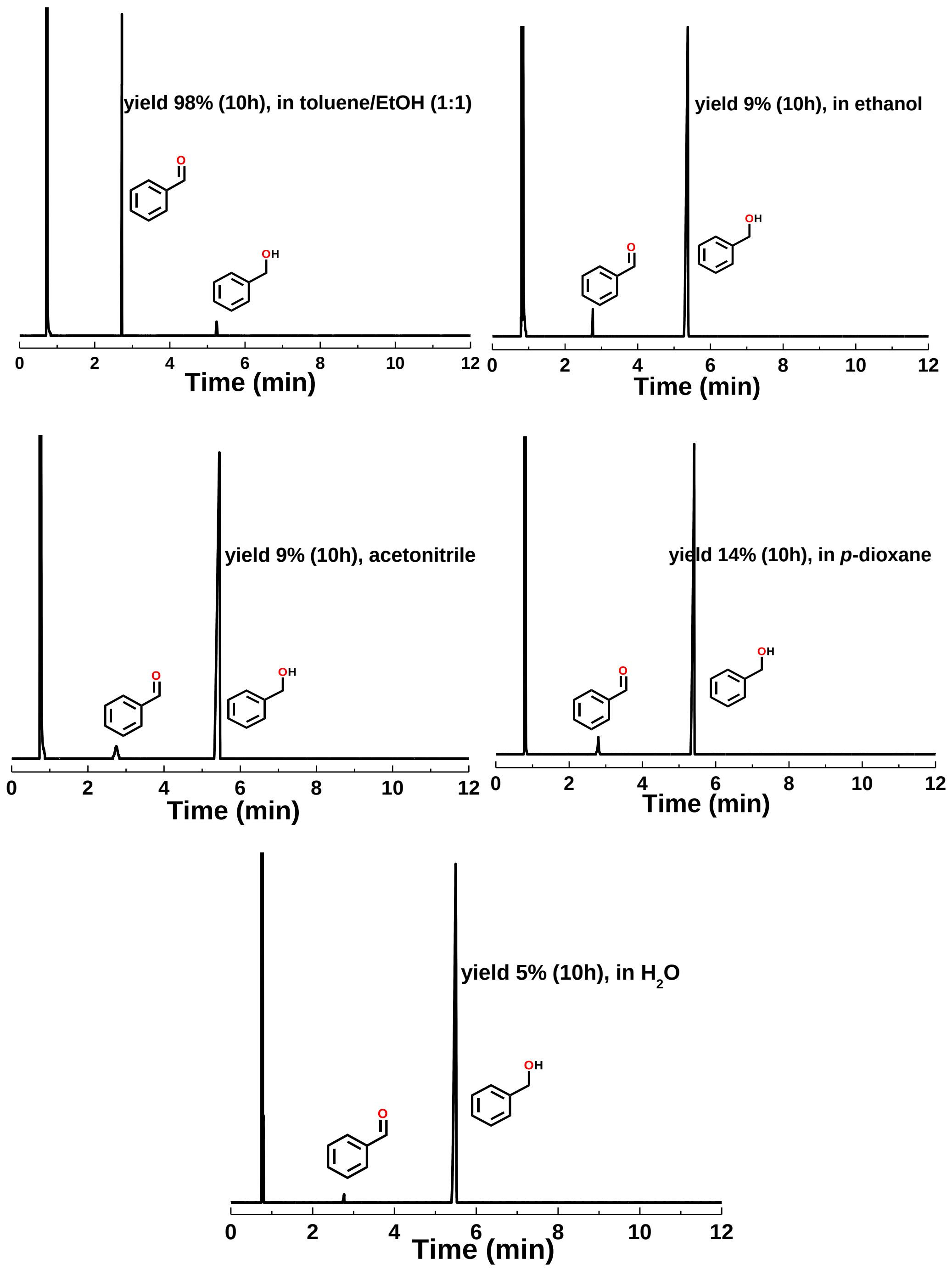

Supplementary Figure 12. GC analysis of the **2**-catalyzed selective benzyl alcohol oxidation in air using different solvents with light irradiation (λ > 400 nm, 300 W xenon with a power density of 2.5 W cm^{-2}).

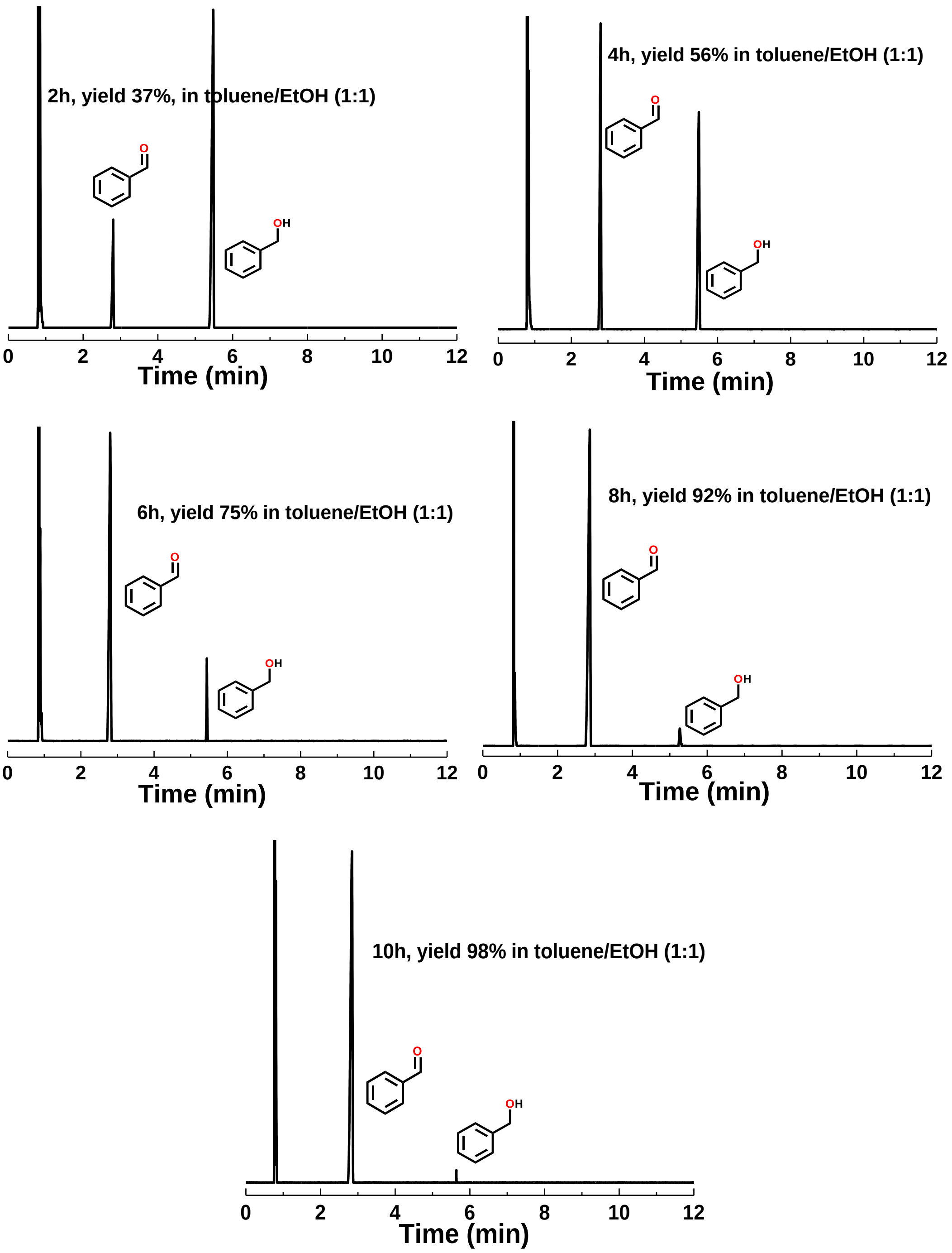

Supplementary Figure 13. GC analysis of the selective benzyl alcohol oxidation in air with light irradiation (λ > 400 nm, 300 W xenon with a power density of 2.5 W cm^{-2}) catalyzed by **2**: the relationship between time and yield.

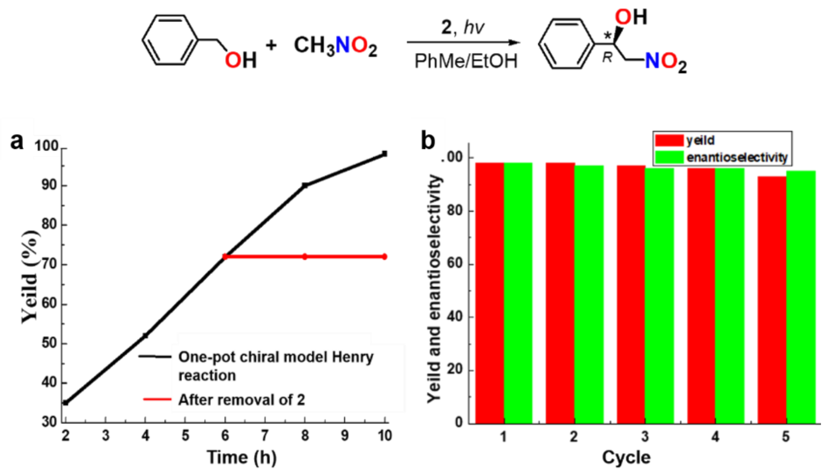

Supplementary Figure 14. **a**, Reaction time examination (black line) and leaching test (red line) for model one-pot asymmetric Henry reaction. **b**, Catalytic cycles. After each run, the catalyst was collected by centrifugation, washed with ethanol and dichloromethane, dried at 90 °C in vacuum for the next catalytic run under the same conditions. Reaction conditions: **2** (6 mg, 1 mol % Au equiv), aromatic alcohol (0.5 mmol), nitromethane (1.5 mmol), K_2CO_3 (1.5 mmol), toluene/EtOH (1:1, 2 mL), room temperature, visible light (λ > 400 nm, 300 W xenon with a power density of 2.5 W cm^{-2}), 10 h. The solid catalyst was filtrated from the reaction solution after 6 h, whereas the filtrate was transferred to a new vial and the reaction was carried out under the same conditions for an additional 4 h. Yield was determined by the GC measurement on HP-5 column, and enantiomeric excess was determined by HPLC with a Chiralcel OD-H column (90 : 10 = *n*-hexane : isopropanol, 1.0 mL min^{-1}, 230 nm), respectively.

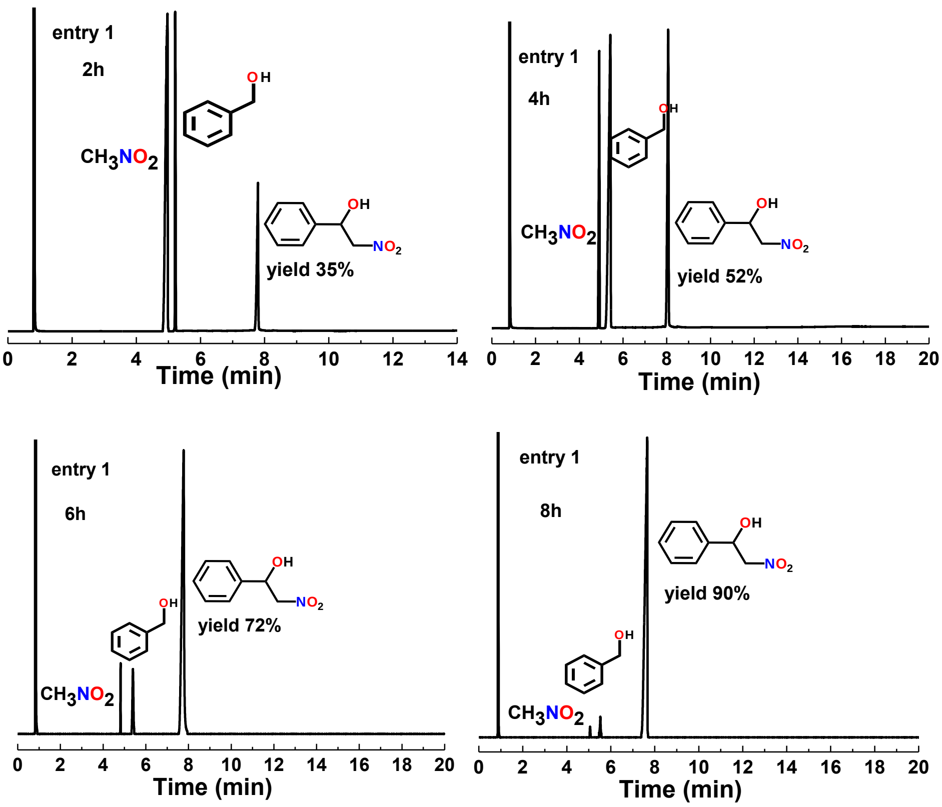

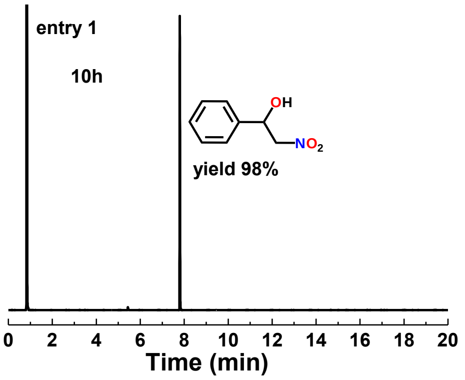

Supplementary Figure 15. GC analysis for the model one-pot asymmetric Henry reaction catalyzed by **2**: the relationship between time and yield.

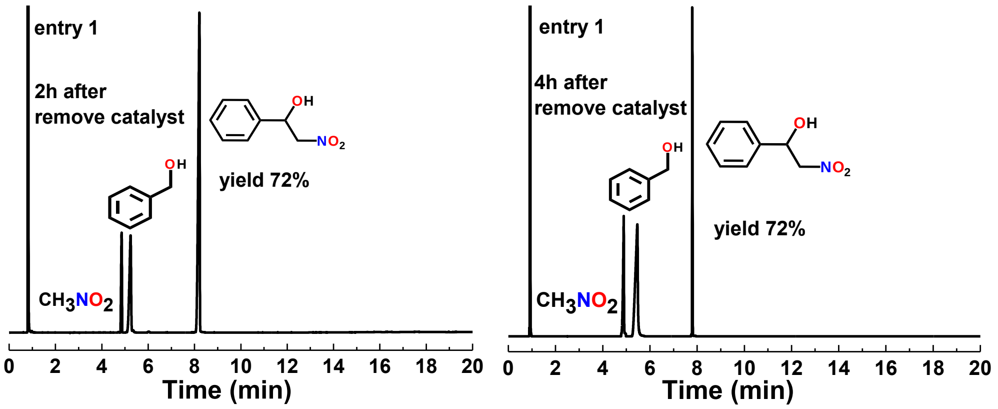

Supplementary Figure 16. Hot leaching test. The GC analysis for the model one-pot asymmetric Henry reaction without **2**.

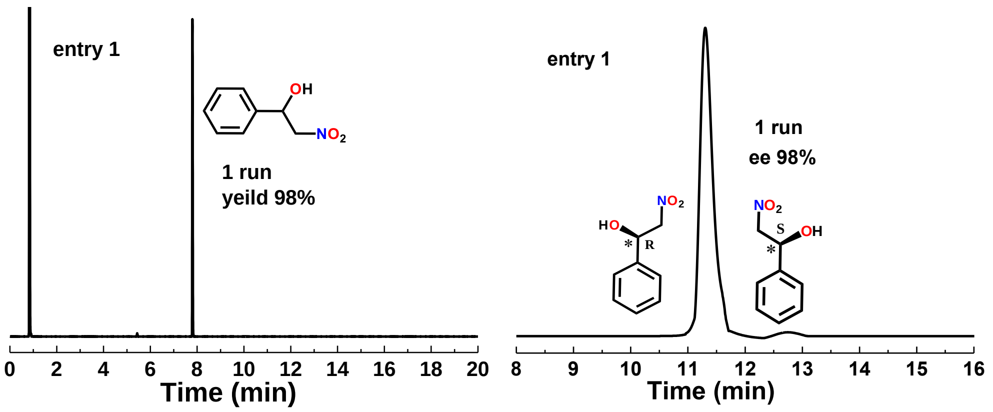

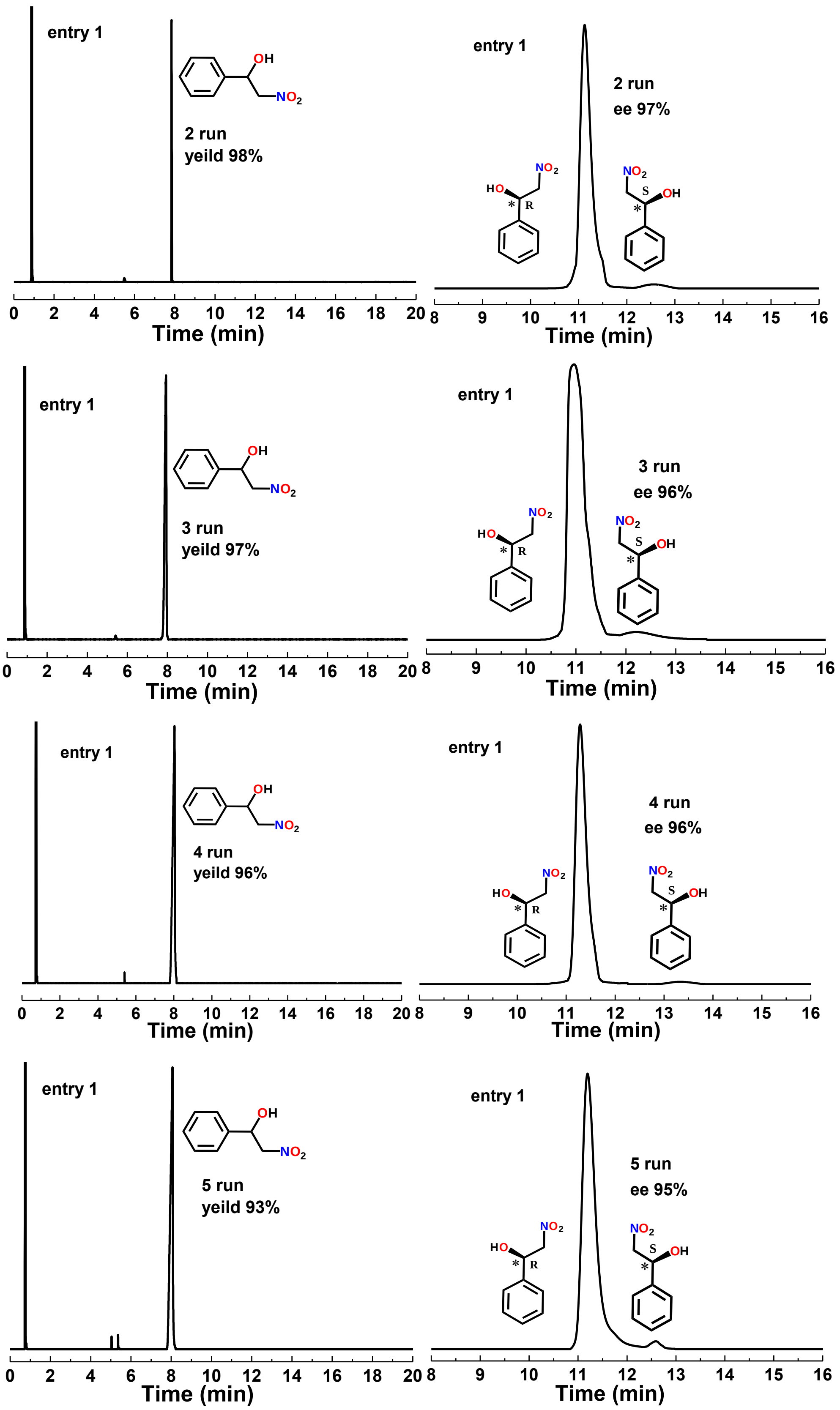

Supplementary Figure 17. Catalytic cycles for the model one-pot asymmetric Henry reaction. Yield was determined by the GC on HP-5 column, and enantiomeric excess was determined by HPLC with a Chiralcel OD-H column (90 : 10 = n-hexane : isopropanol, 1.0 mL min^{-1}, 230 nm).

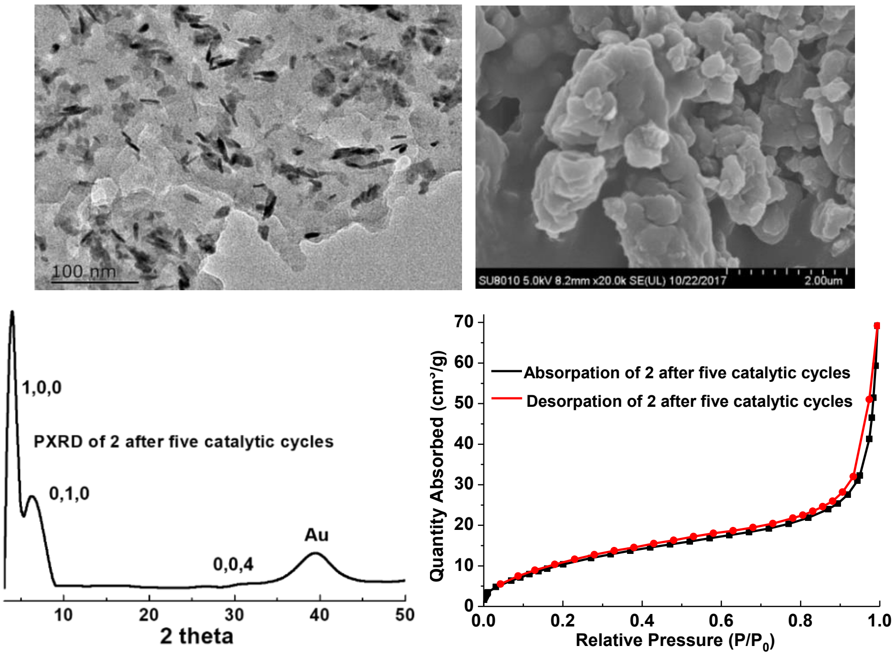

Supplementary Figure 18. TEM, SEM, PXRD and the gas adsorption-desorption of **2** after five catalytic cycles. The Au amount in **2** determined by ICP is 15.1 wt% after five catalytic cycles. No structural and morphology changes were observed after five catalytic cycles. The adsorption-desorption value slightly decreased from 79.4 cm^3 g^{-1} (before catalysis) to 69.2 cm^3 g^{-1} for **2** after five catalytic cycles.

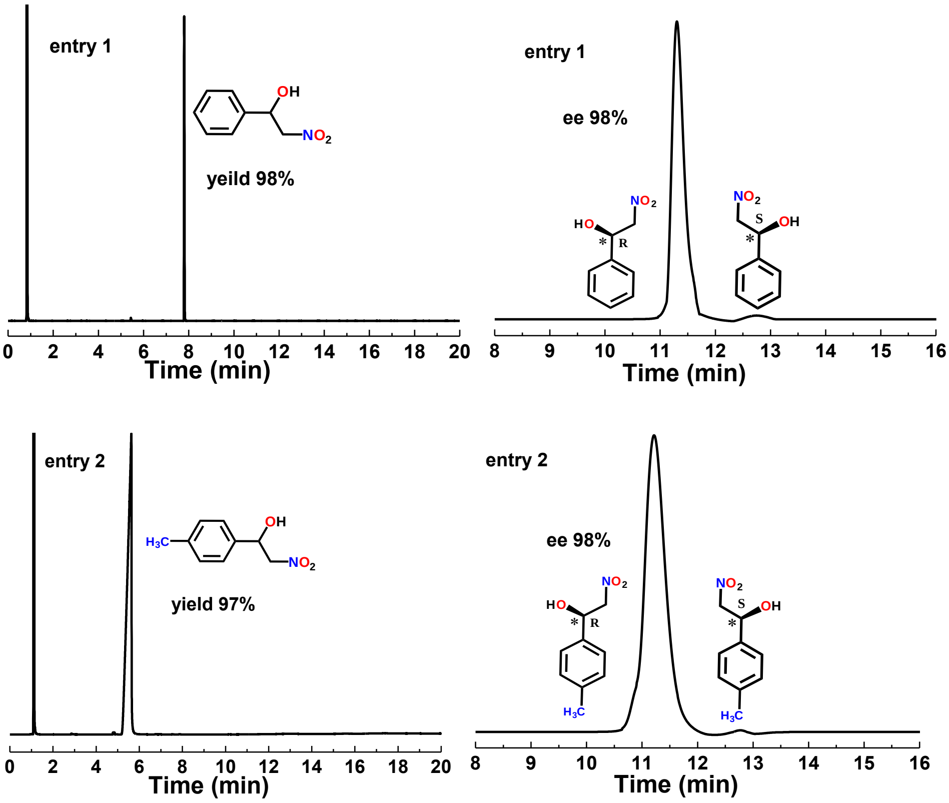

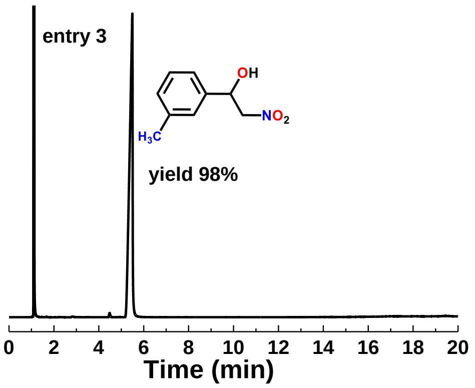
entry 3
OH
NO_2
H_3C
yield 98%
0 2 4 6 8 10 12 14 16 18 20
Time (min)

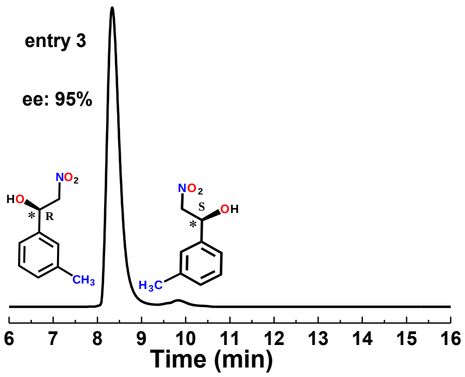
entry 3
ee: 95%
HO
NO_2
R
CH_3
S
OH
H_3C
6 7 8 9 10 11 12 13 14 15 16
Time (min)

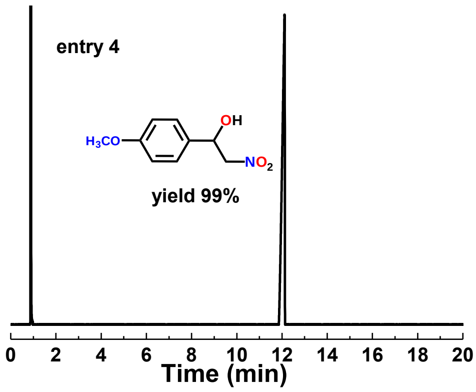
entry 4
H_3CO
OH
NO_2
yield 99%
0 2 4 6 8 10 12 14 16 18 20
Time (min)

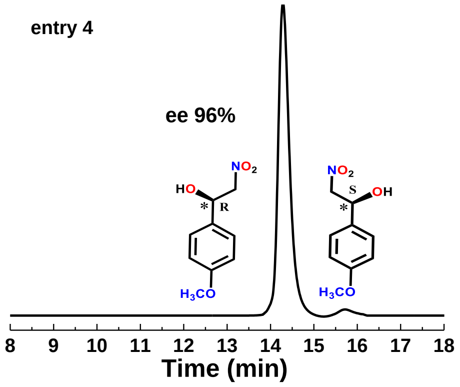
entry 4
ee 96%
HO
NO_2
R
H_3CO
S
OH
8 9 10 11 12 13 14 15 16 17 18
Time (min)

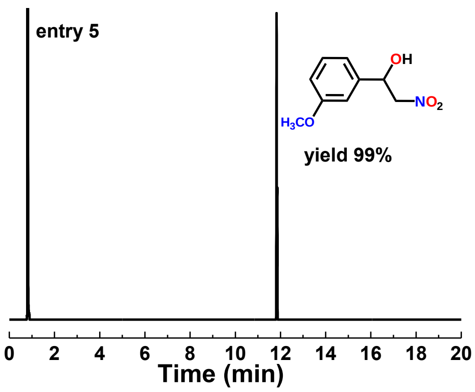
entry 5
OH
NO_2
H_3CO
yield 99%
0 2 4 6 8 10 12 14 16 18 20
Time (min)

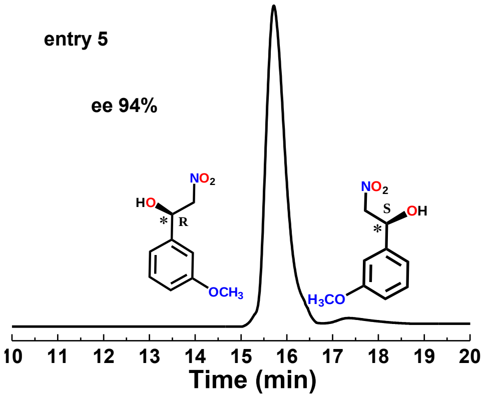
entry 5
ee 94%
HO
NO_2
R
OCH_3
S
OH
H_3CO
10 11 12 13 14 15 16 17 18 19 20
Time (min)

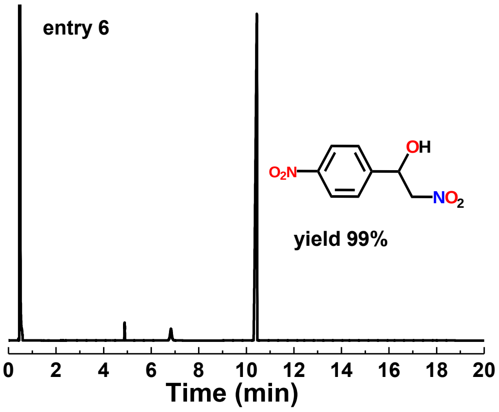
entry 6
O_2N
OH
NO_2
yield 99%
0 2 4 6 8 10 12 14 16 18 20
Time (min)

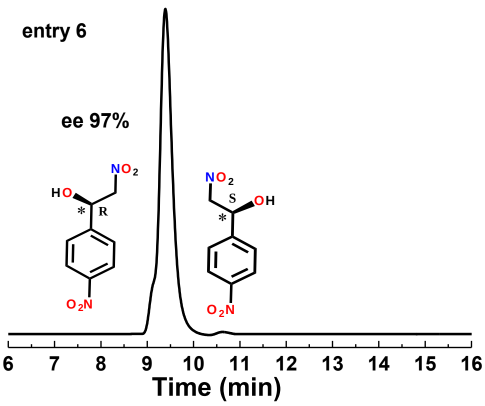
entry 6
ee 97%
HO
NO_2
R
O_2N
S
OH
6 7 8 9 10 11 12 13 14 15 16
Time (min)

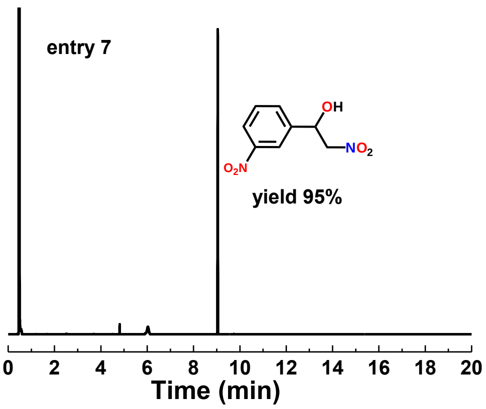
entry 7
yield 95%
OH
NO_2
O_2N
0 2 4 6 8 10 12 14 16 18 20
Time (min)

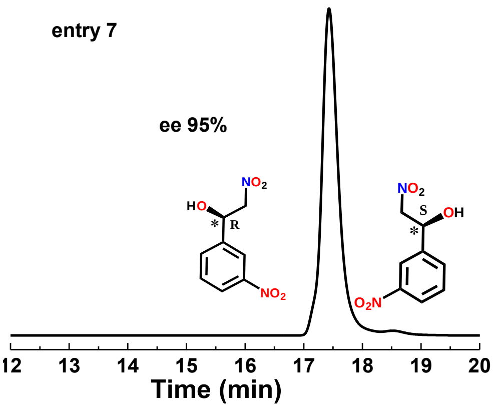
entry 7
ee 95%
HO
NO_2
R
S
OH
O_2N
12 13 14 15 16 17 18 19 20
Time (min)

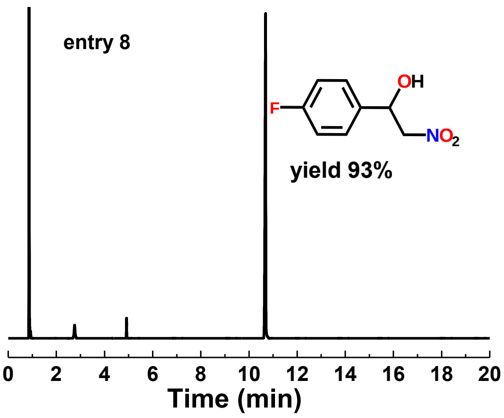
entry 8
yield 93%
F
OH
NO_2
0 2 4 6 8 10 12 14 16 18 20
Time (min)

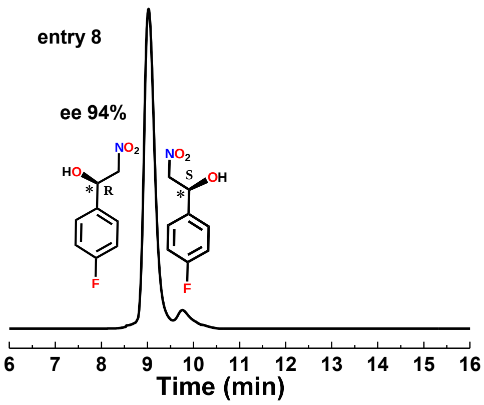
entry 8
ee 94%
HO
NO_2
R
S
OH
F
6 7 8 9 10 11 12 13 14 15 16
Time (min)

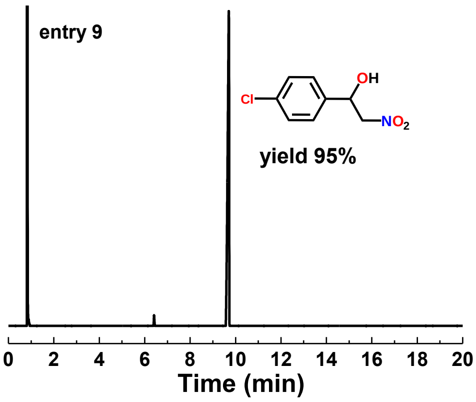
entry 9
yield 95%
Cl
OH
NO_2
0 2 4 6 8 10 12 14 16 18 20
Time (min)

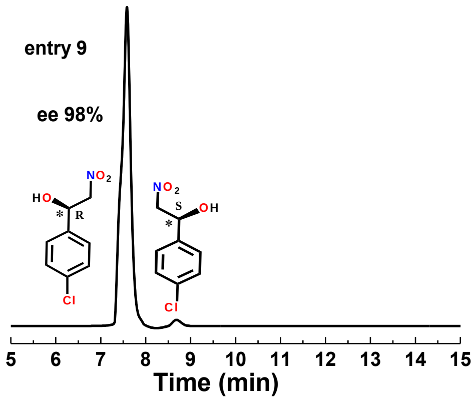
entry 9
ee 98%
HO
NO_2
R
S
OH
Cl
5 6 7 8 9 10 11 12 13 14 15
Time (min)

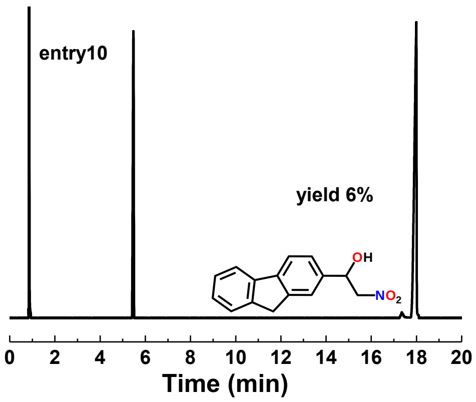
entry10
yield 6%
OH
NO_2
0 2 4 6 8 10 12 14 16 18 20
Time (min)

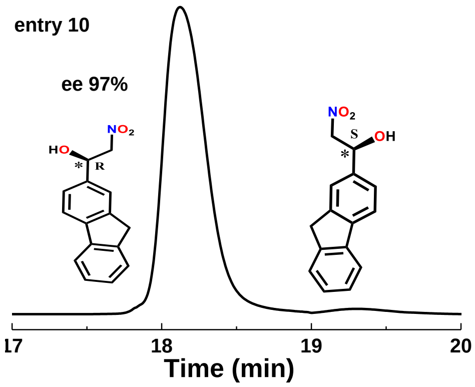
entry 10
ee 97%
HO
NO_2
R
S
OH
17 18 19 20
Time (min)

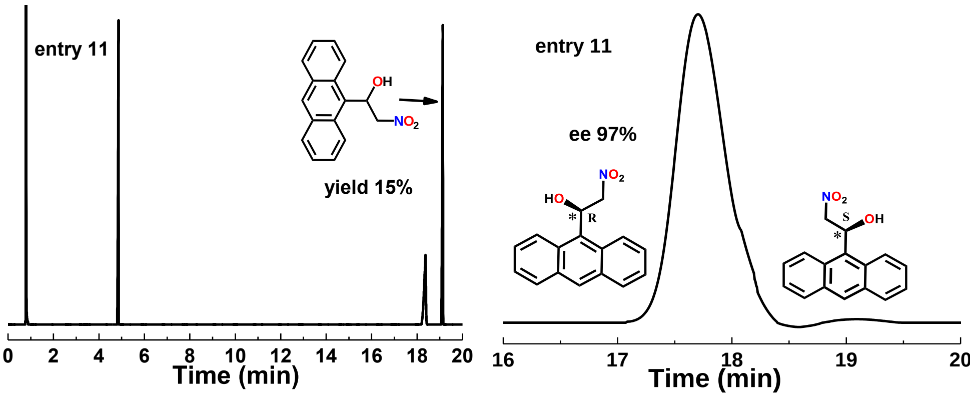

Supplementary Figure 19. GC and HPLC analysis for the expanded one-pot asymmetric Henry reactions catalyzed by **2** based on various substituted substrates (for Table 2).

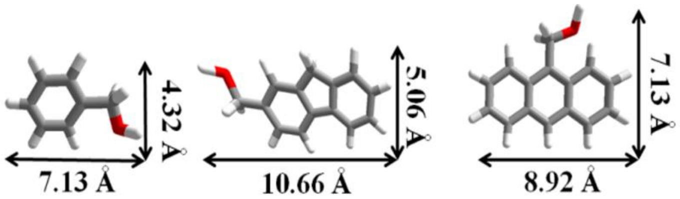

Supplementary Figure 20. The size of benzyl alcohol, 9H-fluorene-2-methanol and 9-anthracenemethanol based on their molecular structure.

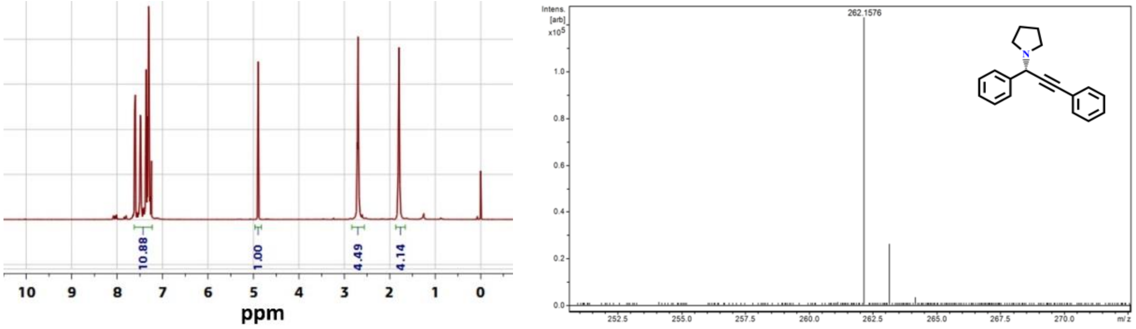

Supplementary Figure 21. ^{1}H NMR and ESI-MS spectra for the model one-pot asymmetric A^3-coupling product. MS (ESI$^+$): exact mass calcd for $C_{19}H_{19}N$, 261.1517 ([M+H$^+$]). Found: 262.1576.

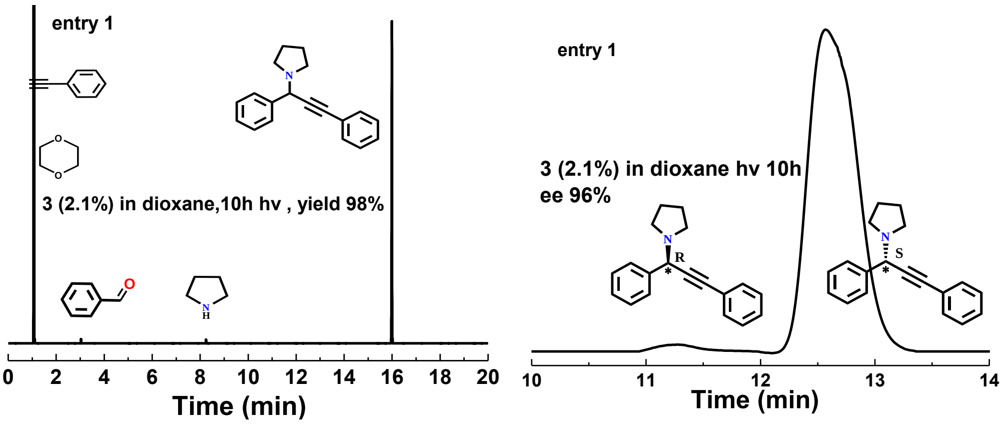

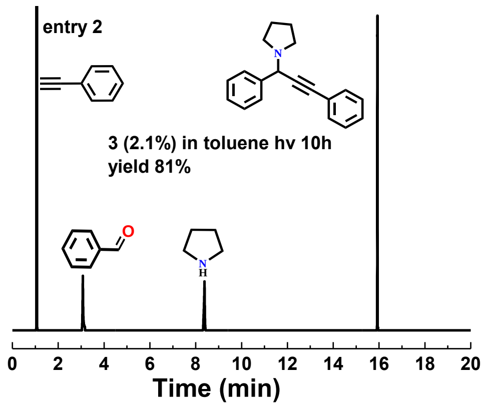
entry 2
3 (2.1%) in toluene hv 10h
yield 81%
0 2 4 6 8 10 12 14 16 18 20
Time (min)

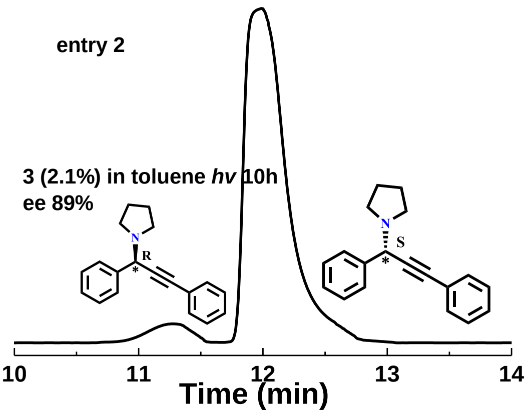
entry 2
3 (2.1%) in toluene hv 10h
ee 89%
R
S
10 11 12 13 14
Time (min)

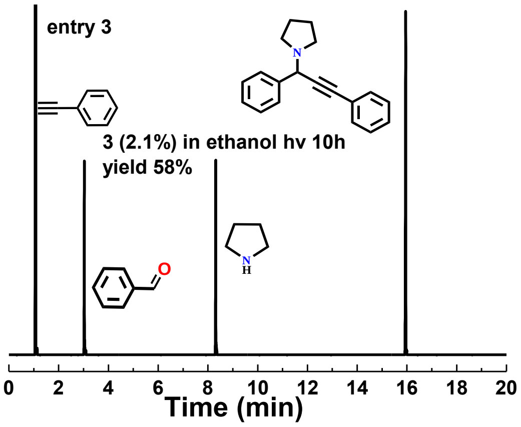
entry 3
3 (2.1%) in ethanol hv 10h
yield 58%
0 2 4 6 8 10 12 14 16 18 20
Time (min)

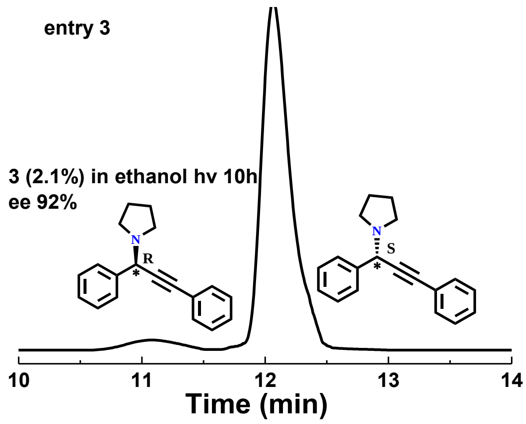
entry 3
3 (2.1%) in ethanol hv 10h
ee 92%
R
S
10 11 12 13 14
Time (min)

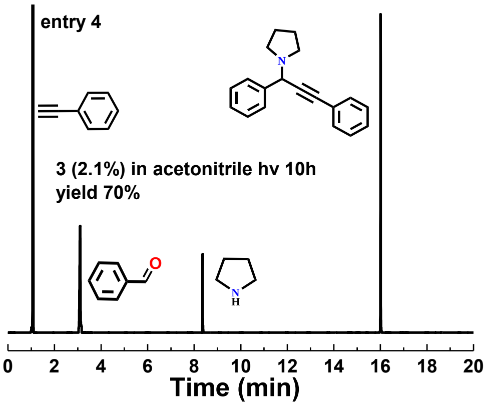
entry 4
3 (2.1%) in acetonitrile hv 10h
yield 70%
0 2 4 6 8 10 12 14 16 18 20
Time (min)

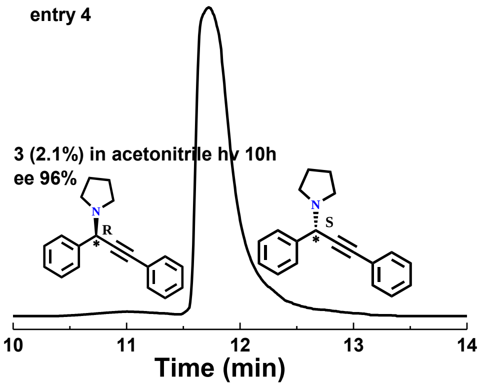
entry 4
3 (2.1%) in acetonitrile hv 10h
ee 96%
R
S
10 11 12 13 14
Time (min)

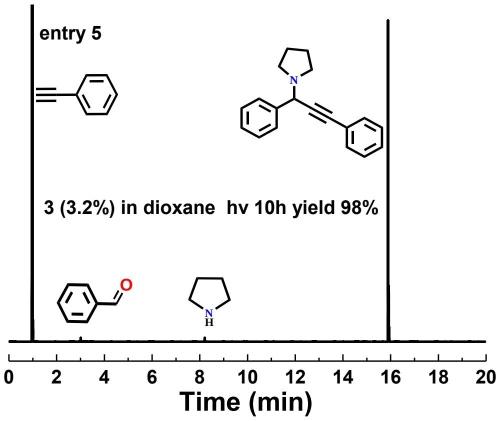
entry 5
3 (3.2%) in dioxane hv 10h yield 98%
0 2 4 6 8 10 12 14 16 18 20
Time (min)

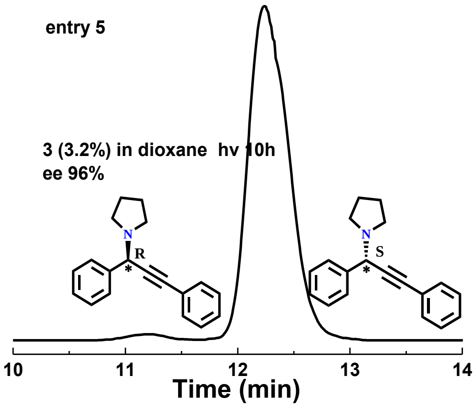
entry 5
3 (3.2%) in dioxane hv 10h
ee 96%
R
S
10 11 12 13 14
Time (min)

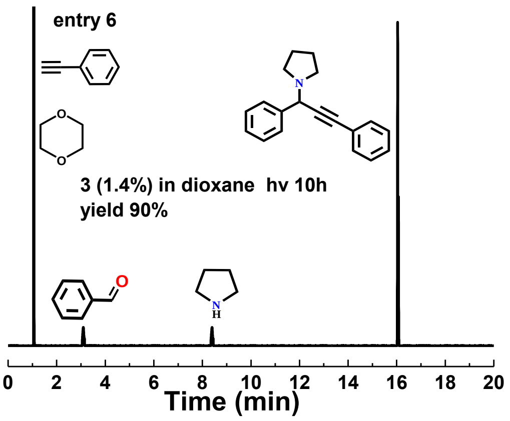
entry 6
3 (1.4%) in dioxane hv 10h
yield 90%
0 2 4 6 8 10 12 14 16 18 20
Time (min)

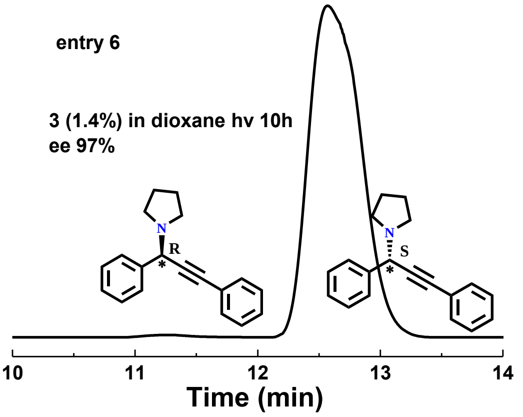
entry 6
3 (1.4%) in dioxane hv 10h
ee 97%
R
S
10 11 12 13 14
Time (min)

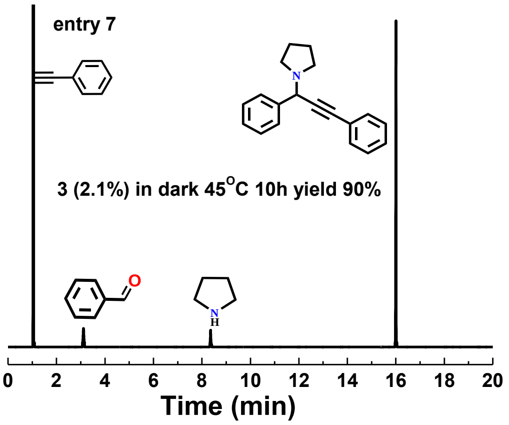
entry 7
3 (2.1%) in dark 45°C 10h yield 90%
0 2 4 6 8 10 12 14 16 18 20
Time (min)

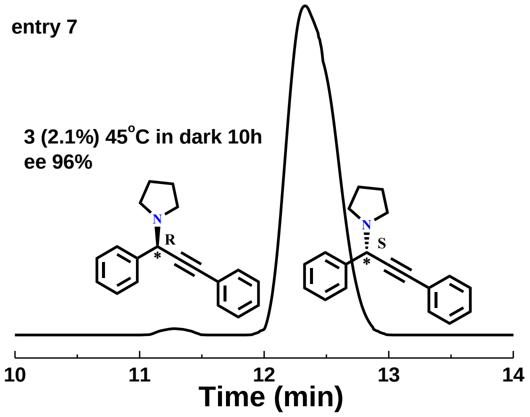
entry 7
3 (2.1%) 45°C in dark 10h
ee 96%
R
S
10 11 12 13 14
Time (min)

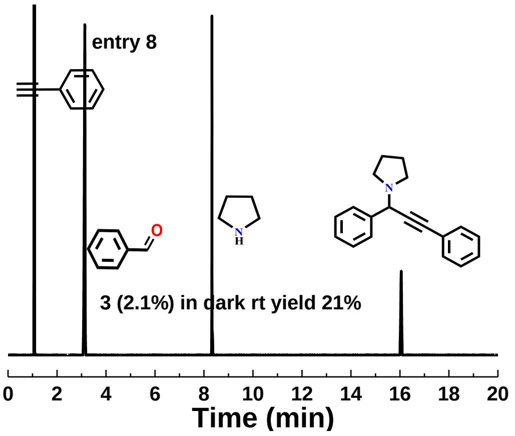
entry 8
3 (2.1%) in dark rt yield 21%
0 2 4 6 8 10 12 14 16 18 20
Time (min)

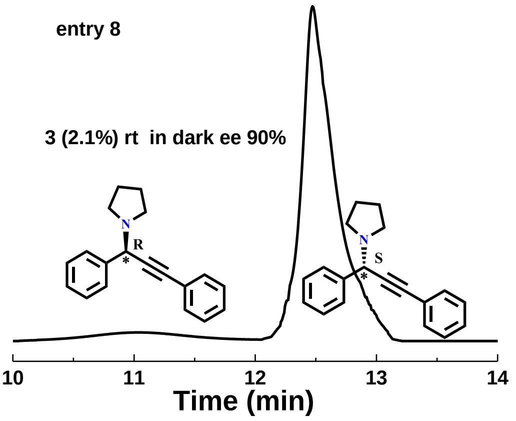
entry 8
3 (2.1%) rt in dark ee 90%
R
S
10 11 12 13 14
Time (min)

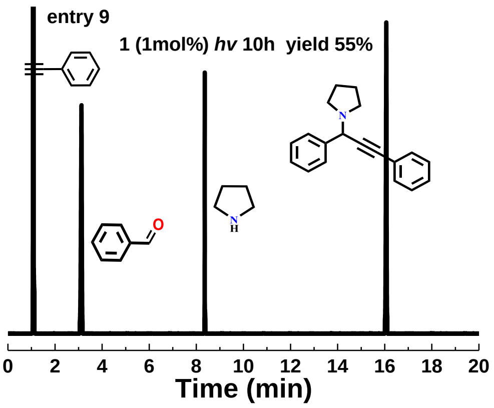
entry 9
1 (1mol%) hv 10h yield 55%
Time (min)

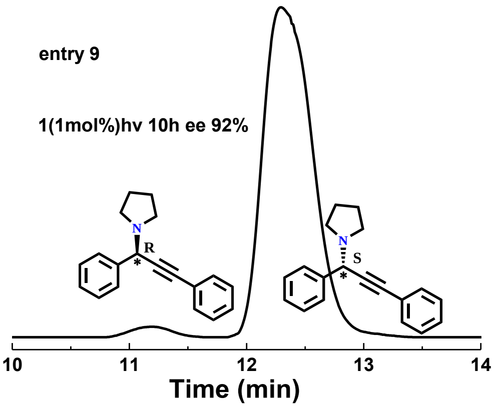
entry 9
1(1mol%)hv 10h ee 92%
R
S
Time (min)

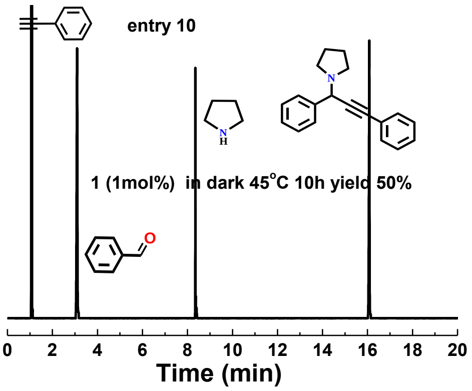
entry 10
1 (1mol%) in dark 45°C 10h yield 50%
Time (min)

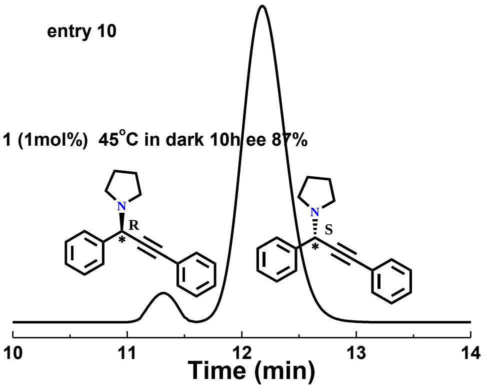
entry 10
1 (1mol%) 45°C in dark 10h ee 87%
R
S
Time (min)

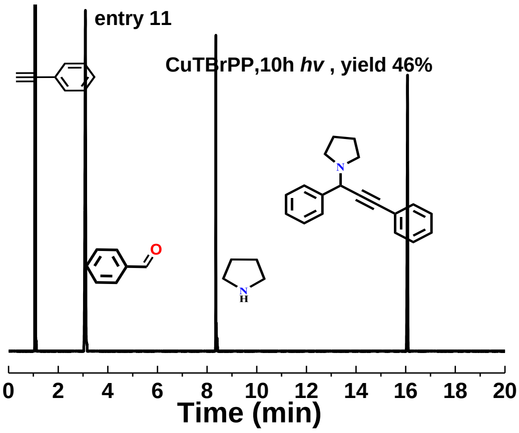
entry 11
CuTBrPP,10h hv , yield 46%
Time (min)

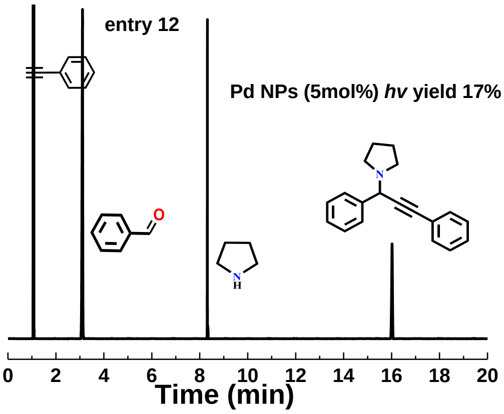
entry 12
Pd NPs (5mol%) hv yield 17%
Time (min)

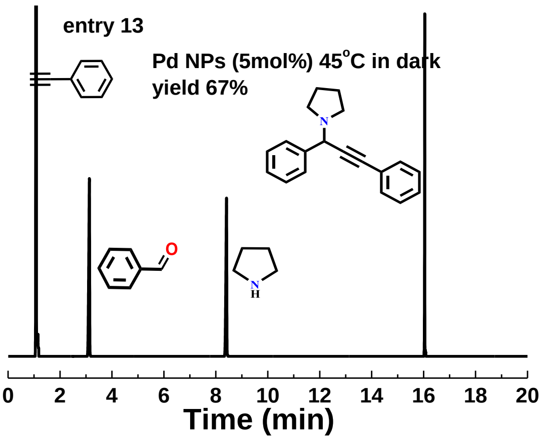
entry 13
Pd NPs (5mol%) 45°C in dark
yield 67%
Time (min)

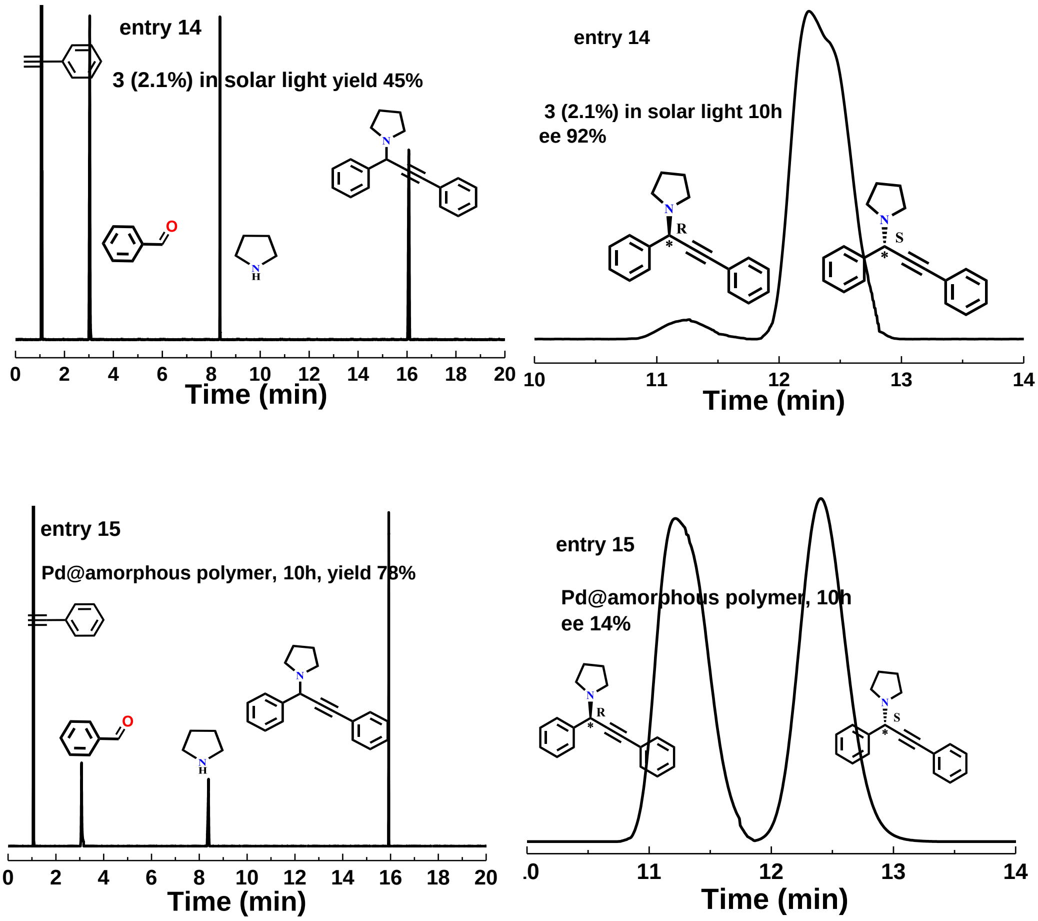

Supplementary Figure 22. GC and HPLC analysis of the model one-pot asymmetric A^3-coupling reaction was carried out under different conditions (for Table 3). Yield was determined by the GC on HP-5 column, and enantiomeric excess was determined by HPLC with a Chiralcel OJ-H column (99 : 1 = *n*-hexane : isopropanol, 1.0 mL min^{-1}, 254 nm).

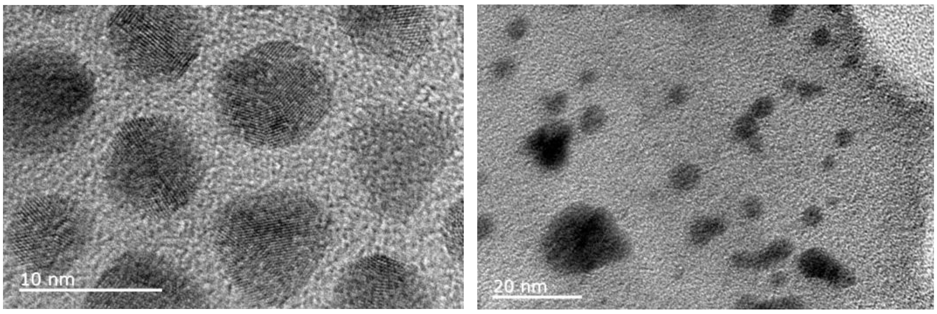

Supplementary Figure 23. TEM images of Pd NP (left) and Pd@amorphous polymer (right) used to catalyze the model A^3-coupling reaction.

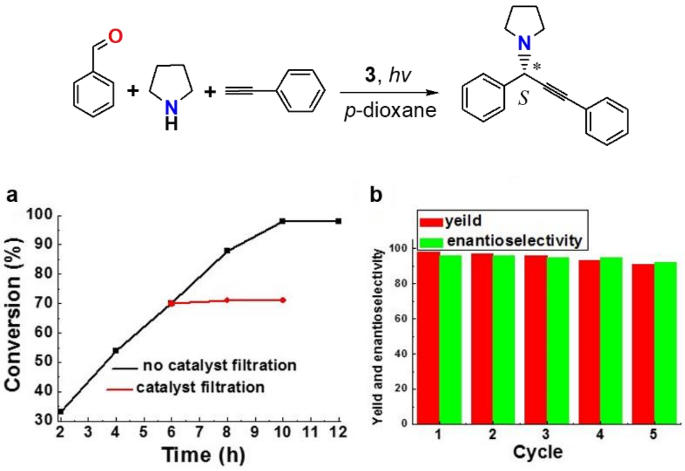

Supplementary Figure 24. a, Reaction time examination (black line) and leaching test (red line) for the **3**-catalyzed model one-pot chiral A[3]-coupling reaction. **b**, Catalytic cycles for the **3**-catalyzed model one-pot chiral A[3]-coupling reaction. After each run, **3** was collected by centrifugation, washed with ethanol and dichloromethane, dried at 90 °C in vacuum for the next catalytic run under the same conditions. Reaction conditions: under nitrogen, **3** (4.5 mg, 2.1 mol % Pd equiv), benzaldehyde (0.5 mmol), phenylacetylene (0.5 mmol) and pyrrolidine (0.75 mmol) in *p*-dioxane (2 mL) was stirred at room temperature under visible light irradiation (λ > 400 nm, 300 W xenon with a power density of 2.5 W cm^{-2}). The solid catalyst was filtrated from the reaction solution after 6 h, whereas the filtrate was transferred to a new vial and reaction was carried out under the same conditions for an additional 4 h. Yield was determined by the GC measurement on HP-5 column, and enantiomeric excess was determined by HPLC with a Chiralcel OJ-H column (99 : 1 = *n*-hexane : isopropanol, 1.0 mL min^{-1}, 254 nm), respectively.

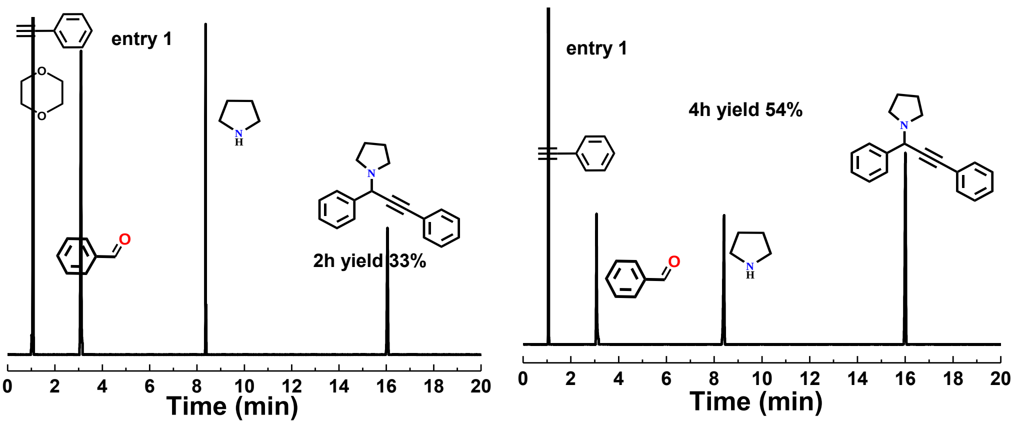

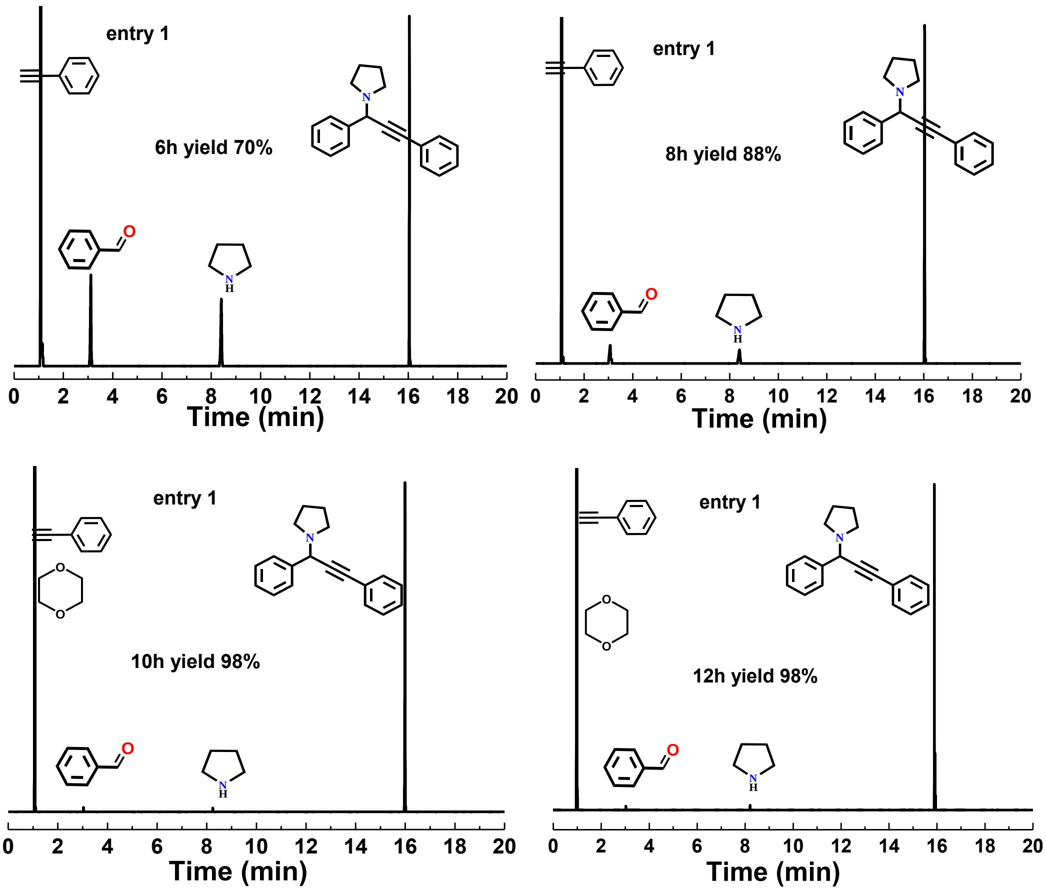

Supplementary Figure 25. GC analysis of the model one-pot asymmetric A[3]-coupling reaction catalyzed by **3**: the relationship between time and yield. Yield was determined by the GC on HP-5 column.

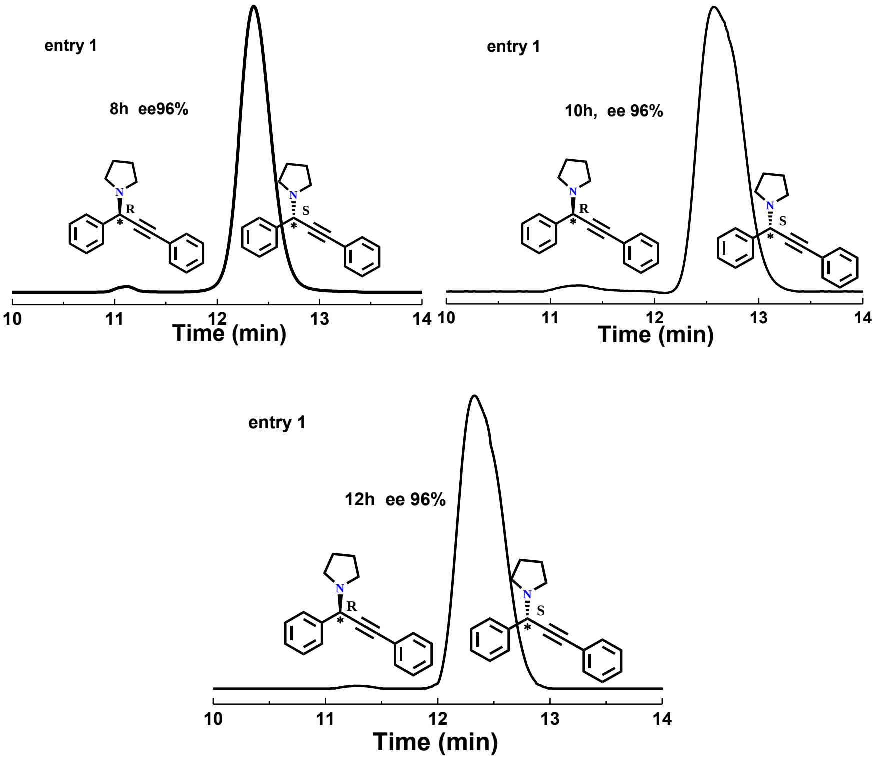

Supplementary Figure 26. The corresponding enantiomeric excess of the model one-pot asymmetric A^3-coupling reaction was monitored by HPLC with a Chiralcel OJ-H column (99 : 1 = *n*-hexane : isopropanol, 1.0 mL min^{-1}, 254 nm) at different time.

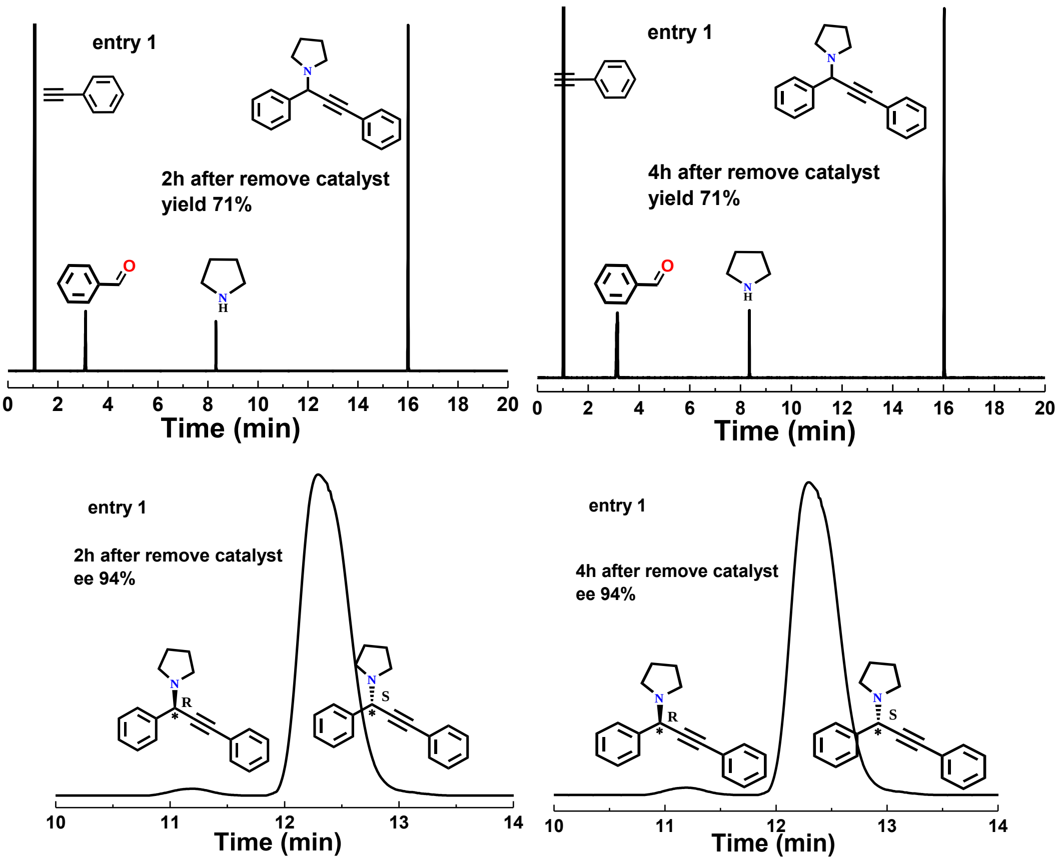

Supplementary Figure 27. Hot leaching test. The GC (yield) and HPLC analysis (ee) for the model one-pot asymmetric A^3-coupling reaction without **3**. Yield was determined by the GC on HP-5 column, and enantiomeric excess was determined by HPLC with a Chiralcel OJ-H column (99 : 1 = *n*-hexane : isopropanol, 1.0 mL min^{-1}, 254 nm).

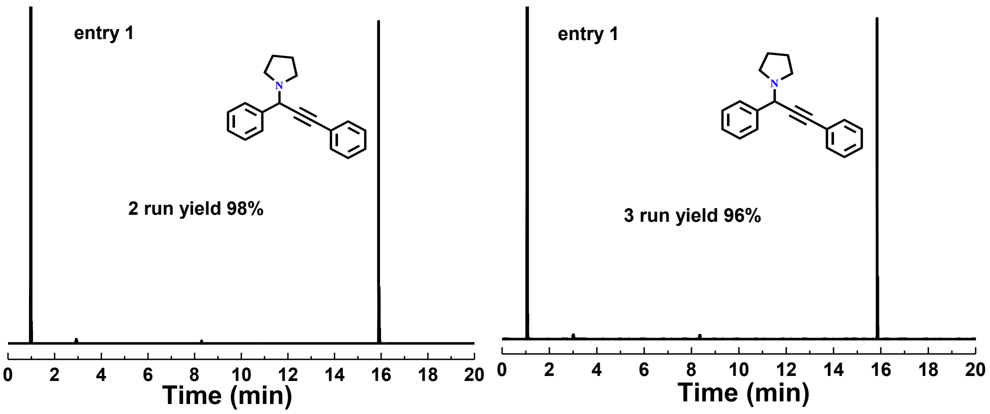

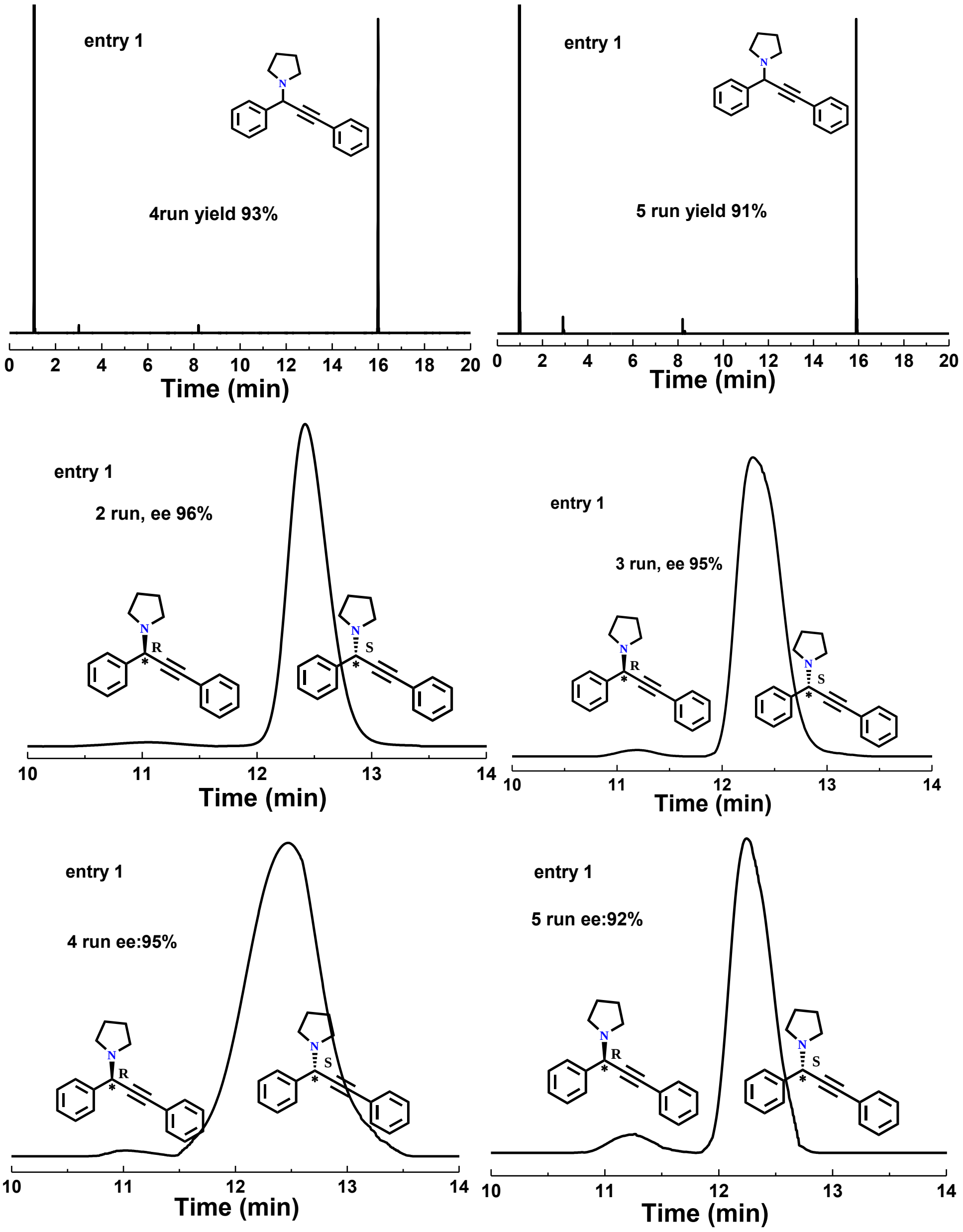

Supplementary Figure 28. Catalytic cycle: GC (yield) and HPLC analysis (ee) for the model one-pot asymmetric A^3-coupling reaction catalyzed by **3**. Yield was determined by the GC on HP-5 column, and enantiomeric excess was determined by HPLC with a Chiralcel OJ-H column (99 : 1 = n-hexane : isopropanol, 1.0 mL min^{-1}, 254 nm).

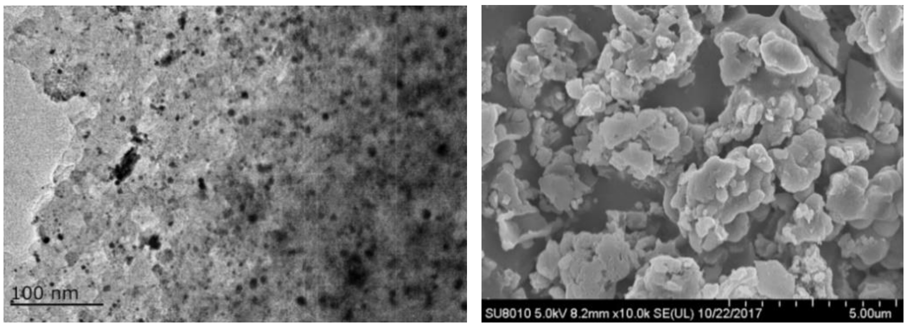

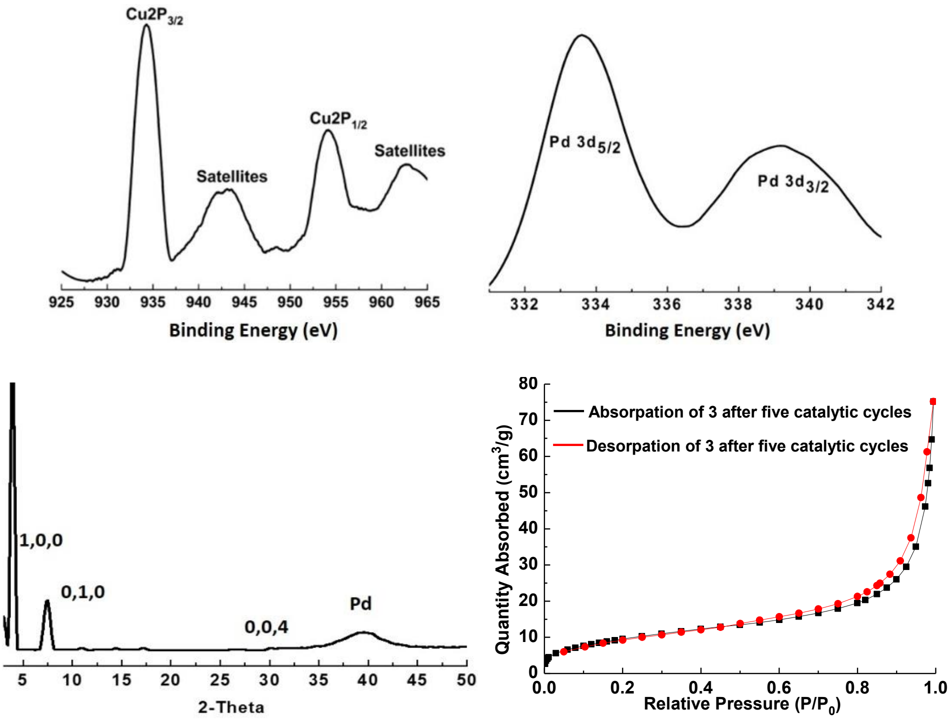

Supplementary Figure 29. TEM, SEM, XPS, PXRD and the gas adsorption-desorption of **3** after five catalytic cycles. The Pd amount in **3** determined by ICP analysis after five catalytic cycles is 20.1 wt %. No structural and morphology changes were observed after five catalytic cycles. The adsorption-desorption value slightly decreased from 94.8 cm^3 g^{-1} (before catalysis) to 75.2 cm^3 g^{-1} for **3** after five cycles.

Supplementary Table 5. Comparison of **3** with the reported catalysts for asymmetric A^3-coupling reactions.

catalyst	conditions	recycle	yield (ee)	TON	ref.
Cu(I)/Acid-Thiourea [a]	CH_2Cl_2/0 °C/12 h	0	92 (92)	23	21
CuBr/(*S*,*S*,*R*a)-**2a** [b]	toluene/0 °C/24 h	0	89 (98.1)	14.8	22
Cu(OTf)/tridentate bis(oxazolinyl)Pybox **5** [c]	toluene/35 °C/2 d	0	83(93)	8.3	23
Cu(I)PF_6/i-Pr-pybox-diPh [d]	toluene/rt/ 5 h	0	95(85)	9.5	24
Cu$(OTf)_2$/PYBOX ligand [e]	DCE/25 °C /15h	0	98(94)	8.2	25
CuBr/(*R*)-quinap-**2** [f]	toluene/rt/70 h	0	98(86)	17.8	26
CuBr/(*R*)-(+)-a-phenethylamine (**5a**) [g]	toluene/rt	0	84(98)	15.3	27
Cu(II)-iPr-pybox-diPh [h]	$CHCl_3$/rt/22 h	0	89(95)	8.9	28
CuBr/chiralligand(*R*)-Quinap [i]	toluene/rt/3 d	3	93(82)	18.6	29
Cu$(OTf)_2$/pybox [j]	ball-milling /30 min	5	91(96)	9.1	30
Me_2Zn/(1*R*,2*S*)-**22i** [k]	CH_2Cl_2/rt/24 h	0	85(80)	2.1	31
$(CuOTf)_2$·toluene/*N*-benzoyl pybim **1b** [l]	CH_2Cl_2/rt/36 h	0	93(98)	9.3	32
CuBr/rac-7 [m]	toluene/0 °C /24 h	0	95(97)	0.95	33
CuBr/rac-StackPhos [n]	CH_2Cl_2/rt/24 h	0	85(94)	15.4	34
CuBr/(*R*,*R*)-1 (PINAP) [o]	toluene/rt/22h	0	88(96)	16	35
**Pd@CCOF-CuTPP (3)** [p]	dioxane/*hv*/ 10 h	4	98(96)	46.7	**this work**

[a] benzaldehyde, pyrrolidine and phenylacetylene
[b] isobutyraldehyde, dibenzylamine and trimethylsilylacetylene
[c] aldehyde, aniline and alkyne

[d] benzaldehyde, aniline and phenylacetylene
[e] aldehyde, phenylacetylene and aniline
[f] phenylacteylene, 3-methylbutanal and dibenzylamine
[g] isobutyraldehyde, $HNBn_2$ and trimethylsilylacetylene
[h] methyl 2-formybenzoate, aniline and alkyne
[i] benzaldehyde, $HNBn_2$ and trimethylsilylacetylene
[j] benzaldehyde, aniline and phenylacetylene
[k] *p*-chlorobenzaldehyde, o-methoxyaniline and phenylacetylene
[l] PhCHO, *p*-anisidine, 4-phenylbutyne
[m] cyclohexanecarboxyaldehyde, $HNBn_2$ and trimethylsilylacetylene
[n] ynal, dibenzylamine and TMS-acetylene
[o] isobutyraldehyde, 4-piperidone·HCl and trimethylsilylacetylene
[p] benzaldehyde, pyrrolidine and phenylacetylene

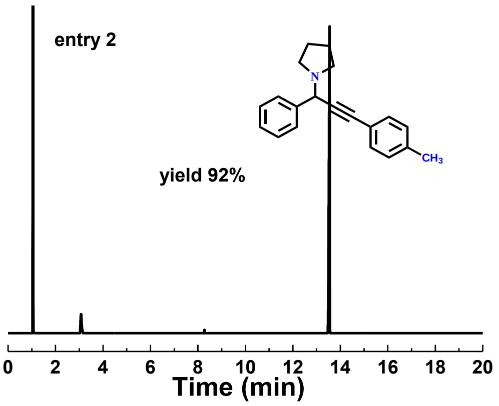

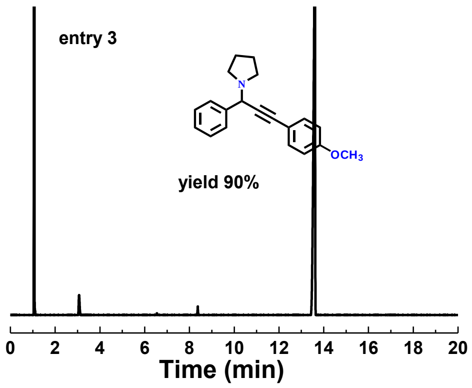

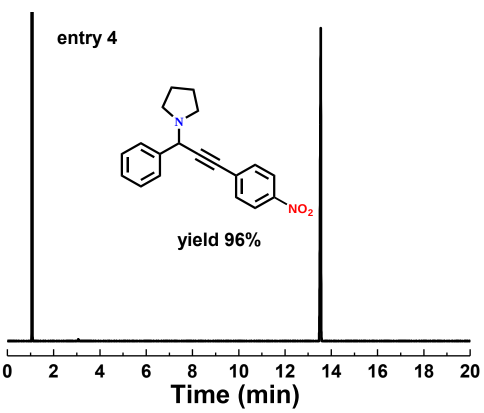

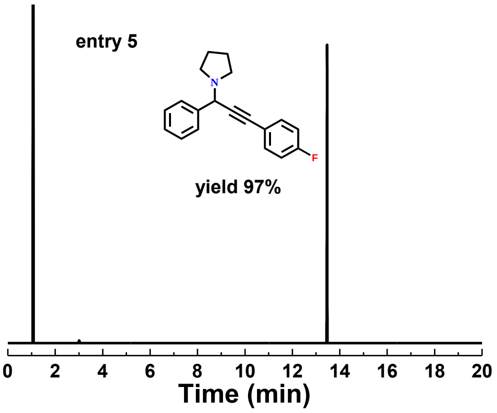

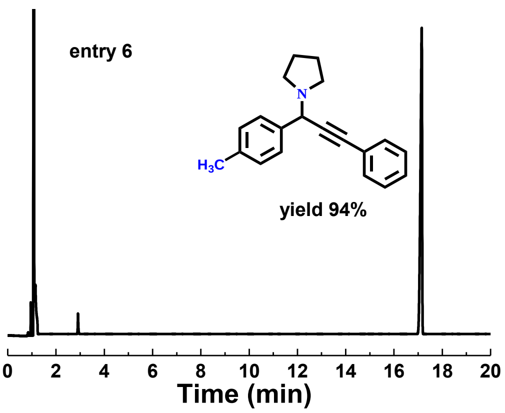

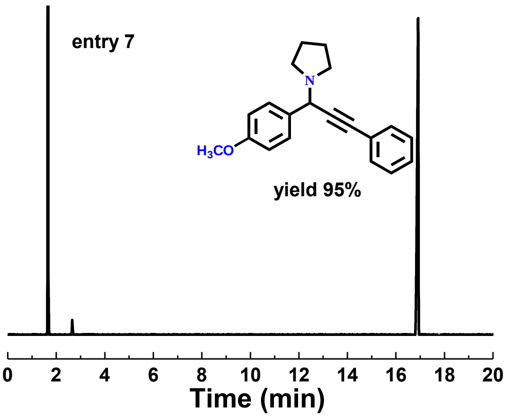

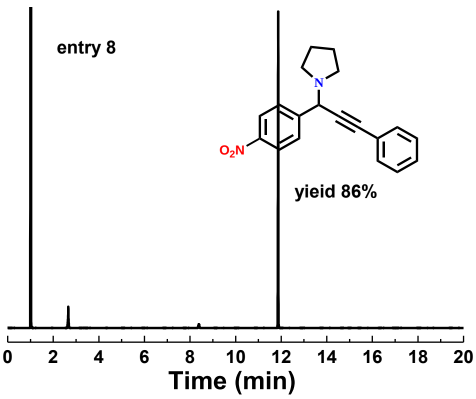
entry 8
N
O2N
yieid 86%
0 2 4 6 8 10 12 14 16 18 20
Time (min)

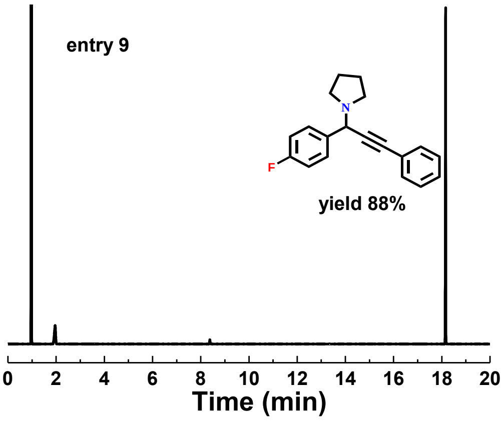
entry 9
N
F
yield 88%
0 2 4 6 8 10 12 14 16 18 20
Time (min)

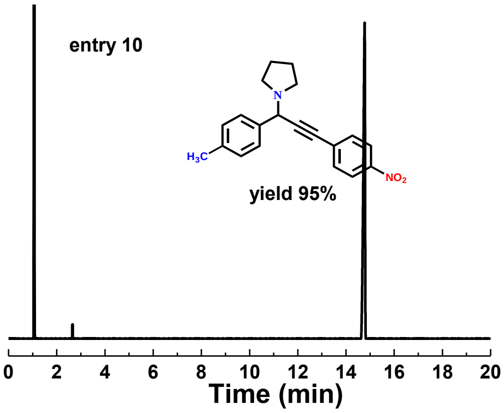
entry 10
N
H3C
NO2
yield 95%
0 2 4 6 8 10 12 14 16 18 20
Time (min)

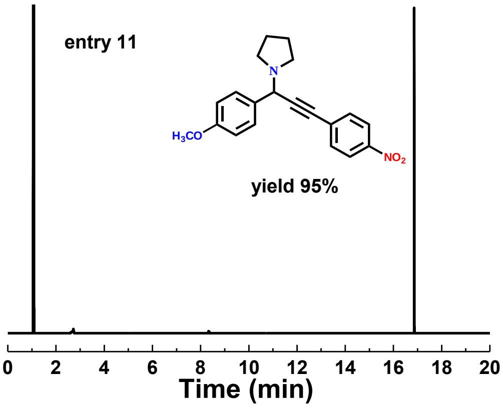
entry 11
N
H3CO
NO2
yield 95%
0 2 4 6 8 10 12 14 16 18 20
Time (min)

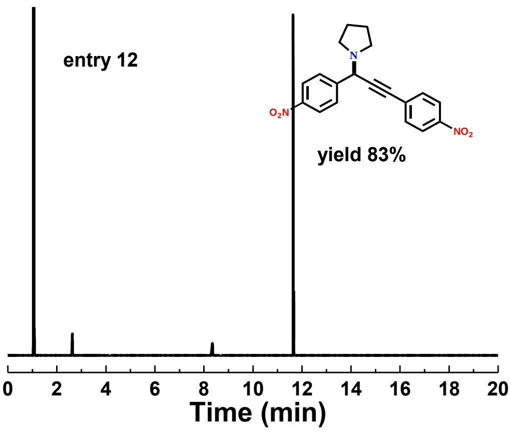
entry 12
N
O2N
NO2
yield 83%
0 2 4 6 8 10 12 14 16 18 20
Time (min)

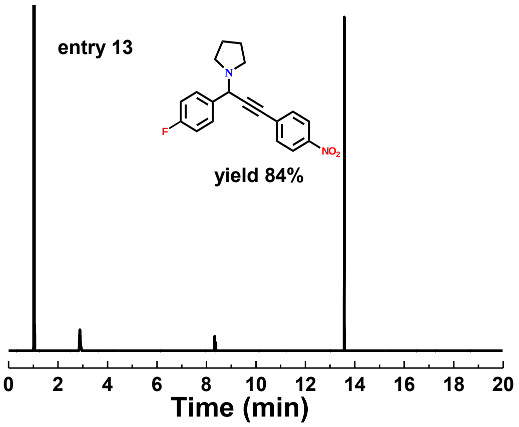
entry 13
N
F
NO2
yield 84%
0 2 4 6 8 10 12 14 16 18 20
Time (min)

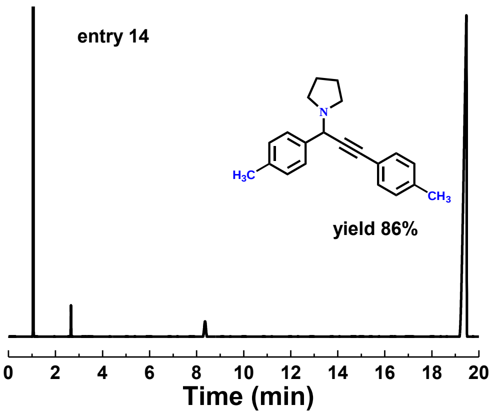
entry 14
N
H3C
CH3
yield 86%
0 2 4 6 8 10 12 14 16 18 20
Time (min)

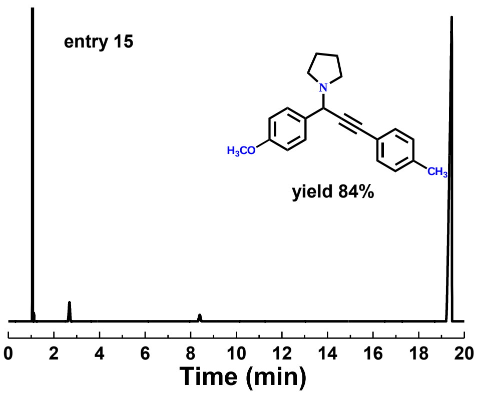
entry 15
N
H3CO
CH3
yield 84%
0 2 4 6 8 10 12 14 16 18 20
Time (min)

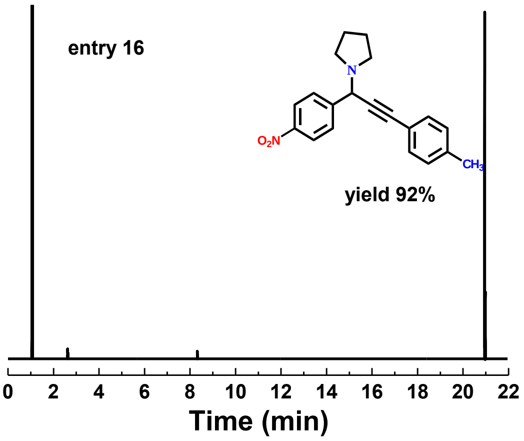
entry 16
O2N
N
CH3
yield 92%
0 2 4 6 8 10 12 14 16 18 20 22
Time (min)

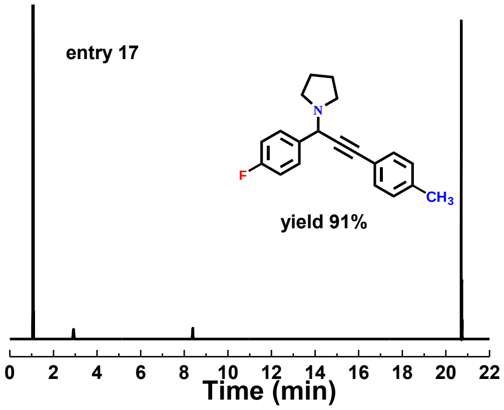
entry 17
N
F
CH3
yield 91%
0 2 4 6 8 10 12 14 16 18 20 22
Time (min)

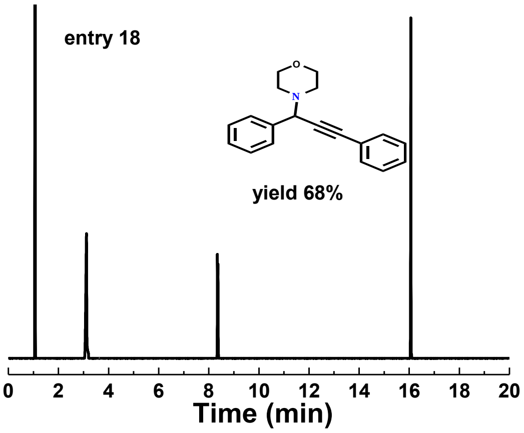
entry 18
O
N
yield 68%
0 2 4 6 8 10 12 14 16 18 20
Time (min)

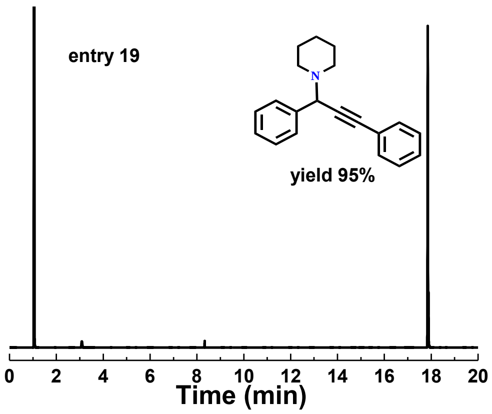
entry 19
N
yield 95%
0 2 4 6 8 10 12 14 16 18 20
Time (min)

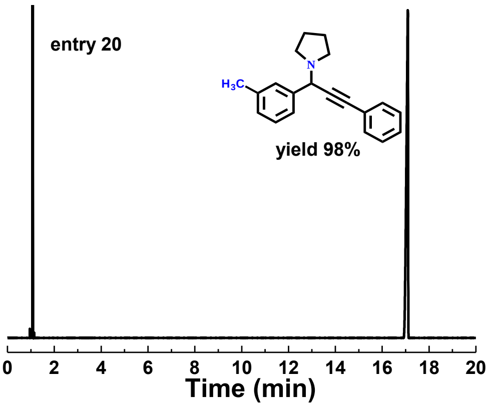
entry 20
H3C
N
yield 98%
0 2 4 6 8 10 12 14 16 18 20
Time (min)

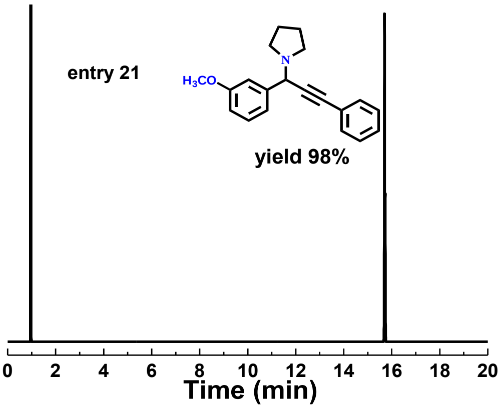
entry 21
H3CO
N
yield 98%
0 2 4 6 8 10 12 14 16 18 20
Time (min)

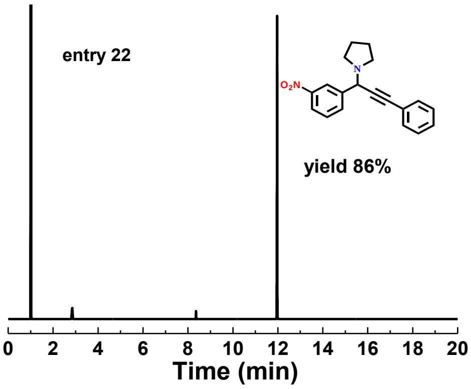
entry 22
O2N
N
yield 86%
0 2 4 6 8 10 12 14 16 18 20
Time (min)

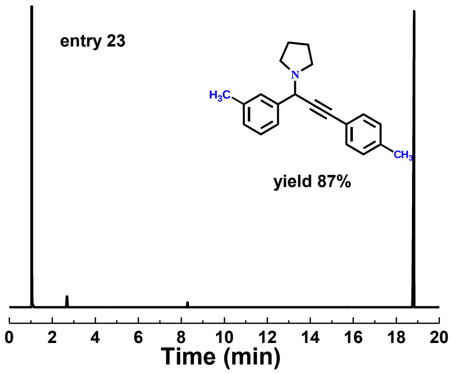
entry 23
H3C
N
CH3
yield 87%
0 2 4 6 8 10 12 14 16 18 20
Time (min)

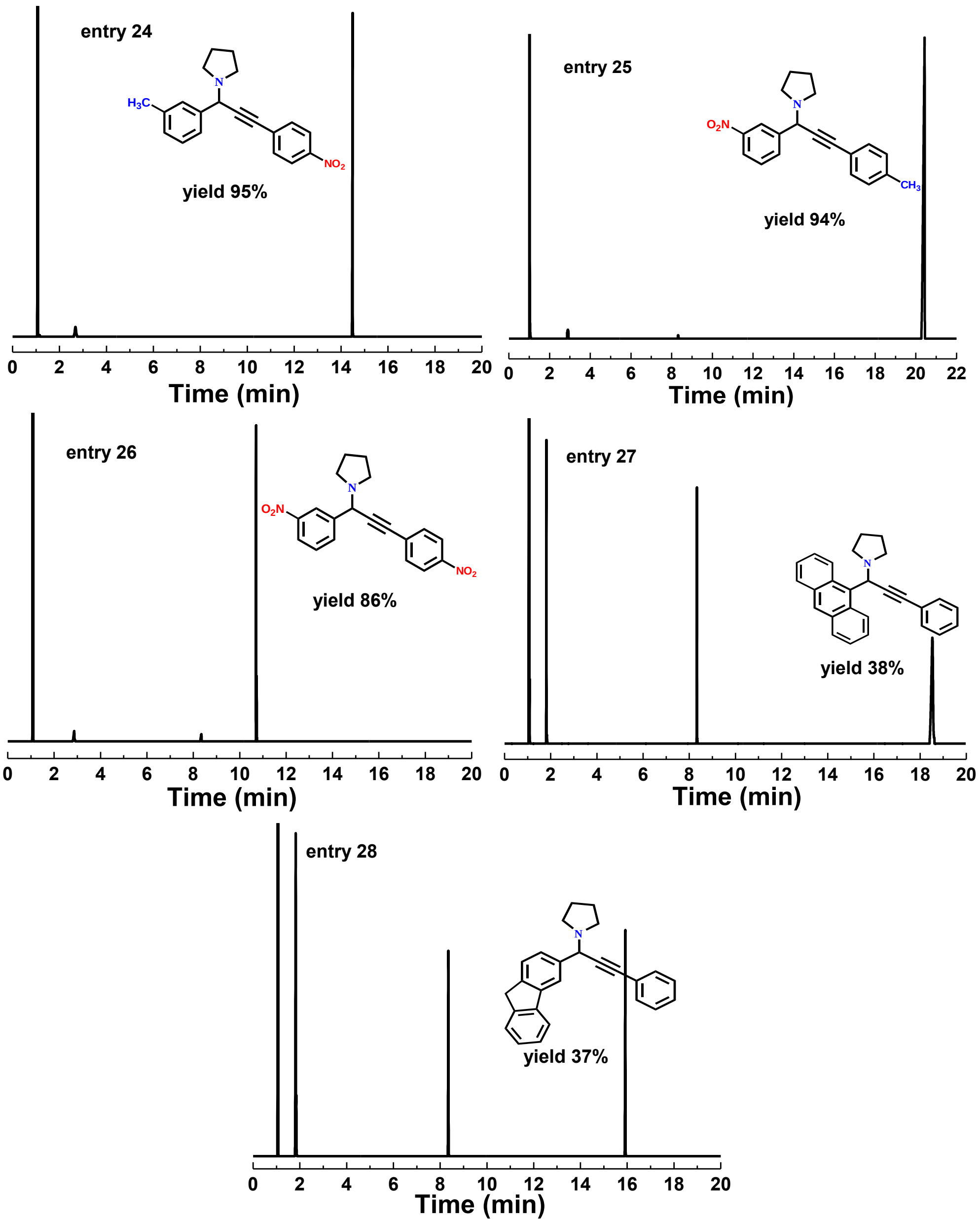

Supplementary Figure 30. GC analysis (yield) for the expanded asymmetric A^3-coupling reactions with various substituted substrates catalyzed by **3** (for Table 4).

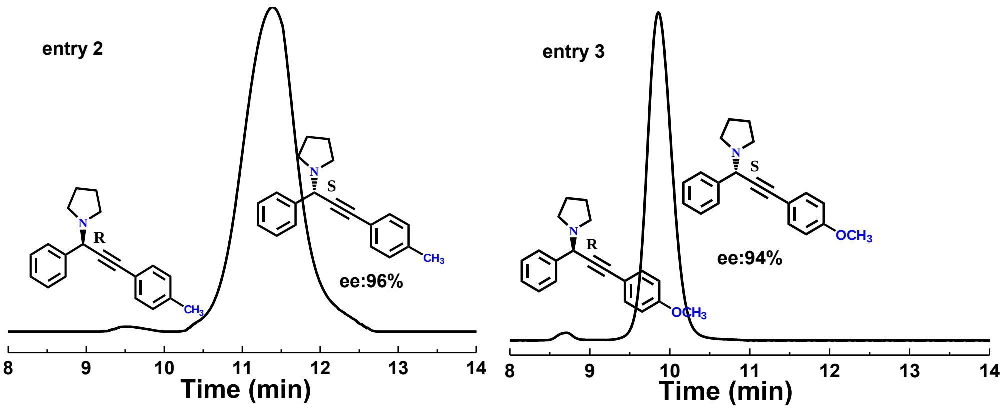

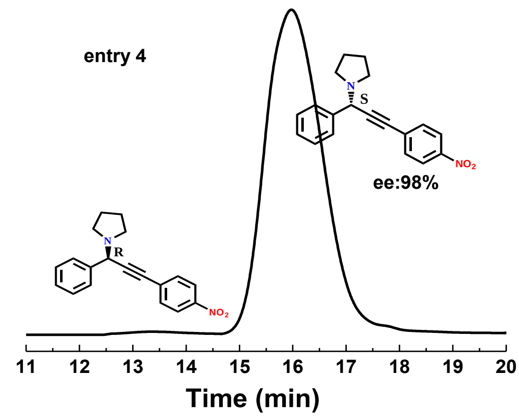
entry 4
ee:98%
Time (min)

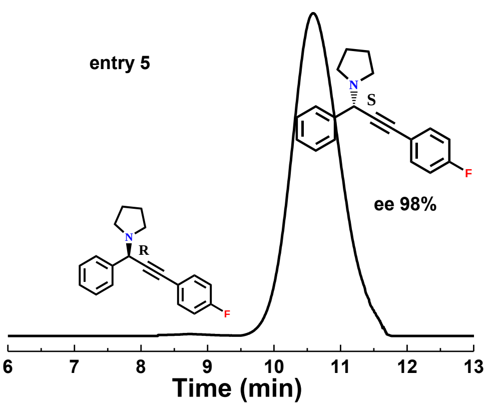
entry 5
ee 98%
Time (min)

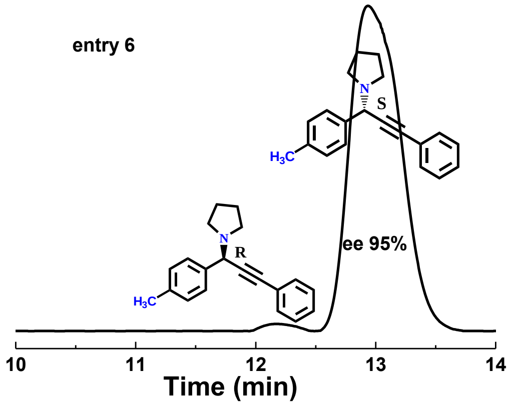
entry 6
ee 95%
Time (min)

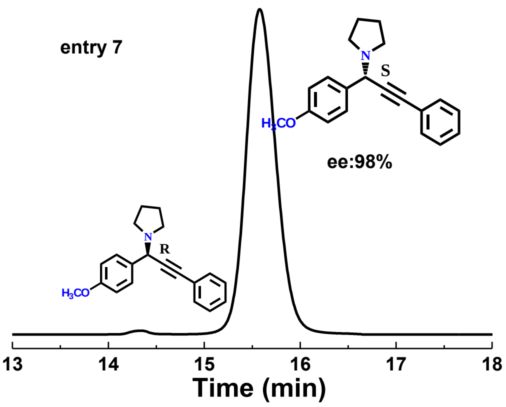
entry 7
ee:98%
Time (min)

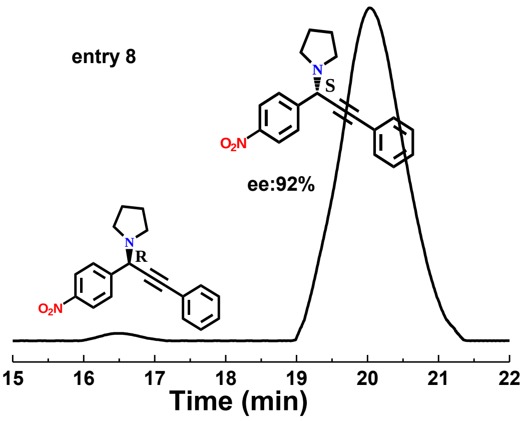
entry 8
ee:92%
Time (min)

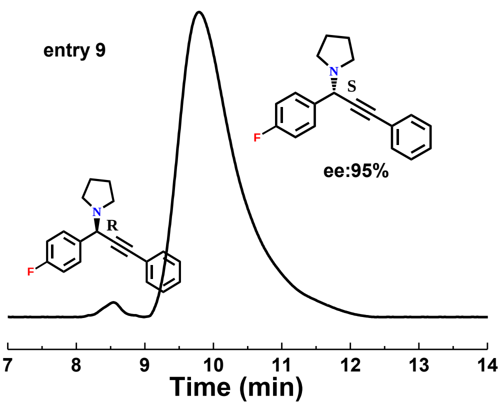
entry 9
ee:95%
Time (min)

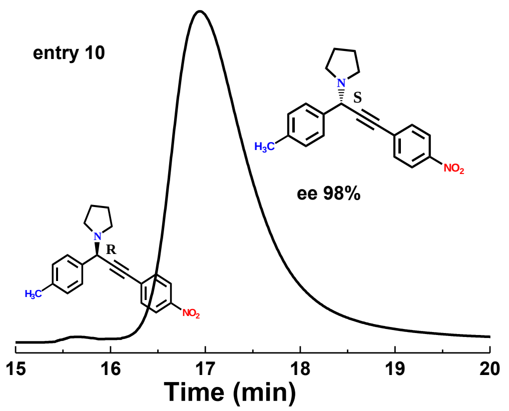
entry 10
ee 98%
Time (min)

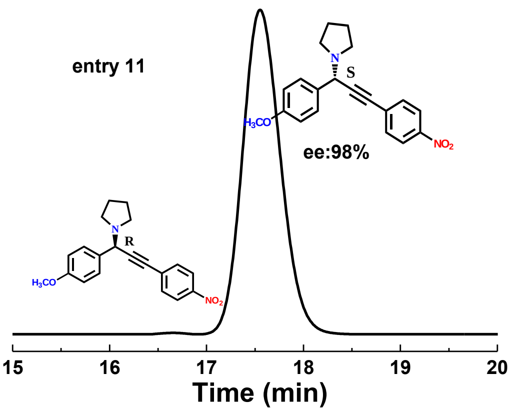
entry 11
ee:98%
Time (min)

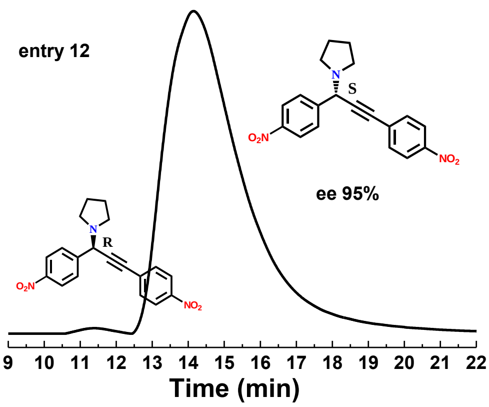
entry 12
ee 95%
9 10 11 12 13 14 15 16 17 18 19 20 21 22
Time (min)

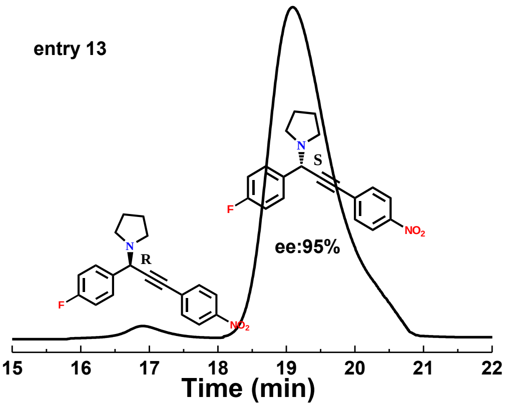
entry 13
ee:95%
15 16 17 18 19 20 21 22
Time (min)

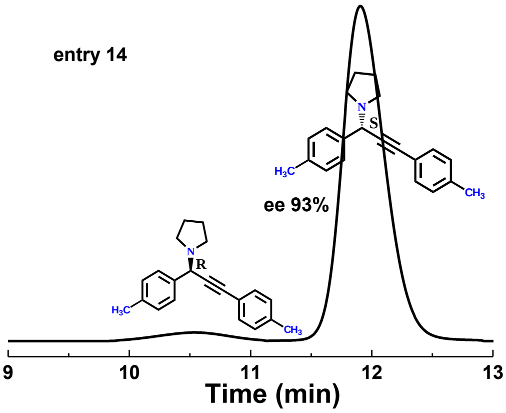
entry 14
ee 93%
9 10 11 12 13
Time (min)

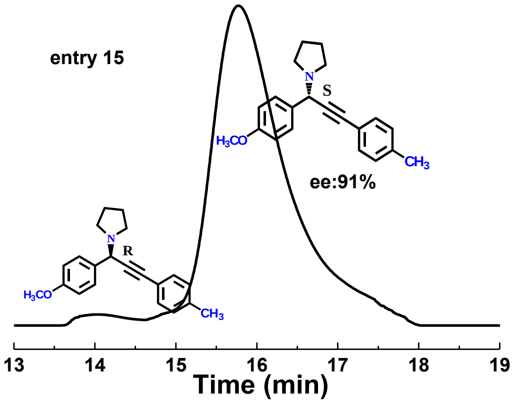
entry 15
ee:91%
13 14 15 16 17 18 19
Time (min)

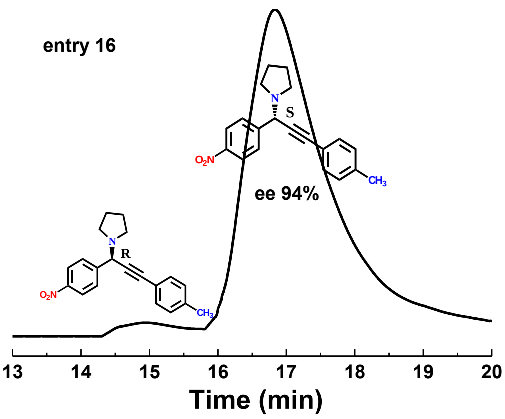
entry 16
ee 94%
13 14 15 16 17 18 19 20
Time (min)

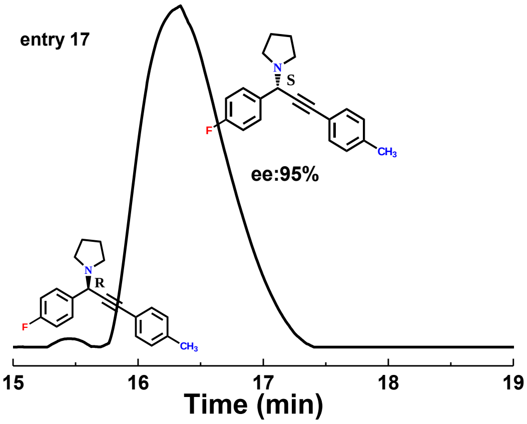
entry 17
ee:95%
15 16 17 18 19
Time (min)

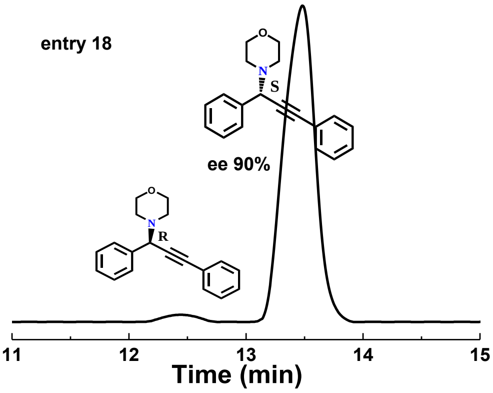
entry 18
ee 90%
11 12 13 14 15
Time (min)

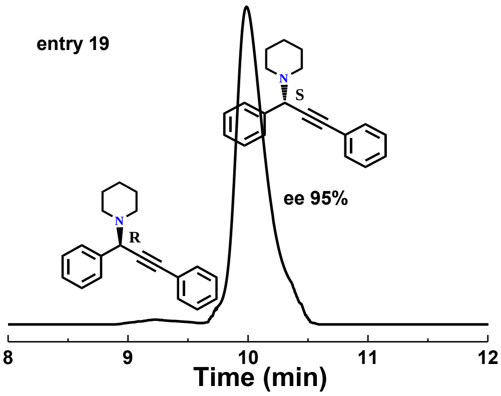
entry 19
ee 95%
8 9 10 11 12
Time (min)

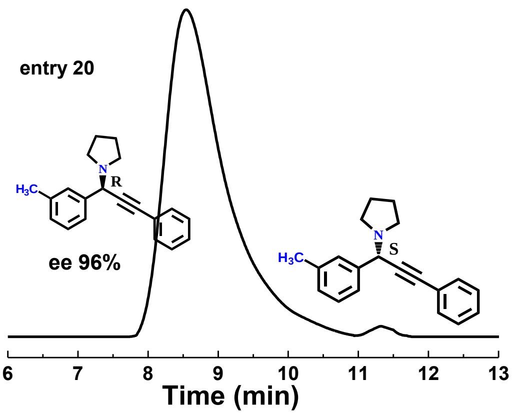
entry 20
ee 96%
R
S
Time (min)

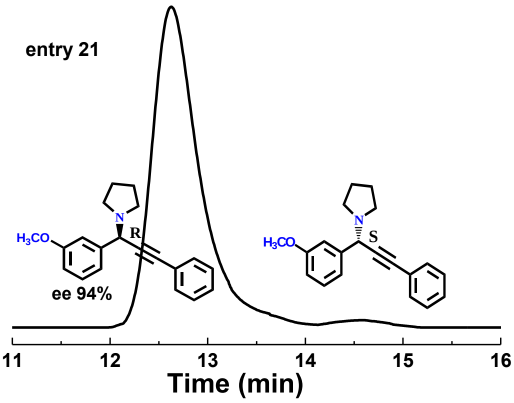
entry 21
ee 94%
R
S
Time (min)

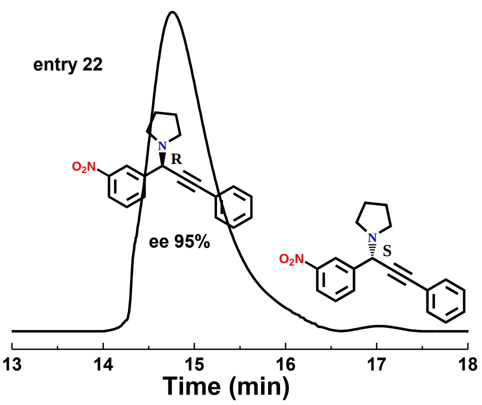
entry 22
ee 95%
R
S
Time (min)

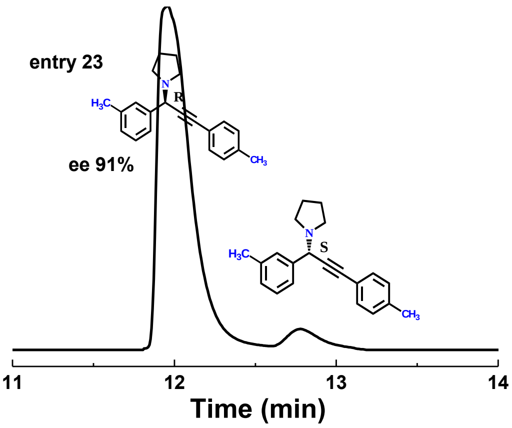
entry 23
ee 91%
R
S
Time (min)

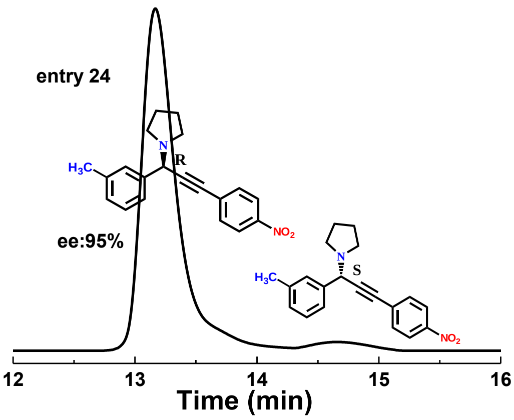
entry 24
ee:95%
R
S
Time (min)

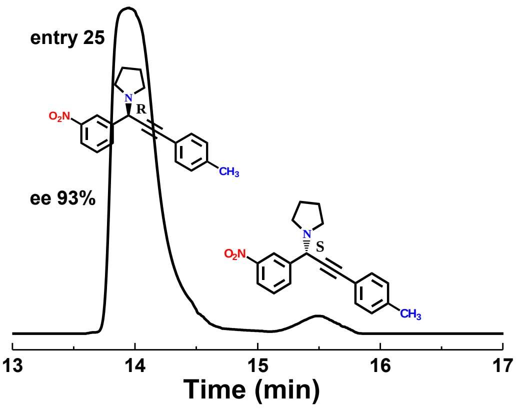
entry 25
ee 93%
R
S
Time (min)

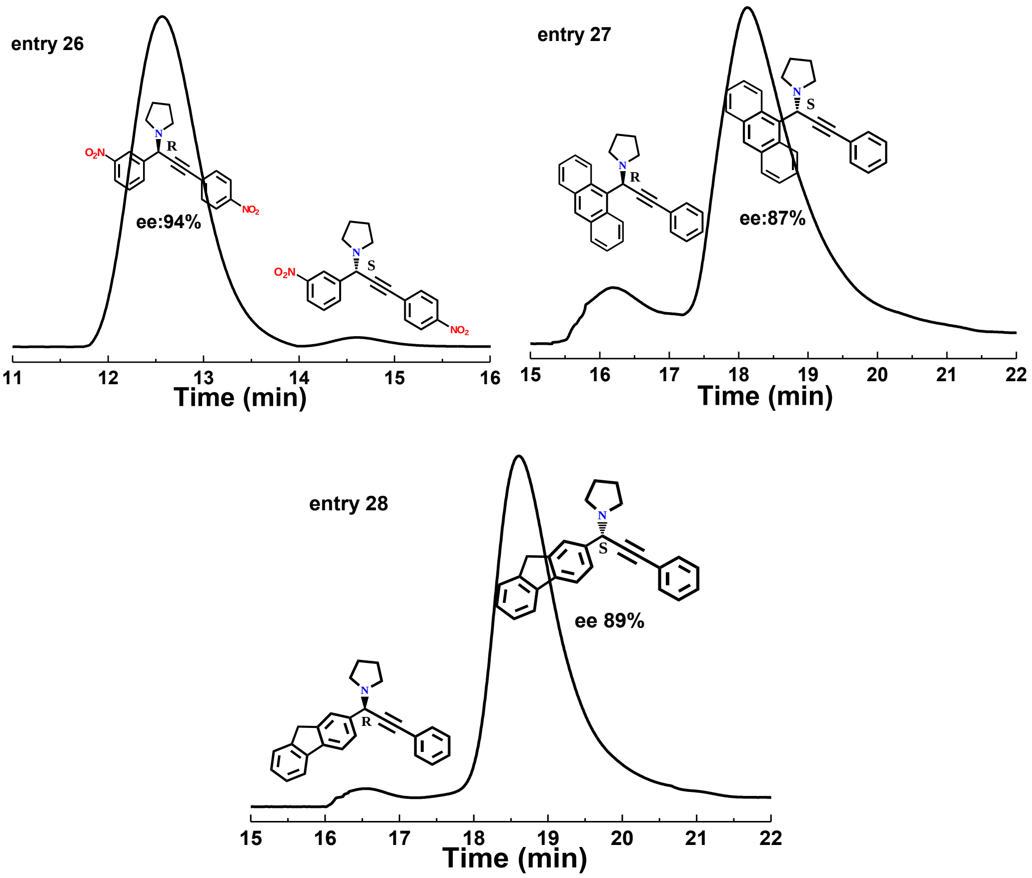

Supplementary Figure 31. HPLC (ee) analysis for the expanded asymmetric A^3-coupling reactions with various substituted substrates catalyzed by **3** (for Table 4). HPLC with a Chiralcel OD-H column was used for the products generated from aromatic aldehyde or aromatic alkyne substrates with electron-withdrawing groups (99 : 1 = *n*-hexane : isopropanol, 1.0 mL min^{-1}, 254 nm). HPLC with a Chiralcel OJ-H column was used for the products generated from other types of aromatic aldehyde or aromatic alkyne substrates (99 : 1 = *n*-hexane : isopropanol, 1.0 mL min^{-1}, 254 nm).

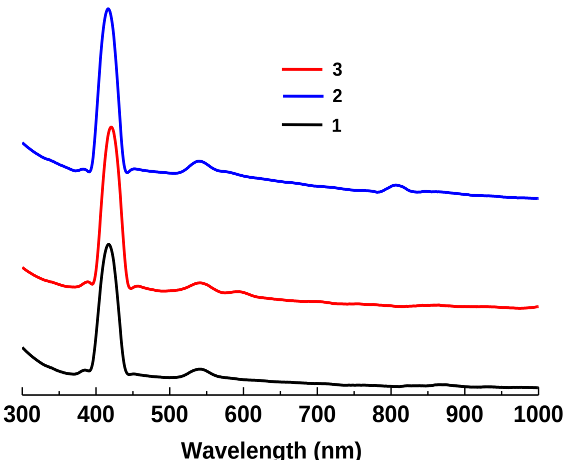

Supplementary Figure 32. Absorption spectra of **1**, **2** and **3**. They all displayed the multiple adsorption bands greater than 400 nm, including porphyrin Soret band (ca. 417 nm) and one or two weak Q-bands (540-702 nm).

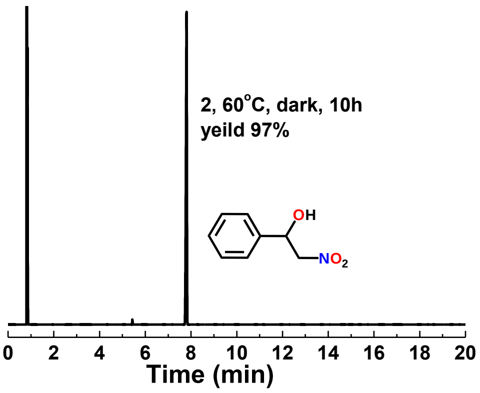
2, 60°C, dark, 10h
yeild 97%
OH
NO2
0 2 4 6 8 10 12 14 16 18 20
Time (min)

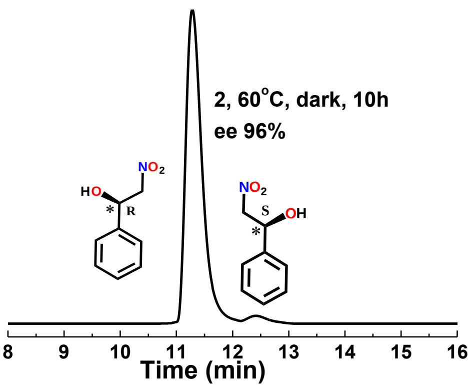
2, 60°C, dark, 10h
ee 96%
NO2
HO
R
S
OH
8 9 10 11 12 13 14 15 16
Time (min)

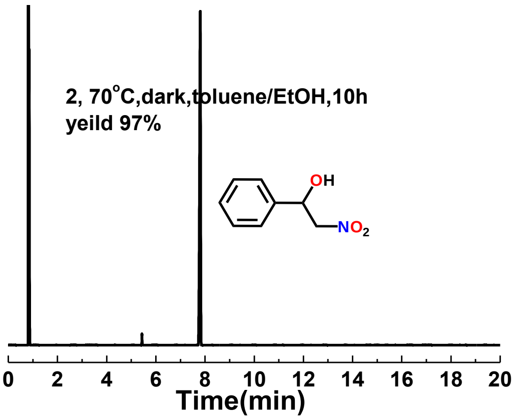
2, 70°C,dark,toluene/EtOH,10h
yeild 97%
OH
NO2
0 2 4 6 8 10 12 14 16 18 20
Time(min)

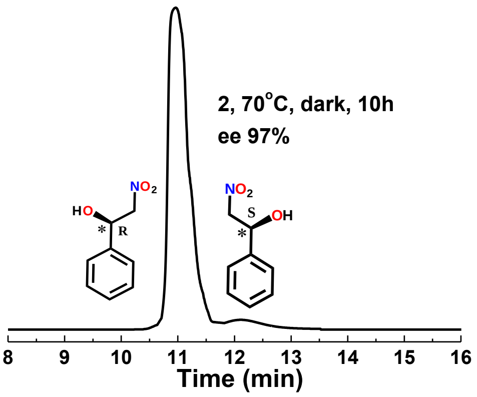
2, 70°C, dark, 10h
ee 97%
NO2
HO
R
S
OH
8 9 10 11 12 13 14 15 16
Time (min)

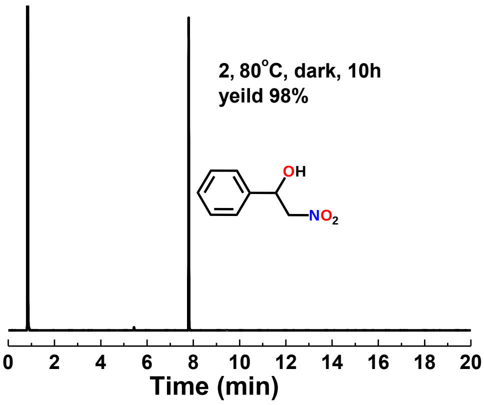
2, 80°C, dark, 10h
yeild 98%
OH
NO2
0 2 4 6 8 10 12 14 16 18 20
Time (min)

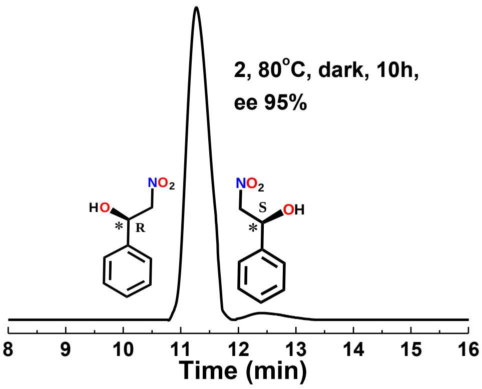
2, 80°C, dark, 10h,
ee 95%
NO2
HO
R
S
OH
8 9 10 11 12 13 14 15 16
Time (min)

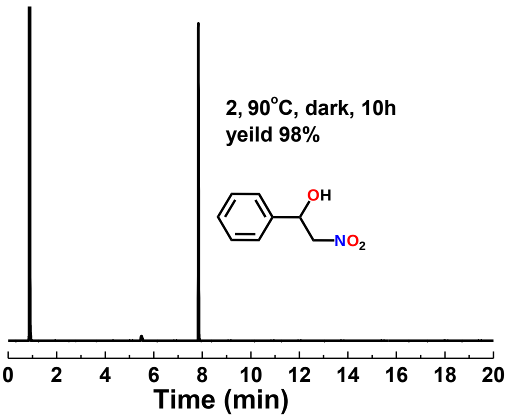
2, 90°C, dark, 10h
yeild 98%
OH
NO2
0 2 4 6 8 10 12 14 16 18 20
Time (min)

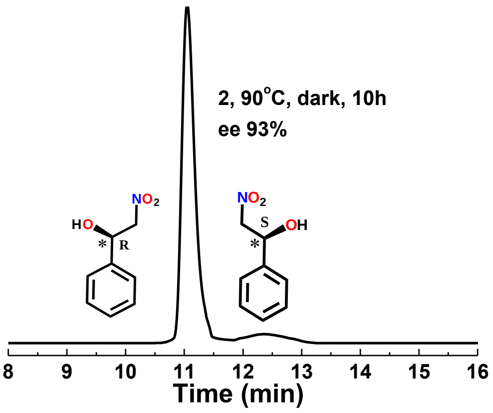
2, 90°C, dark, 10h
ee 93%
NO2
HO
R
S
OH
8 9 10 11 12 13 14 15 16
Time (min)

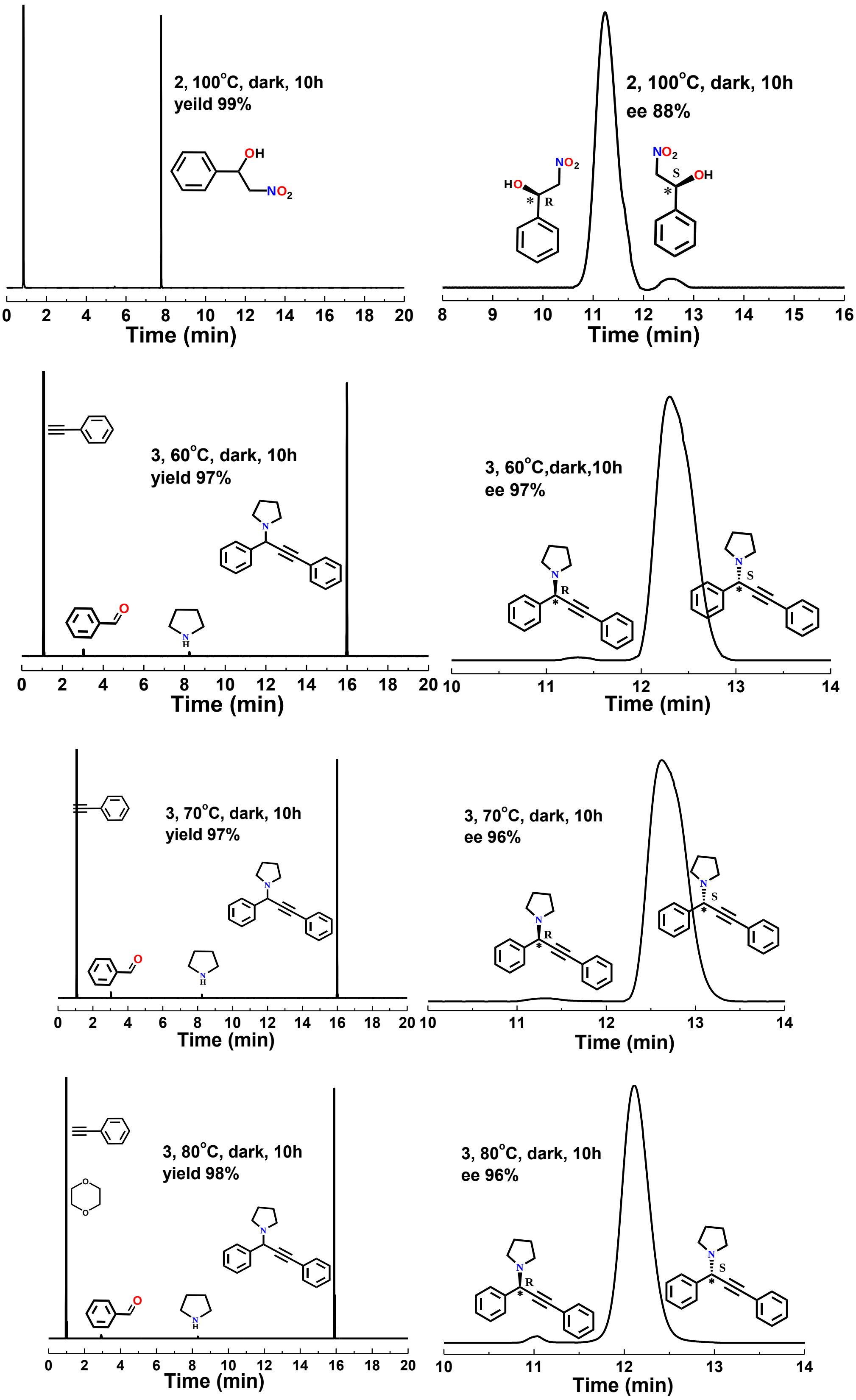

2, 100°C, dark, 10h
yeild 99%
Time (min)
2, 100°C, dark, 10h
ee 88%
Time (min)
3, 60°C, dark, 10h
yield 97%
Time (min)
3, 60°C,dark,10h
ee 97%
Time (min)
3, 70°C, dark, 10h
yield 97%
Time (min)
3, 70°C, dark, 10h
ee 96%
Time (min)
3, 80°C, dark, 10h
yield 98%
Time (min)
3, 80°C, dark, 10h
ee 96%
Time (min)

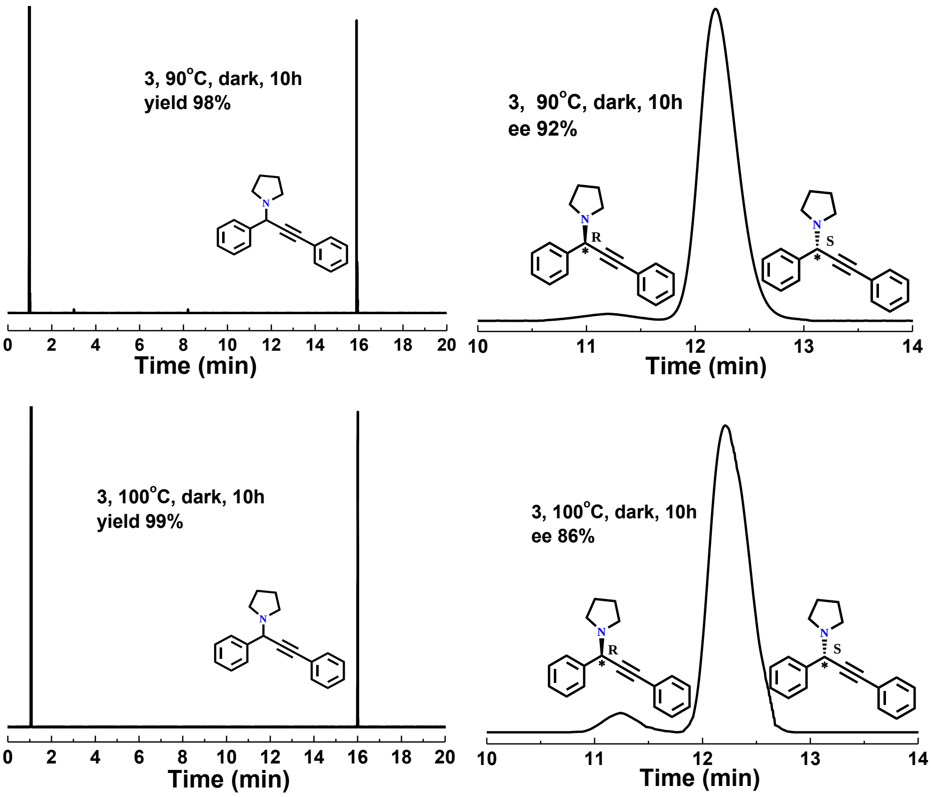

Supplementary Figure 33. Yield and ee for the **2**-catalyzed asymmetric model Henry reaction (**2**, 6.0 mg, 1 mol% Au equiv, benzyl alcohol (0.5 mmol), nitromethane (1.5 mmol), K_2CO_3 (1.5 mmol), PhMe/EtOH (1:1, 2 mL)) and the **3**-catalysed chiral model A^3-coupling reaction (**3**, 4.5 mg, 2.1 mol Pd% equiv, benzaldehyde (0.5 mmol), phenylacetylene (0.5 mmol), pyrrolidine (0.75 mmol), *p*-dioxane (2 mL)) performed at different temperatures in dark (for Table 5).

Supplementary References

(1) Wang, X., et al. Zinc Tetraphenylporphyrin−Fluorene Branched Copolymers: Synthesis and Light-Emitting Properties. *Macromolecules*. **43**, 709-715 (2010).

(2) Mayani, V. J., Abdi, S. H. R., Kureshy, R. I., Khan, N. H., Das, A., Bajaj, H. C. Heterogeneous Chiral Copper Complexes of Amino Alcohol for Asymmetric Nitroaldol Reaction. *J. Org. Chem*. **75**, 6191-6195 (2010).

(3) Noole, A., Lippur, K., Metsala, A., Lopp, M., Kanger. T. Enantioselective Henry Reaction Catalyzed by CuII Salt and Bipiperidine. *J. Org. Chem*. **75**, 1313-1316 (2010).

(4) Evans, D. A., Seidel, D., Rueping, M., Lam, H. W., Shaw, J. T., Downey, C. W. A New Copper Acetate-Bis(oxazoline)-Catalyzed, Enantioselective Henry Reaction. *J. Am. Chem. Soc*. **125**, 12692-12693 (2003).

(5) Li, Y., Deng, P., Zeng, Y., Xiong, Y., Zhou, H. anti-Selective Asymmetric Henry Reaction Catalyzed by a Heterobimetallic Cu−Sm−Aminophenol Sulfonamide Complex. *Org. Lett*. **18**, 1578-1581 (2016).

(6) Yu, X., Pérez, B., Zhang, Z., Gao, R., Guo, Z. Mining catalytic promiscuity from Thermophilic archaea: an acyl-peptide releasing enzyme from Sulfolobus tokodaii (ST0779) for nitroaldolreactions. *Green Chem*. **18**, 2753-2761 (2016).

(7) Kuhbeck, D., Saidulu, G., Reddy, K. R., Dıaz, D. D. Critical assessment of the efficiency of chitosan biohydrogel beads as recyclable and heterogeneous organocatalyst for C–C bond formation. *Green Chem*. **14**, 378-392 (2012).

(8) Lang, K., Park, J., Hong, S. Urea/Transition-Metal Cooperative Catalyst for anti-SelectiveAsymmetric Nitroaldol Reactions. *Angew. Chem. Int. Ed*. **51**, 1620-1624 (2012).

(9) Huang, H., Zong, H., Bian, G., Yue, H., Song, L. Correlating the Effects of the N-Substituent Sizes of Chiral 1,2-Amino Phosphinamide Ligands on Enantioselectivities in Catalytic Asymmetric Henry Reaction Using Physical Steric Parameters. *J. Org. Chem*. **79**, 9455-9464 (2014).

(10) Tanaka, K., Iwashita, T., Yoshida, E.; Ishikawa, T., Otuka, S., Urbanczyk-Lipkowskab, Z., Takahashi, H. Solvent-dependent strong asymmetric amplification in the catalytic enantioselective Henry reaction using the trans-N, N-bis-biphenyl-4-ylmethylcyclohexane-1,2-diamine-CuCl2 complex. *Chem. Commun*. **51**, 7907-7910 (2015).

(11) Kitagaki, S., Uedab, T., Mukai, C. Planar chiral [2.2]paracyclophane-based bis(thiourea) catalyst: application to asymmetric Henry reaction. *Chem. Commun*. **49**, 4030-4032 (2013).

(12) Kanagaraj, K., Suresh, P., Pitchumani, K. Per-6-amino-cyclodextrin as a Reusable Promoter and Chiral Host for Enantioselective Henry Reaction. *Org. Lett*. **12**, 4070-4073 (2010).

(13) White, D. J., Shaw, S. A New Catalyst for the Asymmetric Henry Reaction: Synthesis of β-Nitroethanols in High Enantiomeric Excess. *Org. Lett*. **14**, 6270-6273 (2012).

(14) Xiong, Y., Wang, F., Huang, X., Wen, Y., Feng. X. A New Copper(I)-Tetrahydrosalen-Catalyzed Asymmetric Henry Reaction and Its Extension to the Synthesis of (S)-Norphenylephrine. *Chem. Eur. J*. **13**, 829-833 (2007).

(15) Qin, D. D., et al. Highly Enantioselective Henry Reactions of Aromatic Aldehydes Catalyzed by an Amino Alcohol-Copper(II) Complex. *Chem. Eur. J*. **18**, 10515-10518 (2012).

(16) Choudary, B. M., Ranganath, K. V. S., Pal, U., Kantam, M. L., Sreedhar, B. Nanocrystalline MgO for Asymmetric Henry and Michael Reactions. *J. Am. Chem. Soc*. **127**, 13167-13171 (2005).

(17) Scharnagel, D., et al. The First Modular Route to Core-Chiral Bispidine Ligands and Their Application in Enantioselective Copper(II)-Catalyzed Henry Reactions. *Chem. Eur. J*. **21**, 12488-12500 (2015).

(18) Spangler, K. Y., Wolf, C. Asymmetric Copper(I)-Catalyzed Henry Reaction with an Aminoindanol-Derived Bisoxazolidine Ligand. *Org. Lett*. **11**, 4724-4727 (2009).

(19) Marcelli, T., Haas, R. N. S., Maarseveen, J. H., Hiemstra, H. Asymmetric Organocatalytic Henry Reaction. *Angew. Chem. Int. Ed*. **45**, 929-931 (2006).

(20) Ma, H. C., Kan, J. L., Chen, G. J., Chen, C. X., Dong, Y. B. Pd NPs-Loaded Homochiral Covalent Organic Framework for Heterogeneous Asymmetric Catalysis, *Chem. Mater*. **15**, 6518-6524 (2017).

(21) Zhao, C., Seidel, D. Enantioselective A3 Reactions of Secondary Amines with a Cu(I)/ Acid−Thiourea Catalyst Combination. *J. Am. Chem. Soc*. **137**, 4650-4653 (2015).

(22) Rokade, B. V., Guiry, P. J. Diastereofacial π‑Stacking as an Approach To Access an Axially Chiral P,N-Ligand for Asymmetric Catalysis. *ACS Catal*. **7**, 2334-2338 (2017).

(23) Wei, C., Mague, J. T., Li, C. J. Cu(I)-catalyzed direct addition and asymmetric addition of terminal alkynes to imines. *PNAS*. **101**, 5749-5754 (2004).

(24) Bisai, A., Singh, V. K. Enantioselective One-Pot Three-Component Synthesis of Propargylamines. *Org. Lett*. **8**, 2405-2408 (2006).

(25) Gao, X. T., Gan, C. C., Liu, S. Y., Zhou, F., Wu, H. H., and Zhou, J. Utilization of CO_2 as a C_1 Building Block in a Tandem Asymmetric A^3 Coupling-Carboxylative Cyclization Sequence to 2−Oxazolidinones. *ACS Catal*. **7**, 8588-8593 (2017).

(26) Gommermann, N., Koradin, C., Polborn, K., Knochel, P. Enantioselective, Copper(I)-Catalyzed Three-Component Reaction for the Preparation of Propargylamines. *Angew. Chem. Int. Ed*. **42**, 5763-5766 (2003).

(27) Knöpfel, T. F., Aschwanden, P., Ichikawa, T., Watanabe, T., Carreira, E. M. Readily Available Biaryl P,N Ligands for Asymmetric Catalysis. *Angew. Chem. Int. Ed*. **43**, 5971-5973 (2004).

(28) Bisai, V., Suneja, A., Singh, V. K. Asymmetric Alkynylation/Lactamization Cascade: An Expeditious Entry to Enantiomerically Enriched Isoindolinones. *Angew. Chem. Int. Ed*. **53**, 10737-10741 (2014).

(29) Gommermann, N., Knochel, P. Practical highly enantioselective synthesis of terminal propargylamines. An expeditious synthesis of (S)-(1)-coniine. *Chem. Commun*. 2324- 2325 (2004).

(30) Li, Z., Jiang, Z., and Su, W. Fast, solvent-free, highly enantioselective three-component coupling of aldehydes, alkynes, and amines catalysed by the copper(II)pybox complex under high-vibration ball-milling. *Green Chem*. **17**, 2330-2334 (2015).
(31) Zani, L., Eichhorn, T., Bolm, C. Dimethylzinc-Mediated, Enantioselective Synthesis of Propargylic Amines. *Chem. Eur. J.* **13**, 2587-2600 (2007).
(32) Nakamura, S., Ohara, M., Nakamura, Y., Shibata, N., Toru, T. Copper-Catalyzed Enantioselective Three-Component Synthesis of Optically Active Propargylamines from Aldehydes, Amines, and Aliphatic Alkynes. *Chem. Eur. J*. **16**, 2360-2362 (2010).
(33) Cardoso, F. S. P., Abboud, K. A., Aponick, A. Design, Preparation, and Implementation of an Imidazole-Based Chiral Biaryl P,N-Ligand for Asymmetric Catalysis. *J. Am. Chem. Soc*. **135**, 14548-14551 (2013).
(34) Paioti, P. H. S., Abboud, K. A., Aponick, A. Catalytic Enantioselective Synthesis of Amino Skipped Diynes. *J. Am. Chem. Soc*. **138**, 2150-2153 (2016).
(35) Aschwanden, P., J. Stephenson, C. R., Carreira, E. M. Highly Enantioselective Access to Primary Propargylamines: 4-Piperidinone as a Convenient Protecting Group. *Org. Lett*. **8**, 2437-2440 (2006).
